# Studies towards the Design and Synthesis of Novel 1,5-Diaryl-1*H*-imidazole-4-carboxylic Acids and 1,5-Diaryl-1*H*-imidazole-4-carbohydrazides as Host LEDGF/p75 and HIV-1 Integrase Interaction Inhibitors

**DOI:** 10.3390/molecules26206203

**Published:** 2021-10-14

**Authors:** Thompho J. Rashamuse, Muhammad Q. Fish, E. Mabel Coyanis, Moira L. Bode

**Affiliations:** 1 Advanced Materials Division, Mintek, Private Bag X3015, Randburg 2194, South Africa; jasonr@mintek.co.za (T.J.R.); QasimF@mintek.co.za (M.Q.F.); 2 Molecular Sciences Institute, School of Chemistry, University of the Witwatersrand, Private Bag 3, PO Wits, Johannesburg 2050, South Africa

**Keywords:** ethyl 1,5-diaryl-1*H*-imidazole-4-carboxylates, 1,5-diaryl-1*H*-imidazole-4-carboxylic acids, 1,5-diaryl-1*H*-imidazole-4-carbohydrazides, ethyl isocyanoacetate, *N*-aryl-benzimidoyl chlorides, HIV-1 integrase, LEDGF/p75

## Abstract

Two targeted sets of novel 1,5-diaryl-1*H*-imidazole-4-carboxylic acids **10** and carbohydrazides **11** were designed and synthesized from their corresponding ester intermediates **17**, which were prepared via cycloaddition of ethyl isocyanoacetate **16** and diarylimidoyl chlorides **15**. Evaluation of these new target scaffolds in the AlphaScreen^TM^ HIV-1 IN-LEDGF/p75 inhibition assay identified seventeen compounds exceeding the pre-defined 50% inhibitory threshold at 100 µM concentration. Further evaluation of these compounds in the HIV-1 IN strand transfer assay at 100 μM showed that none of the compounds (with the exception of **10a**, **10l**, and **11k**, with marginal inhibitory percentages) were actively bound to the active site, indicating that they are selectively binding to the LEDGF/p75-binding pocket. In a cell-based HIV-1 antiviral assay, compounds **11a**, **11b**, **11g**, and **11h** exhibited moderate antiviral percentage inhibition of 33–45% with cytotoxicity (CC_50_) values of >200 µM, 158.4 µM, >200 µM, and 50.4 µM, respectively. The antiviral inhibitory activity displayed by **11h** was attributed to its toxicity. Upon further validation of their ability to induce multimerization in a Western blot gel assay, compounds **11a**, **11b**, and **11h** appeared to increase higher-order forms of IN.

## 1. Introduction

The human immunodeficiency virus type 1 (HIV-1), the causative agent of acquired immune deficiency syndrome (AIDS), is one of the world’s greatest health and development challenges. Despite chemotherapeutic interventions for the virus being developed through active research, the emergence of highly drug-resistant strains against the current approved Food and Drug Administration (FDA) antiretroviral drugs takes a significant toll on the global fight against HIV-1 [1,2,3,4,5,6,7]. Thus, there has been an ongoing search for new treatment options targeting novel steps in the HIV-1 replication cycle. Targeting the active host protein and HIV-1 protein interactions has become a viable therapeutic approach. A typical example is a lens epithelium-derived growth factor (LEDGF/p75) and HIV-1 integrase (IN) protein-protein interaction [8,9,10]. IN is one of three multifaceted essential enzymes required for HIV-1 replication. This 32 kDa protein consists of 288 amino acids and is composed of the catalytic core, *N*-terminal and *C*-terminal functional domains [11,12,13,14]. The main protein function is to allow reliable integration of viral DNA genomes into host cells [11,15]. There are currently only four IN inhibitors on the market that have been clinically tested and approved by the FDA, including elvitegravir, dolutegravir, raltegravir, and bictegravir [16,17,18,19]. These inhibitors are referred to as strand transfer inhibitors (INSTIs) and share the identical mechanistic mode of action of blocking the strand transfer step through binding to the active site of viral IN [14,15,16,17]. Despite the success of highly active antiretroviral therapy (HAART), a new generation of drugs is needed to overcome multi-drug resistance, enhance adherence, and lower toxicities [1,2,3,4,5,6,7].

**Figure 1:**
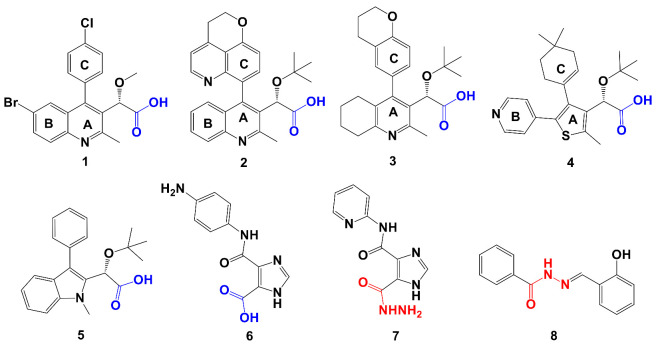
Structures of different of LEDGF/p75-IN inhibitors (**1**–**8**).

During the last two decades, there have been significant efforts to develop small molecules as clinical alternatives to INSTIs for treating HIV-1 infected individuals [20,21,22,23,24,25,26]. These compounds are often referred to as allosteric integrase inhibitors (ALLINIs) or LEDGF/p75-IN inhibitors (LEDGINs), IN-LEDGF/p75 allosteric inhibitors (INLAIs), non-catalytic site integrase inhibitors (NCINIs), and selective multimeric IN inhibitors (MINIs) [23,24,25,26]. They bind to LEDGF/p75 binding sites on IN, leading to aberrant multimerization of HIV-1 IN, which ultimately results in defective virions [16,17,18,19,20,21,22,23,24,25]. Examples include BI-1001 (**1**), BI 224436 (**2**), MUT 101 (**3**), MUT-A (**4**), indole derivatives (**5**), 5-carbonyl-1*H*-imidazole-4-carboxamides (**6** and **7**), and *N*-acylhydrazone-based derivatives (**8**) (Figure 1) [23,24,25,26,27,28,29]. Although none of the LEDGF/p75-IN inhibitors reported to date have been approved clinically, they provide proof-of-concept that developing small molecules targeting the LEDGF/p75-IN interaction is an attractive prospect for new antiviral drug discovery.

**Figure 2:**
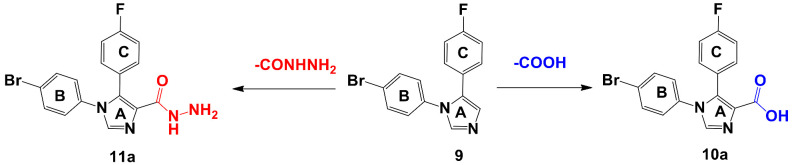
Modification of hit compound **9** by including a carboxylic acid (**10a**) or carbohydrazide group (**11a**).

Research efforts by our group directed towards the identification of potential LEDGF/p75-IN inhibitors led to the discovery of 1,5-diaryl-1*H*-imidazole **9** as a hit compound (Figure 2) [30]. While the candidate hit compound **9** could not achieve antiviral effects in vitro, it contained the common *N*-heterocycle core (**A**) and two aromatic rings (**B** and **C**) present in inhibitors of the HIV-1 IN-LEDGF/p75 interaction.

Evaluation of LEDGF/p75-IN interaction inhibitors **1–6** has shown that the carboxylic acid (-COOH) functionality present in these structures contributes significantly to their binding properties [23,24,25,26,27,28]. Additionally, compounds **7** and **8**, containing a carbohydrazide moiety, have been shown to inhibit the LEDGF/p75-IN interaction [28,29,31]. Based on this information, we postulated that addition of a carboxylic acid functionality or a carbohydrazide motif to the hit compound **9**, to give **10a** and **11a**, respectively, could significantly improve the properties of the parent scaffold (Figure 2).

The 1,5-disubstituted imidazole-4-carboxylate esters are ubiquitous structural motifs in many compounds displaying biological properties [32,33]. One of the efficient methods for making such 1,5-disubstituted imidazole-4-carboxylates would be their direct formation from commercially available α-isocyanoacetates. These α-isocyanoacetates are useful multipurpose reagents for the construction of various nitrogen-containing heterocycles [34,35]. Different five-membered nitrogen-containing heterocycles like thiazole [36,37], imidazole [31,32,33,38,39,40,41,42,43], indole [44,45,46], oxazole [47], pyrrole [48,49], and triazole [50,51] have been built using these synthons. Isocyanoacetate building blocks have attracted the interest of researchers owing to their structural characteristics, reactivity, and reliability. Their structures encompass four densely packed reaction sites, namely the isocyanide motif, α-acidic methine centre, a substituted R-group (which allows for considerable variability), and the carboxylate ester group [40,41]. These reactive sites permit a diverse range of organic transformations, affording α-adducts.

**Scheme 1:**
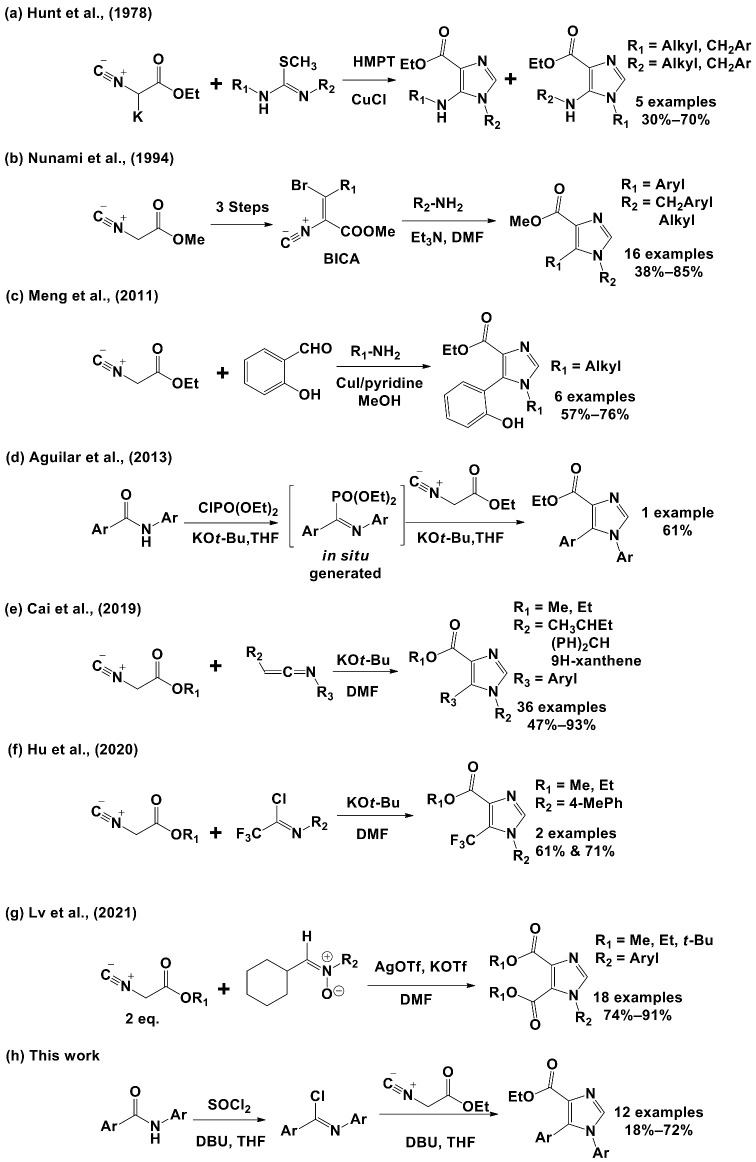
Synthesis of 1,5-disubstituted-imidazole-4-carboxylate esters using α-isocyanoacetates.

A survey of the literature reveals that a few synthetic methods for constructing 1,5-disubstituted-imidazole-4-carboxylates using α-isocyanoacetate synthons have been reported. One of the first syntheses was described in 1978 by Hunt and co-workers [38], where the condensation of the potassium salt of ethyl isocyanoacetate with isothioureas in hexamethylphosphoric triamide (HMPT) at room temperature catalyzed by copper (I) chloride (CuCl) afforded a mixture of disubstituted imidazole-4-carboxylate esters in various yields as presented in Scheme 1a. Another approach was reported in 1994 by Nunami and co-workers [39] in which key intermediate 3-bromo-2-isocyanoacrylates (BICA) were easily derived from isocyanoacetate esters, followed by reaction with a variety of primary amines in the presence of triethylamine (Et_3_N) in *N*,*N*-dimethylformamide (DMF) as solvent (Scheme 1b). Furthermore, in 2011, Meng and co-workers [33] demonstrated a strategy to introduce a range of 1,5-disubstituted-1*H*-imidazole-4-carboxylate ester derivatives as intermediates towards the synthesis of chromeno [3,4-*d*]imidazol-4-one by using readily available ethyl isocyanoacetate, primary aliphatic amines, and an aldehyde in the presence of copper iodide (CuI) and pyridine in methanol (MeOH) heated at 50 °C (Scheme 1c). Aguilar and co-workers [40] reported a one-pot two-step strategy for the preparation of a disubstituted imidazole-4-carboxylate ester. This procedure involves first coupling 4-chloroaniline with 4-iodobenzoyl chloride using 1-ethyl-(3-dimethylaminopropyl)carbodiimide (EDCI) to produce the amide. The amide is then treated with chlorophosphate to generate unstable iminophosphates in situ, followed by condensation under basic conditions with ethyl isocyanoacetate to produce the desired compound (Scheme 1d). This procedure only reported one example and the approach required strictly anhydrous conditions. Additionally, Cai and co-workers [41] in 2019 reported an annulation between α-isocyanoacetates and ketenimines in the presence of a base, potassium *tert*-butoxide (*t*-BuOK), in DMF at 40 °C as an efficient and straightforward synthesis of disubstituted imidazole-4-carboxylate ester derivatives in good yields (Scheme 1e). Additionally, Hu and co-workers [42] in 2020 reported a new method for preparing 1,5-disubstituted imidazole-4-carboxylate esters by *t*-BuOK-mediated annulation of ester isocyanoacetates with trifluoroacetimidoyl chlorides (Scheme 1f). The methodology includes mild reaction conditions and uses readily available reagents. However, only two examples were reported utilizing ester isocyanoacetate synthons in this approach. More recently, Lv and co-workers [43] in 2021 described the construction of these compounds in good yields by reacting α-isocyanoacetates with nitrones catalyzed by silver trifluoromethanesulfonate (AgOTf) in the presence of potassium trifluoromethanesulfonate (KOTf) in toluene (PhMe) as a solvent under a nitrogen atmosphere (Scheme 1g).

As part of our ongoing interest in the design of possible inhibitors of the HIV-1 IN and LEDGF/p75 protein-protein interaction [30], we directed our attention to the synthesis of 1,5-disubstitued-1*H*-imidazole-4-carboxylate esters. Herein we describe a novel and efficient approach for the synthesis of diaryl-imidazole-4-carboxylate esters via cycloaddition of an α-isocyanoacetate with stable *N*-aryl-benzimidoyl chloride intermediates (Scheme 1h). None of the previously reported synthetic methods to these compounds [33,40,41,42,43] make use of diarylimidoyl chlorides and isocyanoacetate esters. The carboxylate esters prepared using this new methodology were subsequently converted into the corresponding 1,5-diaryl-1*H*-imidazole-4-carboxylic acids and 1,5-diaryl-1*H*-imidazole-4-carbohydrazides, which were then evaluated for their ability to bind to the novel HIV-1 IN dimer interface pocket.

## 2. Results and Discussion

### 2.1. Testing the Hypothesis In Silico

**Figure 3:**
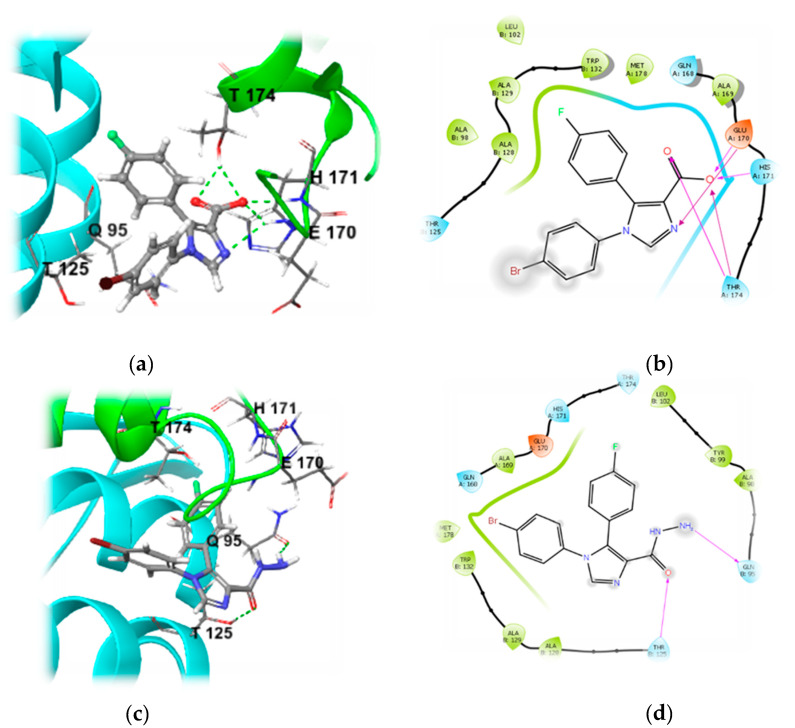
Docking studies: (**a**) 3D model of structure **10a** docked at the LEDGF/p75 binding site in HIV-1 integrase (PDB code: 4NYF, chain A: green and chain B: light blue); (**b**) 2D view of structure **10a** at the LEDGF/p75 binding site in HIV-1 integrase; (**c**) the 3D mode of compound **11a** binding at the LEDGF/p75 binding site in HIV-1 integrase; (**d**) 2D image of structure **11a** at the LEDGF/p75 binding site in HIV-1 integrase. Hydrogen bonds are represented by the green dotted lines in 3D structures (**a**,**c**) and as purple arrows in 2D views (**b**,**d**).

In order to test our hypothesis and provide some proof of concept for preparation of the proposed analogues (Figure 2), target candidates **10a** and **11a** were docked into the LEDGF/p75-binding pocket of HIV-1 integrase to gain a deeper understanding of the possible binding interactions. The X-ray crystal structure of HIV-1 IN dimer (PDB code: 4NYF) in complex with the small molecule, BI 224436 (**2**), was used for a better understanding of binding interactions of the tested ligands [27]. The model developed suggested a clear difference in the positioning of the two ligands within the binding site. The carboxylic acid moiety of compound **10a** was shown to form hydrogen bonds with critical histidine (His) 171, glutamic acid (Glu) 170, and threonine (Thr) 174 amino acid residues of HIV-1 IN, while the imidazole motif is predicted to exhibit hydrogen bonding with the glutamic acid 170 residue as presented in Figure 3a,b. Moreover, the 4-fluorophenyl moiety is shown to be positioned towards hydrophobic alanine (Ala) 128 and alanine 129 residues while the 4-bromophenyl moiety is positioned towards the hydrophobic pockets occupied by threonine 125. On the other hand, the carbohydrazide motif of **11a** is predicted to interact with threonine 125 and glycine (Gln) 95 amino acid residues. In addition, the 4-fluorophenyl moiety is projected towards the Glycine 168 residue while the 4-bromophenyl moiety projects towards histidine 171 and glutamic acid 170 as shown in Figure 3c,d. The modelling data suggest that these scaffolds interact with critical amino acid residues in the LEDGF/p75 binding site of HIV-1 integrase.

### 2.2. Chemistry

#### 2.2.1. Synthesis of Key 1,5-Diaryl-1*H*-imidazole-4-carboxylate Esters

**Scheme 2:**
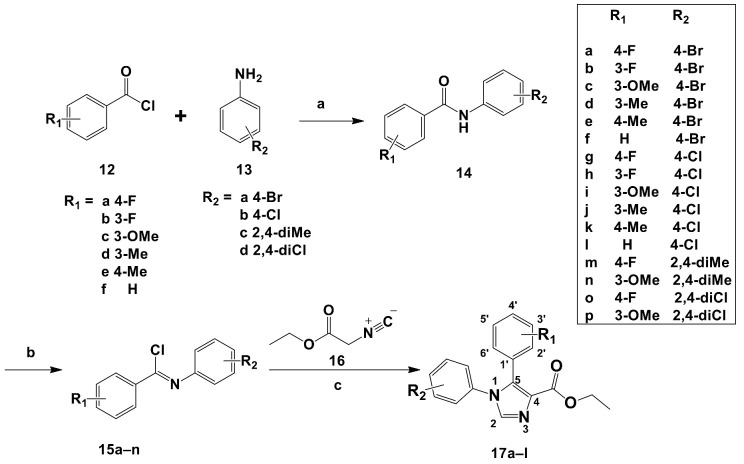
Synthesis of compounds **14**–**17**. Reaction conditions: (a) Et_3_N_,_ AcOEt, RT, 24 h; (b) SOCl_2_, reflux, 16 h; (c) ethyl isocyanoacetate, DBU, anhydrous THF,−78 °C–RT, 48 h.

Possible synthetic pathways were considered for the synthesis of the desired target 1,5-diaryl-1*H*-imidazole-4-carboxylic acids **10** and 1,5-diaryl-1*H*-imidazole-4-carbohydrazides **11**. The approach adopted was to start with reaction of an acyl chloride **12** with an aniline derivative **13** to give the corresponding amide **14** that could be readily converted into the corresponding imidoyl chloride **15** (Scheme 2). The key step in the synthesis was the cycloaddition reaction between ethyl isocyanoacetate **16** and a range of imidoyl chlorides to construct a range of key 1,5-diaryl-1*H*-imidazole-4-carboxylate ester intermediates **17**. Facile conversion of the ester intermediates into their corresponding carboxylic acid and carbohydrazide derivatives ultimately gave rise to the desired targets **10** and **11**, respectively. Judicial choice of differently substituted starting materials using this approach enabled a detailed and thorough structure-activity relationship (SAR) analysis of the desired final compounds to be undertaken. The route was initially tested starting from commercially available 4-fluorobenzoyl chloride **12a** and 4-bromoaniline **13a**. A mixture of 4-fluorobenzoyl chloride **12a** and 4-bromoaniline **13a** in ethyl acetate in the presence of Et_3_N was reacted at room temperature for 16 h to afford *N*-(4-bromophenyl)-4-fluorobenzamide **14a** in excellent yield, which was utilized directly in the next step without purification [51,52,53]. The amide **14a** was then subjected to a neat reaction by heating under reflux with excess SOCl_2_ for 8 h to give *N*-(4-bromophenyl)-4-fluorobenzimidoyl chloride **15a**. This imidoyl chloride was isolated in quantitative yield and was subsequently used without purification [51,54,55,56]. Compound **15a** was then subjected to reaction with ethyl isocyanoacetate **16**. Specifically, a mixture of stoichiometric amounts of ethyl isocyanoacetate **16**, 1,8-diazabicyclo[5.4.0]undec-7-ene (DBU) in dry THF under argon was cooled to −78 °C, followed by subsequent addition of **15a**. The reaction was allowed to warm to room temperature and stirred for 48 h, affording the desired novel ethyl 1-(4-bromophenyl)-5-(4-fluorophenyl)-1*H*-imidazole-4-carboxylate **17a** in 64% yield, after column chromatographic purification.

The optimized conditions were then applied to various commercially available benzoyl chlorides **12b**–**f** and aniline derivatives **13a**–**h** to establish the scope and limitations of the reaction. First, we studied the transformation of the amides **14b**–**p** into the essential *N*-aryl benzimidoyl chloride intermediates **15**. It was found that intermediates **15b**–**n** were successfully synthesized in quantitative yields after removal of excess SOCl_2_ and compounds were used in the subsequent reaction without purification. However, when the aniline ring had two chlorine substituents (at the *para*- and *ortho*-positions, i.e., **14o** and **14p**), no product was obtained and TLC analysis indicated the presence of unreacted starting materials. The series of *N*-aryl benzimidoyl chloride intermediates **15b–n** was then subsequently subjected to cyclization with the isocyanide **16**. After column chromatographic purification, only the ethyl 1,5-diaryl-1*H*-imidazole-4-carboxylate derivatives derived from aniline motifs substituted with the *para*-electron-withdrawing groups (i.e., **17a**–**l** in Table 1) were isolated in adequate purity. It was noted that scaffolds containing electron-donating substituents on the aryl ring directly attached to the 5-position of the imidazole motif were obtained in far poorer yield than those containing electron-withdrawing groups. For instance, the reasonable yields of 64% for **17a**, 58% for **17b**, 72% for **17g**, and 67% for **17h** were achieved when the electron-withdrawing fluorine atom occupied the *para*- or the *meta*- positions on the 5-aryl ring. On the other hand, the presence of an electron-donating group (i.e., methyl and methoxy substituents) attached to the *para*- or the *meta*- positions of the 5-aryl group led to poor reactivity and gave **17c**–**e** and **17i**–**k** in poor yields of between 18% and 41%. It was further observed that the absence of substituents on the 5-aryl ring also negatively affected the yields of the products (**17e** was obtained in 22% and **17l** in 37% yield). Unfortunately, the presence of electron-donating methyl groups at the *para*- and *ortho*- positions on the aryl ring derived from the aniline motif (i.e., substrates **15m** and **15n** in Scheme 2) was shown to hinder product formation entirely. It is not clear if the difficulty in forming the imidazole ring under the reaction conditions described can be attributed primarily to the steric hindrance of these groups or their electron-donating nature. However, electron-donating groups are expected to strongly disfavor the reaction by decreasing the electrophilicity of the imidoyl chloride carbon atom that undergoes nucleophilic attack by the α-carbon of the isocyanide. The structures of compounds **17a**–**l** are in accordance with their ^1^H and ^13^C NMR spectra (see Supplementary Materials, Figures S1–S24) and MS-ESI. The ^1^H NMR spectra of compounds **17a**–**l** are characterized by the presence of the methine proton of the imidazole ring at ca. 7.6 ppm, the methylene protons of the ethyl group at ca. 4.2 ppm, as well as the methyl protons which appear at ca. 1.2 ppm as a triplet (Figures S1–S24). The ^13^C NMR spectra of the title compounds displayed the methine-, methylene-, and methyl carbons at ca. 137, 60, and 14 ppm, respectively. To the best of our knowledge, there are no previous reports on the cycloaddition reactions of ethyl isocyanoacetate and *N*-aryl benzimidoyl chloride intermediates for the preparation of these novel ethyl 1,5-diaryl-1*H*-imidazole-4-carboxylate derivatives **17a**–**l** as described herein.

**Scheme 3:**
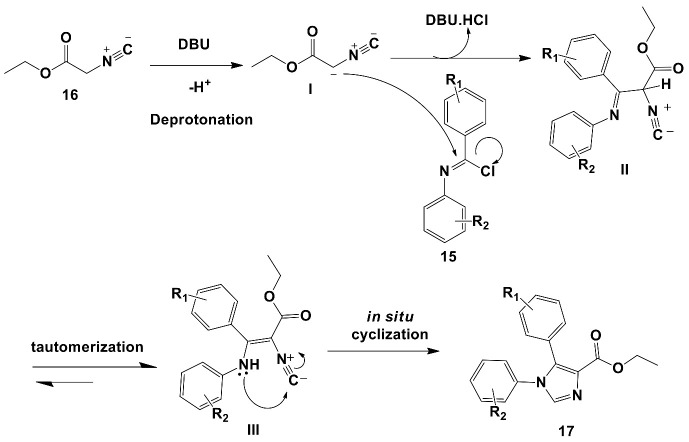
Plausible mechanism for the formation of product **17**.

A mechanistic pathway for the formation of 1,5-diaryl-1*H*-imidazole-4-carboxylate esters **17** is proposed in Scheme 3. The initial step is thought to involve activation of ethyl isocyanoacetate **16** under basic conditions to generate anion **I** [42]. The α-carbon of the intermediate **I** then undertakes a nucleophilic attack on the C=N double bond of **15** to afford intermediate **II**, with the elimination of a chloride ion as the DBU hydrochloride salt. Intermediate **II** tautomerizes to produce **III**, which then undergoes in situ cyclization and proton transfer to give the desired product **17**. 

#### 2.2.2. Synthesis of Novel 5-Diaryl-1*H*-imidazole-4-carboxylic Acids and 1,5-Diaryl-1*H*-imidazole-4-carbohydrazides

When compounds **17a**–**j** were hydrolyzed (using conditions d in Scheme 4), the resulting novel 1,5-diaryl-1*H*-imidazole-4-carboxylic acids **10a**–**l** were obtained in yields ranging from 68 to 83% (Table 2) while the reaction of **17a**,**b**, **17d**,**e**, and **17g**–**l** with hydrazine monohydrate in ethanol heated at reflux temperature (conditions e in Scheme 4) yielded the desired novel 1,5-diaryl-1*H*-imidazole-4-carbohydrazide derivatives **11a**,**b**, **11d**,**e**, **11g**–**l** in yields ranging from 58 to 76% (Scheme 4) [57]. Unfortunately, there was insufficient material available for the conversion of compounds **11c** and **11f** to the corresponding hydrazide derivatives. The complete hydrolysis to products **10a**–**l** was evidenced by the disappearance of the signals corresponding to the ethyl group in their ^1^H and ^13^C NMR spectra (Figures S25–S48) and the appearance of a broad, downfield signal at ca. 12 ppm corresponding to the acidic proton of the carboxylic acid group (COOH) in the ^1^H NMR spectra. Meanwhile, the formation of carbohydrazides **11** was evident from the appearance of signals between ca. 9.2–9.3 and 4.3–4.4 ppm corresponding to NH and NH_2_, respectively (Figures S49–S68).

### 2.3. Biological Evaluation

#### 2.3.1. HIV-1 IN-LEDGF AlphaScreen^TM^ Assays

The inhibition of LEDGF/p75-HIV IN protein-protein activity by the newly synthesized 1,5-diaryl-1*H*-imidazole-4-carboxylic acids **10a**–**l** and 1,5-diaryl-1*H*-imidazole-4-carbohydrazide derivatives **11a**,**b**, **11d**,**e**, and **11g–l** was assessed using an Amplified Luminescent Proximity Homogeneous Assay (AlphaScreen™ assay) at 100 μM compound concentration [58]. MUT 101 (**3**), a compound that inhibits both IN-LEDGF/p75 interaction and IN strand transfer activity while enhancing IN-IN interaction, was used as a positive control [21]. Experimental protocols were conducted in triplicate, and the percentage inhibition results are shown in Table 3. Compounds showing percentage inhibition rates of higher than 50% were considered to be active. The biological evaluation data revealed a total of seventeen potential inhibitors (out of twenty-two tested compounds) that exceeded our pre-defined 50% inhibitory threshold of disrupting the HIV-1 IN-LEDGF/p75 interaction. Carbohydrazide **11e**, with the *para*-methylphenyl group at the 5-position and the *para*-bromophenyl group at the *N*-site of the imidazole moiety exhibited the highest inhibition of 89%, which is close to the value obtained for reference compound MUT 101, with 90% inhibition. Carboxylic acid **10k**, with the *para*-methylphenyl ring at the 5-position and the *para*-chlorophenyl unit at the *N-*site of the imidazole motif showed the second-highest level of inhibition, at 85%. This was followed closely by carboxylic acid **10d**, with the *meta*-methylphenyl and the *para*-bromophenyl motifs at the 5- and *N*- sites, respectively, which showed 84% inhibition for disruption of the LEDGF/p75-IN interaction. Interestingly, these top 3 inhibitors all have a methyl substituent attached to the aryl ring at the 5-position of the imidazole motif, which is also present in the majority of known LEDGF/p75 interaction inhibitors. This indicates that the presence of the methyl substituent on the structure could be essential to the potency of the compound. The carboxylic acids, **10a** (with the *para*-fluorophenyl and the *para*-bromophenyl motifs attached at the 5- and *N*-positions, respectively) and **10e** (with the *para*-methylphenyl and the *para*-bromophenyl units at the 5- and *N*-site) displayed the fourth and fifth best inhibition of 83% and 82%, respectively. Compounds **10b**, **10i**, **10j**, **11d**, and **11k** exhibited good overall inhibition, with percentage inhibitions ranging from 67% to 78% while chemical entities **10c**, **10h**, **11a**, **11b**, **11h**, and **11i** disrupted the HIV-1 IN and LEDGF/p75 interaction by 50–59%, demonstrating moderate inhibitory activity. Marginal percentage inhibition values of 17, 35, and 33% were observed for compounds **10f**, **10l**, and **11j**, respectively, while compounds **11g** and **11i** were the only inactive molecules. According to the data, with the notable exception of **11g** and **11i**, the results did support the initial hypothesis that incorporation of carboxylic acid and carbohydrazide moieties onto the structure of hit **9** would have a significant impact on the binding capabilities of the compounds.

#### 2.3.2. Direct ELISA Assay

**Figure 4:**
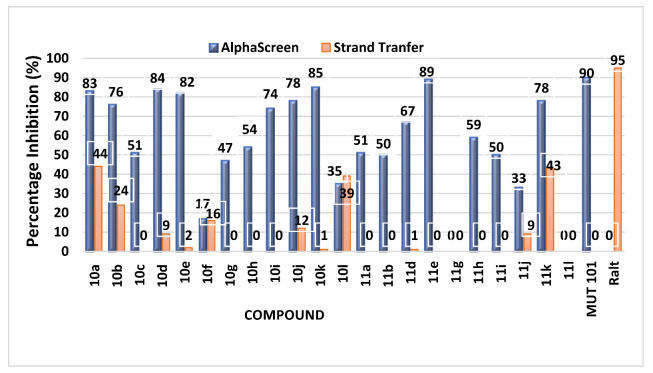
A comparison of carboxylic acid (**10**) and carbohydrazide (**11**) inhibition percentages in AlphaScreen^TM^ (blue) and ELISA (orange) assays at 100 μM.

The new compounds **10a**–**l**, **11a**,**b**, **11d**,**e**, and **11g–l** were also examined for their ability to bind to divalent cations in the catalytic core of integrase using ELISA assays at 100 μM concentration [59]. The assay was carried out in order to determine if the newly synthesized compounds act similarly to MUT 101 (**3**), which inhibits both IN-LEDGF interaction and IN strand transfer. A control compound, raltegravir, known to bind divalent metals within HIV-1 IN, was also tested alongside the test compounds. The experimental protocols were carried out in duplicate, and their relative percentage inhibitory values are shown in Table 3 and Figure 4. The data showed that most of the compounds showed no significant inhibition of strand transfer. Only three compounds, viz. **10a**, **10l**, and **11k**, showed marginal percentage inhibition of 44%, 39%, and 43%, respectively, at 100 μM compared to a 95% inhibition for raltegravir at 10 μM concentration as shown in Table 3. Thus, these results demonstrate that these two classes of compounds (**10** and **11**) may be specifically binding to the LEDGF/p75 binding site in HIV-1 integrase (Figure 4).

#### 2.3.3. Cytotoxicity and Antiviral Assays

The newly synthesized compounds **10a**–**l** and **11a**,**b**, **11d**,**e**, and **11g–l** were further examined for cytotoxicity and antiviral activity using methods similar to those previously described [30]. The assays were performed in the metallothionein type 4 (MT-4) cells, a human cell line that is highly susceptible to HIV infection. Auranofin, a compound known for its toxicity to mammalian cells, was screened alongside the compounds. Cytotoxicity screening indicates the tolerance of the system to the compounds, as well as indicating the relative toxicity of these compounds to live cells [60]. Cytotoxicity results are represented in Table 4. The data show that thirteen compounds are marginally toxic or non-toxic, having CC_50_ values of greater than or equal to 100 µM, while nine compounds exhibited slightly higher toxicity, with CC_50_ values of less than 100 µM. However, these scaffolds were not found to be overly toxic in comparison to the control, auranofin, which exhibited a CC_50_ value of 1.6 µM. The CC_50_ value of control compounds was comparable to the previously described CC_50_ values of 4.5 µM against human HepG-2 human liver cells [61]. The results indicate that most of the compounds were tolerated well by the cells.

Finally, the compounds were evaluated for their capability to inhibit the viral outgrowth of the HIV-1 subtype C replicating in a cell-based antiviral assay at 100 µM single dose concentration [62]. The antiviral percentage inhibition values of the evaluated compounds were calculated and are recorded in Table 4. The antiviral percentage activities were found to range from low to moderate, with none found to be higher than 50% at 100 µM compound concentration. Although compound **11h** was the best performer with an antiviral percentage of 40%, its potency level was possibly induced by its toxicity of 50.4 µM (Table 4). Taken together, compounds **11a**, **11b**, and **11g** with CC_50_ values of >200 µM, 158.4 µM, and >200 µM, and antiviral percentage activities of 35%, 36%, and 33%, respectively, were considered to be promising scaffolds. This indicates that the selectivity of inhibition over toxicity for these compounds was favorable. Furthermore, it was interesting to observe that the compounds with marginal antiviral activities belong to the carbohydrazide family of compounds. Additionally, although, the antiviral percentage activities were minimal in comparison to raltegravir (tested at 10 µM), the results indicate that the inclusion of the carbohydrazide motif positively influenced the binding more than the carboxylic acid moiety.

### 2.4. Multimerization Gel Assay as a Secondary Screening Assay

**Figure 5:**
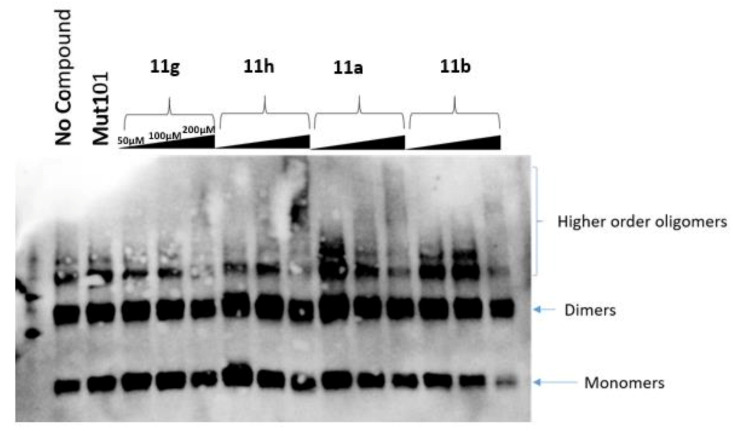
Western blot illustration of the multimerization caused by tested compounds. Compounds **11a**, **11b**, **11g**, and **11h** were selected for multimerization studies. Compounds were tested at 50 µM, 100 µM, and 200 µM. MUT101 was included as a control and tested at 50 µM only. Between the no-compound control and MUT101, an increase of higher order IN forms is noticed. An increase in higher order IN oligomers is most pronounced in compounds **11a**, **11b**, and **11h** and the equilibrium shifts to higher order forms as a factor of compound concentration.

Chemical covalent crosslinking of IN has been shown to stabilize the multimer forms when observed on SDS-PAGE [63]. This is particularly useful due to the disruption of these multimers due to SDS and reducing conditions in the gel. The IN cross-linking assay was performed as described previously [63]. This allowed for the capture of multimer forms and provided useful information in determining the effects that the compounds had on IN multimerization. Four compounds **11a**, **11b**, **11g**, and **11h**, exhibiting marginal antiviral percentage inhibitory activities were selected for the multimerization studies in a Western blot gel multimerization assay at concentrations of 50, 100, and 200 µM. Compounds **11a**, **11b**, and **11h** appear to cause an increase in higher-order oligomeric forms of IN as shown in Figure 5. There is a clear shift in the oligomeric equilibrium as a result of increasing concentrations of the compounds. These results indicate that the tested compounds cause multimerization, similar to previously described allosteric integrase inhibitors [64,65].

## 3. Experimental Protocols

### 3.1. Chemistry

#### 3.1.1. Materials and Methods

All commercially available reagents were purchased from Sigma Aldrich (Schnelldorf, Germany) and used without further purification. Dry solvents were used from an LC-Tech SP-1 Solvent Purification System (LC Technology Solutions Inc., Seabrook, New Hampshire, USA) and kept under argon. TLC was performed on pre-coated Merck silica gel F254 plates and viewed under UV light (254 nm) or following exposure to iodine vapor. Column chromatography was performed using Merck Silica gel 60 [particle size 0.040–0.063 mm (230–400 mesh)]. NMR spectra were recorded on a 400 MHz Bruker Avance Spectrometer (Bruker BioSpin, Rheinstetten, Germany) at 298 K equipped with a BBI 5 mm probe and solvent calibrated. High-resolution mass spectra were recorded on an LC-MS system consisting of a Dionex Ultimate 3000 Rapid Separation LC system equipped with a C-18 pre-coated column and coupled to a MicrOTOF QII Bruker mass spectrometer (Bruker Daltonik GmbH, Bremen, Germany) fitted with an electrospray source and operating in positive mode. Sodium formate clusters were used for internal calibration. The chemical shifts (δ) are expressed in parts per million, according to the solvent residual peak resonances and calibrated using solvent signals (7.26 (CDCl_3_) and 2.50 (DMSO-*d*_6_) for ^1^H NMR; 77.0 (CDCl_3_) and 39.52 (DMSO-*d*_6_) for ^13^C NMR]. LC-MS grade acetonitrile and methanol were from Sigma Aldrich and water from a Milli-Q integral system. The MicrOTOF QII source parameters source were as follows: ESI source type; auto MSMS mode; mass range: 50–3000 Da; on positive polarity; endplate offset −500 V; capillary voltage 4500 V; nebulizer N_2_ 1.2 bar; dry heater 200 °C; drying N_2_ flow 8.0 L/min. The mass analyses were performed on Data Analysis software (Bruker Daltonics, Bremen, Germany). All melting points were obtained using a Stuart SMP10 melting point apparatus and are uncorrected.

#### 3.1.2. General Procedure for the Synthesis of *N*-Aryl-benzamides **14a**–**p**

To a stirred solution containing aniline (14.4 mmol) and Et_3_N (25.1 mmol) in EtOAc (30 mL) was added benzoyl chloride (15.1 mmol) at 0 °C. After addition, the resulting reaction mixture was allowed to warm to room temperature and stirred for 16 h (monitored by TLC). Upon completion, EtOAc (20 mL) was added, followed by washing with 1M HCl, saturated NaHCO_3_ and water. The organic layer was dried over anhydrous MgSO_4_, and the solvent evaporated and dried in vacuo to afford pure *N*-aryl-benzamide derivatives [53].

#### 3.1.3. General Procedure for the Synthesis of *N*-Aryl-benzimidoyl Chloride Intermediates **15a**–**n**

A mixture of *N*-aryl-benzamide (1.0 molar equivalent) and thionyl chloride (6.0 molar equivalents) was heated to reflux until reaction completion (8 h). Excess thionyl chloride was removed by co-evaporation with toluene to afford, after drying under high vacuum, diarylimidoyl chloride and were directly used in the next step without further purification [54,55,56].

##### *N*-(4-Bromophenyl)-4-fluorobenzimidoyl Chloride **15a**

Yield: 2.01 g, 96% as a pale yellow solid; ^1^H NMR (400 MHz, CDCl_3_) δ 8.15–8.19 (2H, m, ArH), 7.52 (2H, d, *J* = 7.6 Hz, ArH), 7.14 (2H, d, *J* = 7.6 Hz, ArH), 6.89 (2H, d, *J* = 7.6 Hz, ArH); ^13^C NMR (101 MHz, CDCl_3_) δ 165.2 (d, ^1^*J*_CF_ = 252 Hz, 4-FC), 146.3, 142.7 (ArC*C*Cl), 131.9 (ArC), 131.7 (d, ^3^*J*_CF_ = 9 Hz, 2′-C and 6′-C), 131.3 (d, ^4^*J*_CF_ = 3 Hz, 1′-C), 122.3, 118.4 (ArC), 115.6 (d, ^2^*J*_CF_ = 22 Hz, 3′-C and 5′-C).

##### *N*-(4-Bromophenyl)-3-fluorobenzimidoyl Chloride **15b**

Yield: 1.84 g, 95% as pale yellow solid, ^1^H NMR (400 MHz, CDCl_3_) δ 7.94 (1H, *J* = 7.6 Hz, ArH), 7.85 (1H, *J* = 9.6 Hz, ArH), 7.52 (2H, *J* = 8.4 Hz, ArH), 7.42 (1H, dd, *J* = 7.2, 7.6 Hz, ArH), 7.24 (1H, t, *J* = 8.0 Hz, ArH), 6.90 (2H, *J* = 8.4 Hz, ArH); ^13^C NMR (101 MHz, CDCl_3_) δ 162.6 (d, ^1^*J*_CF_ = 247 Hz, 3′-CF), 146.1 (ArC*C*Cl), 142.2 (d, ^4^*J*_CF_ = 3 Hz, 6′-C), 137.2 (d, ^3^*J*_CF_ = 8 Hz, 1′-C), 132.0, 130.5, 129.93 (ArC), 125.1 (d, ^3^*J*_CF_ = 3 Hz, 5′-C), 122.3 (ArC), 119.2 (d, ^3^*J*_CF_ = 22 Hz, 2′-C), 116.3 (d, ^3^*J*_CF_ = 24 Hz, 4′-C).

##### *N*-(4-Bromophenyl)-3-methoxybenzimidoyl Chloride **15c**

Yield: 3.24 g, 98% as pale yellow solid; ^1^H NMR (400 MHz, CDCl_3_) δ 7.72 (1H, d, *J* = 7.6 Hz, ArH), 7.66 (1H, s, ArH), 7.49 (2H, d, *J* = 8.8 Hz, ArH), 7.33 (1H, t, *J* = 8.0 Hz, ArH), 7.07 (1H, d, *J* = 8 Hz, ArH), 6.87 (2H, d, *J* = 7.6 Hz, ArH), 3.86 (3H, s, ArOCH_3_); ^13^C NMR (101 MHz, CDCl_3_) δ 159.6 (Ar*C*OCH_3_), 146.5, 143.9 (ArCCl), 136.5, 131.9, 129.4, 122.2, 122.1, 118.7, 118.3 (ArCBr), 114.0 (ArC), 55.5 (ArCO*C*H_3_).

##### *N*-(4-Bromophenyl)-3-methylbenzimidoyl Chloride **15d**

Yield: 2.68 g, 98% as pale yellow solid: ^1^H NMR (400 MHz, CDCl_3_) δ 7.97–8.03 (2H, m, ArH) 7.56 (2H, d, *J* = 8.4 Hz, ArH), 7.41 (2H, d*, J* = 4.0 Hz, ArH), 6.943 (2H, d, *J* = 8.4 Hz, ArH), 2.24 (3H, s, ArCH_3_); ^13^C NMR (101 MHz, CDCl_3_) δ 145.9, 144.2 (ArCCl), 138.2, 135.0, 130.4, 129.8, 128.9, 128.3, 126.7, 121.9 (ArCBr), 24.1 (ArC*C*H_3_).

##### *N*-(4-Bromophenyl)-4-methylbenzimidoyl Chloride **15e**

Yield: 2.74 g, 100% as pale yellow solid; ^1^H NMR (400 MHz, CDCl_3_) δ 7.98 (2H, d, *J* = 8.4 Hz, ArH), 7.45 (2H, d, *J* = 8.4Hz, ArH), 7.21 (2H, d, *J* = 8.0Hz), 6.83 (2H, d, *J* = 8.4 Hz, ArH), 2.38 (3H, s, ArCH_3_); ^13^C NMR (101 MHz, CDCl_3_) δ 146.7, 144.2, 143.1 (ArCCl), 132.0, 131.6, 129.5, 129.3, 122.5, 118.2 (ArC), 21.5 (ArC*C*H_3_).

##### *N*-(4-Bromophenyl)benzimidoyl Chloride **15f**

Yield: 2.56 g, 96% as pale yellow solid: pale yellow solid; ^1^H NMR (400 MHz, CDCl_3_) δ 8.16 (2H, d, *J* = 7.6 Hz, ArH), 7.47–7.59 (5H, m, ArH), 6.91 (2H, d, *J* = 8.8 Hz, ArH); ^13^C NMR (101 MHz, CDCl_3_) δ 146.5, 144.0 (ArCCl), 135.2, 132.2, 132.0, 131.9, 129.4, 128.5, 122.3, 118.3 (ArC).

##### *N*-(4-Chlorophenyl)-4-fluorobenzimidoyl Chloride **15g**

Yield: 3.00 g, 96% as pale yellow solid; ^1^H NMR (400 MHz, CDCl_3_) δ = 8.11–8.14 (2H, m, ArH), 7.33 (2H, d, *J* = 8.8 Hz, ArH); 7.09–7.13 (3H, m, ArH); 7.12 (1H, t, *J* = 7.8Hz, ArH), 6.85 (2H, d, *J* = 8.4 Hz, ArH); ^13^C NMR (101 MHz, CDCl_3_) δ = 165.2 (^1^*J*_CF_ = 248 Hz, 4′-FC), 145.7, 142.7 (ArC*C*Cl), 131.6 (*^3^J*_CF_ = 9 Hz, 2′-C and 6′-C), 131.3 (^4^*J*_CF_ = 3 Hz, C-1′), 130.6, 129.0, 122.0, 115.4 (d, ^3^*J*_CF_ = 22 Hz, 3′-C and 5′-C).

##### *N*-(4-Chlorophenyl)-3-fluorobenzimidoyl Chloride **15h**

Yield: 3.06 g, 98% as pale yellow solid; ^1^H NMR (400 MHz, CDCl_3_) δ 8.83 (2H, *J* = 7.6 Hz, ArH), 7.74 (1H, d, *J* = 10.0 Hz ArH), 7.27–7.36 (3H, m, ArH), 6.85 (2H, *J* = 8.4 Hz, ArH); ^13^C NMR (101 MHz, CDCl_3_) δ 162.5 (d, ^1^*J*_CF_ = 247 Hz, 3′-CF), 146.1, 142.5 (d, ^4^*J*_CF_ = 3.0 Hz, 6′-C), 137.3 (d, ^3^*J*_CF_ = 8 Hz, 1′-C), 132.0, 130.0, 129.3 (ArC), 125.1 (d, ^3^*J*_CF_ = 3.0 Hz, 5′-C), 122.1 (ArC), 119.1 (d, ^3^*J*_CF_ = 22 Hz, 2′-C), 116.2 (d, ^3^*J*_CF_ = 24 Hz, 4′-C).

##### *N*-(4-Chlorophenyl)-3-methoxybenzimidoyl Chloride **15i**

Yield: 3.14 g, 97% as pale yellow solid; ^1^H NMR (400 MHz, CDCl_3_) δ 7.70 (2H, d, *J* = 7.6 Hz, ArH), 7.66 (1H, s, ArH), 7.32–7.34 (3H, m, ArH), 7.05 (1H, d, *J* = 8.4 Hz, ArH), 6.92 (2H, d, *J* = 7.6 Hz, ArH), 3.82 (3H, s, ArOCH_3_); ^13^C NMR (101 MHz, CDCl_3_) δ 159.5 (Ar*C*OCH_3_), 145.9, 143.8 (ArCCl), 136.4, 130.4. 129.3, 128.9, 122.0, 121.8, 118.5, 113.9 (ArC), 55.5 (ArCO*C*H_3_).

##### *N*-(4-Chlorophenyl)-3-methylbenzimidoyl Chloride **15j**

Yield: 3.02 g, 98% as pale yellow solid; ^1^H NMR (400 MHz, CDCl_3_): δ = 8.05 (2H, t, *J* = 6.0 Hz, ArH), 7.48–7.79 (4H, m, ArH), 7.94 (2H, d, *J* = 8.4 Hz, ArH); 2.52 (3H, s, ArCH_3_) [54].

##### *N*-(4-Chlorophenyl)-4-methylbenzimidoyl Chloride **15k**

Yield: 2.93g, 95% as pale yellow solid; ^1^H NMR (400 MHz, CDCl_3_): δ = 8.00 (2H, d, *J* = 7.6 Hz, ArH), 7.30 (2H, d, *J* = 8.4 Hz, ArH), 7.21 (2H, d, *J* = 8.0, ArH); 6.91 (2H, d, *J* = 8.0 Hz,); 2.39 (3H, s, ArCH_3_); ^13^C NMR (101 MHz, CDCl_3_) δ 146.0, 144.0 (ArCCl), 142.9, 132.4, 130.3, 129.4, 128.9, 122.0 (ArC), 21.4 (ArC*C*H_3_).

##### *N*-(4-Chlorophenyl)benzimidoyl Chloride **15l**

Yield: 2.68 g, 98% as pale yellow solid; ^1^H NMR (400 MHz, CDCl_3_) δ 8.19 (2H, d, *J* = 7.6 Hz, ArH), 7.39–7.60 (5H, m, ArH), 6.99 (2H, d, *J* = 8.4 Hz, ArH); ^13^C NMR (101 MHz, CDCl_3_) δ 145.9, 144.0 (ArCCl), 135.2, 132.1, 130.5, 129.4, 128.9, 128.4, 121.9 (ArC).

##### *N*-(2.4-Dimethylphenyl)-4-fluorobenzimidoyl Chloride **15m**

Yield: 2.95 g, 100% as pale yellow solid; ^1^H NMR (400 MHz, CDCl_3_) δ 8.21–8.24 (2H, m, ArH), 7.10–7.20 (5H, m, ArH), 6.8 (1H, d, *J* = 8.2 Hz, ArH), 2.37 and 2.20 (2 × ArCCH_3_); ^13^C NMR (101 MHz, CDCl_3_) δ 165.2 (d, ^1^*J*_CF_ = 253 Hz, 4-FC), 143.9, 141.6 (ArC*C*Cl), 134.7, 131.7 (d, ^3^*J*_CF_ = 9 Hz, 2′-C and 6′-C), 131.1, 128.4, 126.8, 119.2 9 (ArC), 115.9 (d, ^3^*J*_CF_ = 22 Hz, C-3′ and C-6′), 20.9 and 17.8 (2 × ArC*C*H_3_).

##### *N*-(2.4-Dimethylphenyl)-3-methoxybenzimidoyl Chloride **15n**

Yield: 2.68 g, 98% as pale yellow solid; ^1^H NMR (400 MHz, CDCl_3_) δ 7.80 (1H, d, *J* = 8.2 Hz, ArH), 7.76 (1H, t, *J* = 5.6 Hz, ArH), 7.39 (1H, t, *J* = 5.6 Hz, ArH), 7.07–7.14 (3H, m, ArH), 6.82 (1H, d, *J* = 7.6 Hz, ArH), 3.91 (3H, s, ArOCH_3_), 2.37 and 2.20 (6H, 2 × s, 2 × ArCH_3_); ^13^C NMR (101 MHz, CDCl_3_) δ 159.6 (Ar*C*OCH_3_), 143.9 (ArCCl), 143.0, 136.6, 134.6, 131.0, 129.3, 128.3, 126.8, 122.0, 119.2, 118.3, 114.0 (ArC), 55.4 (ArCO*C*H_3_), 20.9 and 17.7 (2 × ArC*C*H_3_).

#### 3.1.4. General Procedure for the Synthesis of Ethyl 1,5-Diaryl-1*H*-imidazoyl Carboxylates **17a–l**

A mixture of ethyl isocyanoacetate **16** (5.1 mmol) and DBU (5.1 mmol) in anhydrous THF (25 mL) was cooled to −78 °C under argon. N-aryl-benzimidoyl chloride **15** (5.1 mmol) was then added under a flow of argon and the resulting reaction mixture was allowed to warm to room temperature. After 48 h, the reaction was quenched with saturated sodium bicarbonate and extracted with EtOAc, washed with brine, dried over anhydrous MgSO_4_, and concentrated in vacuo. The crude product was purified by column chromatography, (eluting with EtOAc/ Hexane (1:4), followed by 100% EtOAc) to afford the desired ethyl 1,5-diaryl-1*H*-imidazole carboxylate **17**.

##### Ethyl 1-(4-Bromophenyl)-5-(4-fluorophenyl)-1*H*-imidazole-4-carboxylate **17a**

Yield: 1.27 g, 64% as a pale yellow solid; mp: 145–147 °C; ^1^H NMR (400 MHz, CDCl_3_): δ = 7.69 (1H, s, 2-CH), 7.44 (2H, d, *J* = 8.8 Hz, ArH), 7.17 (2H, m, ArH), 6.92–6.99 (4H, m, ArH), 4.23 (2H, q, *J* = 7.2 Hz, OCH_2_CH_3_), 1.24 (3H, t, *J* = 7.2 Hz, OCH_2_CH_3_); ^13^C NMR (101 MHz, CDCl_3_): δ = 162.9 (d, ^1^*J_CF_* = 251 Hz, FC-4′), 162.7 (C=O), 137.4 (C-2), 136.9, 134.4, 132.7 (d, ^3^*J_CF_* = 10 Hz, C-2′ and C-6′), 130.9, 127.3, 123.8 (d, ^4^*J_CF_* = 3 Hz, C-1′), 122.8 (ArCBr), 115.2 (d, ^3^*J_CF_* = 22 Hz, C-3′ and C-5′), 60.6 (OCH_2_CH_3_), 14.2 (OCH_2_CH_3_); HRMS (ESI-TOF) *m*/*z*: [M + H]^+^ Calcd. for C_18_H_15_^79^BrFN_2_O_2_ 389.0295, found 389.0301.

##### Ethyl 1-(4-Bromophenyl)-5-(3-fluorophenyl)-1*H*-imidazole-4-carboxylate **17b**

Yield: 1.15 g, 58% as a pale yellow solid; mp: 129–131 °C; ^1^H NMR (400 MHz, CDCl_3_) δ 7.74 (1H, s, 2-CH), 7.48 (2H, d, *J* = 8 Hz, ArH), 7.28–7.31 (1H, m, ArH), 6.98–7.08 (5H, m, ArH), 4.28 (2H, q, *J* = 7 Hz, OCH_2_CH_3_), 1.28 (3H, t, *J* = 7 Hz, OCH_2_CH_3_); ^13^C NMR (101 MHz, CDCl_3_) δ 162.4 (C=O), 161.9 (d, ^1^*J_CF_* = 247 Hz, FC-3′), 137.5 (C-2), 136.3 (d, ^4^*J_C_*_F_ = 2 Hz, C-6′), 134.7, 132.6, 131.1, 129.8 (d, ^3^*J_CF_* = 9 Hz, C-5′), 129.6, 129.5, 127.7 (d, ^3^*J_CF_* = 3 Hz, C-1′), 122.7 (ArC), 117.7 (d, ^3^*J_CF_* = 23 Hz, C-4′), 115.8 (d, ^3^*J_CF_* = 22 Hz, C-2′), 60.5 (OCH_2_CH_3_), 14.1 (OCH_2_CH_3_); HRMS (ESI-TOF) *m*/*z*: [M + H]^+^ Calcd. for C_18_H_15_^79^BrFN_2_O_2_ 389.0295, found 389.0301.

##### Ethyl 1-(4-Bromophenyl)-5-(3-methoxyphenyl)-1*H*-imidazole-4-carboxylate **17c**

Yield: 368 mg, 18% as a yellow solid; ^1^H NMR (400 MHz, CDCl_3_) δ 7.70 (1H, s, 2-CH), 7.28 (2H, d, *J* = 8.4 Hz, ArH), 7.18 (1H, t, *J* = 8.0 Hz, ArH), 7.01 (2H, d, *J* = 8.4 Hz, ArH), 6.77–6.87 (3H, m, ArH), 4.25 (2H, q, *J* = 7.2 Hz, OCH_2_CH_3_), 3.70 (3H, s, ArOCH_3_), 1.25 (3H, t, J = 7.0 Hz, OCH_2_CH_3_); ^13^C NMR (101 MHz, CDCl_3_) δ 162.6 (C=O), 159.0 (ArCOCH_3_), 137.6 (C-2), 137.2, 134.7, 132.6, 130.9, 129.1, 127.1, 123.1, 122.5, 119.3, 116.5, 114.5 (ArC), 60.5 (OCH_2_CH_3_), 55.1 (ArCOCH_3_), 14.1 (OCH_2_CH_3_); HRMS (ESI-TOF) *m*/*z*: [M + H]^+^ Calcd. for C_19_H_18_^79^BrN_2_O_3_ 401.0495, found 401.0487.

##### Ethyl 1-(4-Bromophenyl)-5-(3-methylphenyl)-1*H*-imidazole-4-carboxylate **17d**

Yield: 591 mg, 30% as a white solid; mp: 185–187 °C; ^1^H NMR (400 MHz, CDCl_3_) δ 7.69 (1H, s, 2-CH), 7.43 (2H, d, *J* = 8.4 Hz, ArH), 7.13–7.19 (2H, m, ArH), 7.05 (1H, s, ArH), 6.93–6.98 (3H, m, ArH), 4.25 (2H, q, *J* = 7.2 Hz, OCH_2_CH_3_), 2.27 (3H, s, ArCH_3_), 1.24 (3H, t, *J* = 7 Hz, OCH_2_CH_3_); ^13^C NMR (101 MHz, CDCl_3_) δ 162.6 (C=O), 138.0, 137.6 (C-2), 137.2, 134.6, 132.5, 131.3, 130.7, 129.7, 128.0, 127.8, 127.7, 127.1, 122.4 (ArC), 60.4 (OCH_2_CH_3_), 21.3 (ArCCH_3_), 14.1 (OCH_2_CH_3_); HRMS (ESI-TOF) *m*/*z*: [M + H]^+^ Calcd. for C_19_H_18_^79^BrN_2_O_2_ 385.0546, found 385.0557.

##### Ethyl 1-(4-Bromophenyl)-5-(4-methylphenyl)-1*H*-imidazole-4-carboxylate **17e**

Yield: 690 mg, 35% as a yellow solid; mp: 142–144 °C; ^1^H NMR (400 MHz, CDCl_3_) δ 7.69 (1H, s, 2-CH), 7.28 (2H, d, *J* = 9.0 Hz, ArH), 7.10 (4H, m, ArH), 6.99 (2H, d, *J* = 8.0 Hz, ArH), 4.26 (2H, q, *J* = 7.0 Hz, OCH_2_CH_3_), 2.32 (3H, s, ArCH_3_), 1.27 (3H, t, *J* = 7.0 Hz, OCH_2_CH_3_); ^13^C NMR (101 MHz, CDCl_3_) δ 162.7 (C=O), 139.5, 138.1 (ArC), 137.6 (C-2), 134.7, 132.0, 131.4, 130.8, 129.8, 127.8, 127.1, 122.4 (ArC), 60.4 (OCH_2_CH_3_), 21.3 (ArCCH3), 14.2 (OCH_2_CH_3_); HRMS (ESI-TOF) *m*/*z*: [M + H]^+^ Calcd. for C_18_H_18_^79^BrFN_2_O_2_ calc. 385.0546, found. 385.0548.

##### Ethyl 1-(4-Bromophenyl)-5-phenyl-1*H*-imidazole-4-carboxylate **17f**

Yield: 417 mg, 22% as a white solid; mp: 138–140 °C; ^1^H NMR (400 MHz, CDCl_3_) δ 7.70 (1H, s, 2-CH), 7.46 (2H, d, *J* = 8.4 Hz, ArH), 7.28–7.33 (3H, m, ArH), 7.21–7.23 (2H, d, *J* = 6.8 Hz, ArH), 6.93 (2H, d, J = 8.4 Hz, ArH), 4.26 (2H, q, *J* = 7.2 Hz, OCH_2_CH_3_), 1.25 (3H, t, *J* = 7.2 Hz, OCH_2_CH_3_); ^13^C NMR (101 MHz, CDCl_3_) δ 162.7 (C=O), 137.9 (C-2), 137.3, 134.6, 132.6, 130.8, 130.7, 129.0, 128.0, 127.9, 127.2, 122.5 (ArC), 60.5 (OCH_2_CH_3_), 14.2 (OCH_2_CH_3_); HRMS (ESI-TOF) *m*/*z*: [M + H]^+^ Calcd. for C_16_H_16_^79^BrN_2_O_2_ 371.0390, found 371.0398.

##### Ethyl 1-(4-Chlorophenyl)-5-(4-fluorophenyl)-1*H*-imidazole-4-carboxylate **17g**

Yield: 1.28 g, 72% as a yellow solid; mp: 146–148 °C; ^1^H NMR (400 MHz, CDCl_3_) δ 7.69 (1H, s, 2-CH), 7.29 (2H, d, *J* = 8.0 Hz, ArH), 7.18 (2H, *J* = 7.0 Hz, ArH), 6.96–7.00 (4H, m, ArH), 4.25 (2H, q, *J* = 7.0 Hz, OCH_2_CH_3_), 1.26 (3H, t, *J* = 7.0 Hz, OCH_2_CH_3_); ^13^C NMR (101 MHz, CDCl_3_) δ 162.8 (d, ^1^*J_CF_* = 251 Hz, FC-4′), 162.6 (C=O), 137.4 (C-2), 136.9, 134.7, 133.8 (ArC), 132.7 (d, ^3^*J_CF_* = 9 Hz, C-2′ and C-6′), 130.8, 129.7, 126.9 (ArC), 123.8 (C-1′), 115.2 (d, ^3^*J_CF_* = 24 Hz, C-3′ and C-5′), 60.5 (OCH_2_CH_3_), 14.1 (OCH_2_CH_3_); HRMS (ESI-TOF) *m*/*z*: [M + H]^+^ Calcd. for C_18_H_15_ClFN_2_O_2_ 345.0801, found: 345.0810.

##### Ethyl 1-(4-Chlorophenyl)-5-(3-fluorophenyl)-1*H*-imidazole-4-carboxylate **17h**

Yield: 1.19 g, 67% as a pale yellow solid; mp: 118–120 °C; ^1^H NMR (400 MHz, CDCl_3_) δ 7.74 (1H, s, 2-CH), 7.33 (2H, d, *J* = 8.0 Hz, ArH), 7.28–7.32 (1H, m, ArH), 6.99–7.07 (5H, m, ArH), 4.28 (2H, q, *J* = 7.0 Hz, OCH_2_CH_3_), 1.28 (3H, t, *J* = 7.0 Hz, OCH_2_CH_3_); ^13^C NMR (101 MHz, CDCl_3_) δ 162.4 (C=O), 161.9 (d, ^1^*J_CF_* = 247 Hz, FC-3′), 137.5 (C-2), 136.3 (d, ^4^*J_CF_* = 2 Hz, C-6′), 134.7, 133.6, 131.0, 129.9, 29.8 (ArC), 129.4 (d, ^3^J_CF_ = 9 Hz, C-5′), 126.8, (ArC), 126.4 (d, ^3^*J_CF_* = 3Hz, C-1′), 117.9 (d, ^3^*J_CF_* = 22 Hz, C-4′), 116.0 (d, ^3^*J_CF_* = 21 Hz, C-2′), 60.5 (OCH_2_CH_3_), 14.1 (OCH_2_CH_3_); HRMS (ESI-TOF) *m*/*z*: [M + H]^+^ Calcd. for C_18_H_15_ClFN_2_O_2_ 345.0801, found: 345.0804.

##### Ethyl 1-(4-Chlorophenyl)-5-(3-methoxyphenyl)-1*H*-imidazole-4-carboxylate **17i**

Yield: 582 mg, 32% as a yellow solid; mp: 127–129 °C; ^1^H NMR (400 MHz, CDCl_3_) δ 7.69 (1H, s, 2-CH), 7.28 (2H, d, *J* = 9.0 Hz, ArH), 7.17 (1H, t, *J* = 6.0 Hz, ArH), 7.01 (2H, d, *J* = 9.0 Hz, ArH), 6.77–6.87 (3H, m, ArH), 4.25 (2H, q, *J* = 7.2 Hz, OCH_2_CH_3_), 3.69 (3H, s, ArOCH_3_), 1.25 (3H, t, *J* = 7.0 Hz, OCH_2_CH_3_); ^13^C NMR (101 MHz, CDCl_3_) δ 162.6 (C=O), 158.9 (ArCOCH_3_), 137.7 (C-2), 137.3, 134.5, 134.1, 130.8, 129.9, 129.6, 129.0, 126.8, 123.1, 116.5, 114.5 (ArC), 60.5 (OCH_2_CH_3_), 55.1 (ArCOCH_3_), 14.2 (OCH_2_CH_3_); HRMS (ESI-TOF) *m*/*z*: [M + H]^+^ Calcd. for C_19_H_18_ClN_2_O_3_ 357.1000, found: 357.1007.

##### Ethyl 1-(4-Chlorophenyl)-5-(3-methylphenyl)-1*H*-imidazole-4-carboxylate **17j**

Yield: 713 mg, 41% as a yellow solid; mp: 144–146 °C; ^1^H NMR (400 MHz, CDCl_3_) δ = 7.69 (1H, s, 2-CH), 7.28 (2H, d, *J* = 9.0 Hz, ArH), 7.12–7.22 (2H, m, ArH), 7.05–7.17 (4H, m, ArH), 4.25 (2H, q, *J* = 7.0 Hz, OCH_2_CH_3_), 2.27 (3H, s, ArCH_3_), 1.25 (3H, t, *J* = 7.0 Hz, OCH_2_CH_3_); ^13^C NMR (101 MHz, CDCl_3_) δ 162.7 (C=O), 138.2, 137.6 (C-2), 137.2, 134.5, 134.2, 131.2, 130.7, 129.8, 129.6, 127.9, 127.8, 127.7, 126.9 (ArC), 60.4 (OCH_2_CH_3_), 21.3 (ArCCH_3_), 14.2 (OCH_2_CH_3_); HRMS (ESI-TOF) *m*/*z*: [M + H]^+^ Calcd. for C_19_H_18_ClN_2_O_2_ 341.1051, found: 341.1058.

##### Ethyl 1-(4-Chlorophenyl)-5-(4-methylphenyl)-1*H*-imidazole-4-carboxylate **17k**

Yield: 661 mg, 38% as a pale yellow solid; mp: 161–163 °C; ^1^H NMR (400 MHz, CDCl_3_) δ 7.68 (1H, s, 2-CH), 7.28 (2H, d, *J* = 9.0 Hz, ArH), 7.09 (4H, m, ArH), 6.99–7.02 (2H, m, ArH), 4.26 (2H, q, *J* = 8.0 Hz, OCH_2_CH_3_), 2.31 (3H, s, ArCH_3_), 1.27 (3H, t, *J* = 7.0 Hz, OCH_2_CH_3_); ^13^C NMR (101 MHz, CDCl_3_) δ 162.7 (C=O), 138.9, 138.2 (C-2), 137.2, 134.4, 134.1, 130.5, 130.2, 129.6, 128.7, 126.7, 124.7 (ArC), 60.5 (OCH_2_CH_3_), 21.3 (ArCCH_3_), 14.2 (OCH_2_CH_3_); HRMS (ESI-TOF) *m*/*z*: [M + H]^+^ Calcd for C_18_H_15_ClFN_2_O_2_ 341.1051, found: 341.1052.

##### Ethyl 1-(4-Chlorophenyl)-5-phenyl-1*H*-imidazole-4-carboxylate **17l**

Yield: 534 mg, 32% as a yellow solid; mp: 133–135 °C; ^1^H NMR (400 MHz, CDCl_3_) δ 7.66 (1H, s, 2-CH), 7.23–7.25 (5H, m, ArH), 7.16 (2H, d, *J* = 7.2 Hz, ArH), 6.95 (2H, d, *J* = 8.8 Hz, ArH), 4.20 (2H, q, *J* = 7.0 Hz, OCH_2_CH_3_), 1.20 (3H, t, *J* = 7.0 Hz, OCH_2_CH_3_); ^13^C NMR (101 MHz, CDCl_3_) δ 162.6 (C=O), 137.9 (C-2), 137.3, 134.5, 134.0, 130.6, 130.6, 129.6, 128.9, 128.0, 127.8, 126.9 (ArC), 60.5 (OCH_2_CH_3_), 14.2 (OCH_2_CH_3_); HRMS (ESI-TOF) *m*/*z*: [M + H]^+^ Calcd for C_18_H_15_ClFN_2_O_2_ 327.0895, found: 327.0898.

#### 3.1.5. General Synthetic Procedure for the Preparation of 1,5-Diaryl-1*H*-imidazole-4-carboxylic Acids **10a**–**l**

NaOH (4 mmol) was added to a solution of ethyl 1,5-diaryl-1*H*-imidazoyl carboxylate **17** (2 mmol) in MeOH (10 mL) and H_2_O (10 mL). The resulting reaction mixture was heated under reflux until the disappearance of the starting material (monitored by TLC). MeOH solvent was then removed in vacuo and the remaining aqueous crude material was slowly neutralized with 1M HCl, followed by extraction with EtOAc (2 × 20 mL). The organic layer was then washed with brine, dried over MgSO_4_, and the solvent was removed in vacuo to produce the desired novel 1,5-diaryl-1*H*-imidazole-4-carboxylic acids **10**.

##### 1-(4-Bromophenyl)-5-(4-fluorophenyl)-1*H*-imidazole-4-carboxylic Acid **10a**

Yield: 491 mg, 68% as a pale yellow solid; ^1^H NMR (400 MHz, DMSO-*d*_6_) δ 12.3 (1H, br s, COOH), 8.06 (1H, s, 2-CH), 7.59 (2H, d, *J* = 8.4 Hz, ArH), 7.28 (2H, t, *J* = 7.2 Hz), 7.13–7.19 (4H, m, ArH), ^13^C NMR (101 MHz, DMSO-*d*_6_) δ 163.7 (C=O), 162.1 (d, ^1^*J_C_*_F_ = 246.1 Hz, FC-4′), 138.2 (C-2), 136.2, 134.7, (ArC), 133.1 (d, ^3^*J_CF_* = 9 Hz, C-2′ and C-6′), 132.3, 130.7, 128.4, 128.2 (ArC), 125.0 (d, ^4^*J_CF_* = 3 Hz, C-1′); 121.7 (ArC), 114.8 (d, ^3^*J_CF_* = 21 Hz, C-2′ and C-5′); HRMS (ESI-TOF) *m*/*z*: [M + H]^+^ Calcd, for C_16_H_11_^79^BrFN_2_O_2_ 360.9982, found 360.9991.

##### 1-(4-Bromophenyl)-5-(3-fluorophenyl)-1*H*-imidazole-4-carboxylic Acid **10b**

Yield: 520 mg, 72% as a pale yellow solid; ^1^H NMR (400 MHz, DMSO-*d*_6_) δ 12.3 (1H, br s, COOH), 8.08 (1H, s, 2-CH), 7.60 (2H, d, *J* = 8.8 Hz, ArH), 7.32 (1H, d, *J* = 6.8 Hz, ArH), 7.16–7.22 (4H, m, ArH), 7.01 (1H, d, *J* = 7.6 Hz, ArH); ^13^C NMR (101 MHz, DMSO-*d*_6_) δ 163.6 (C=O), 161.3 (d, *^1^J_CF_* = 244 Hz, FC-3′), 138.4 (C-2), 135.8, 136.3 (d, *^4^J_CF_* = 2 Hz, C-6′), 132.3 (ArC), 130.8 (d, ^3^*J_CF_* = 9 Hz, C-5′), 129.7 (d, ^3^*J_CF_* =9 Hz, C-1′), 128.4, 127.7, 127.0 (ArC), 117.9 (d, ^3^*J_CF_* = 23 Hz, C-4′), 115.4 (d, ^3^*J_CF_* = 22 Hz, C-2′); HRMS (ESI-TOF) *m*/*z*: [M + H]^+^ Calcd for C_16_H_11_^79^BrFN_2_O_2_ 360.9982, found 360.9987.

##### 1-(4-Bromophenyl)-5-(3-methoxyphenyl)-1*H*-imidazole-4-carboxylic Acid **10c**

Yield: 582 mg, 78% as a pale yellow solid; pale yellow solid; ^1^H NMR (400 MHz, DMSO-*d*_6_) δ 12.3 (1H, br s, COOH), 8.05 (1H, s, 2-CH), 7.59 (2H, d, *J* = 8.0 Hz, ArH), 7.18 (2H, t, *J* = 6.6 Hz, ArH), 6.87 (2H, d, *J* = 10.0 Hz, ArH), 6.75 (2H, d, *J* = 7.6 Hz, ArH) 3.66 (3H, s, ArOCH_3_); ^13^C NMR (101 MHz, DMSO-*d*_6_) δ 163.7 (C=O), 158.4 (ArCOCH_3_), 138.1 (C-2), 136.9, 134.9, 132.2, 130.5, 129.8, 128.9, 128.3, 123.1, 121.6, 116.8, 144.0, (ArC), 55.0 (ArCOCH_3_); H.RMS (ESI-TOF) *m*/*z*: [M + H]^+^ Calcd for C_17_H_14_^79^BrN_2_O_3_ 373.0182, Found 373.0201.

##### 1-(4-Bromophenyl)-5-(3-methylphenyl)-1*H*-imidazole-4-carboxylic Acid **10d**

Yield: 536 mg, 75% as a white solid; ^1^H NMR (400 MHz, DMSO-*d*_6_) δ 12.2 (1H, br s, COOH), 8.04 (1H, s, 2-CH), 7.58 (2H, d, *J* = 8.4 Hz, ArH), 7.10–7.19 (5H, m, ArH), 6.97 (1H, d, *J* = 7.2 Hz, ArH), 2.22 (3H, s, ArCH_3_); ^13^C NMR (101 MHz, DMSO-*d*_6_) δ 163.7 (C=O), 138.1 (C-2), 137.2, 136.8, 134.8, 132.2, 131.4, 130.5, 129.2, 128.5, 128.3, 127.8, 127.6, (ArC), 121.5 (ArCBr), 20.9 (ArCCH_3_); HRMS (ESI-TOF) *m*/*z*: [M + H]^+^ Calcd for C_17_H_14_^79^BrN_2_O_2_ 357.0233, found 357.0244.

##### 1-(4-Bromophenyl)-5-(4-methylphenyl)-1*H*-imidazole-4-carboxylic Acid **10e**

Yield: 500 mg, 70% as a pale yellow solid; ^1^H NMR (400 MHz, DMSO-*d*_6_) δ 12.2 (1H, br s, COOH), 8.04 (1H, s, 2-CH), 7.43 (2H, d, *J* = 8.6 Hz, ArH), 7.23 (2H, d, *J* = 8.5 Hz, ArH), 7.10 (4H, m, ArH), 2.26 (3H, s, ArCH_3_); ^13^C NMR (101 MHz, DMSO-*d*_6_) δ 163.8 (C=O), 138.1 (C-2),137.9, 137.3, 134.5, 133.1, 130.7, 130.4, 129.3, 128.4, 128.1, 125.6 (ArC), 20.9 (ArCCH_3_); HRMS (ESI-TOF) *m*/*z*: [M + H]^+^ Calcd for C_17_H_14_^79^BrN_2_O_2_ 357.0233, found 357.0246.

##### 1-(4-Bromophenyl)-5-phenyl-1*H*-imidazole-4-carboxylic Acid **10f**

Yield: 432 mg, 63% as a white solid; ^1^H NMR (400 MHz, DMSO-*d*_6_) δ 12.2 (1H, br s, COOH), 8.06 (1H, s, 2-CH), 7.57 (2H, d, *J* = 8.0 Hz, ArH), 7.15–7.31 (7H, m, ArH); ^13^C NMR (101 MHz, DMSO-*d*_6_) δ 163.7 (C=O), 138.2 (C-2), 137.1, 134.8, 132.2, 130.8, 130.5, 128.9, 128.5, 128.3, 127.8 (ArC), 121.5 (ArCBr); HRMS (ESI-TOF) *m*/*z*: [M + H]^+^ Calcd for C_16_H_12_^79^BrN_2_O_2_ 343.0077, found 343.0089.

##### 1-(4-Chlorophenyl)-5-(4-fluorophenyl)-1*H*-imidazole-4-carboxylic Acid **10g**

Yield: 437 mg, 69% as a pale yellow solid; ^1^H NMR (400 MHz, DMSO-*d*_6_) δ 8.10 (1H, s, 2-CH), 7.46 (2H, d, *J* = 8.4 Hz, ArH), 7.25–7.32 (4H, m, ArH), 7.13 (2H, t, *J* = 8.7 Hz, ArH), ^13^C NMR (101 MHz, DMSO-*d*_6_) δ 163.4 (C=O), 162.2 (d, ^1^*J_CF_* = 246.1 Hz, FC-4′), 138.3 (C-2), 136.3, 134.1, 133,3, (ArC), 133.2 (d, ^3^*J_CF_* = 8 Hz, C-2′ and C-6′), 131.8, 131.6, 129.4, 128.2 (ArC), 124.7 (d, ^4^*J_CF_* = 3 Hz, C-1′); 114.8 (d, ^3^*J_CF_* = 22.1 Hz, C-3′ and C-5′); HRMS (ESI-TOF) *m*/*z*: [M + H]^+^ Calcd, for C_16_H_11_ClFN_2_O_2_ 317.0488, found 317.0499.

##### 1-(4-Chlorophenyl)-5-(3-fluorophenyl)-1*H*-imidazole-4-carboxylic Acid **10h**

Yield: 443 mg, 70% as a pale yellow solid; ^1^H NMR (400 MHz, DMSO-*d*_6_) δ 7.93 (1H, s, 2-CH), 7.44 (2H, d, *J* = 7.6 Hz, ArH), 7.17–7.19 (4H, m, ArH), 6.90–7.06 (2H, m, ArH), ^13^C NMR (101 MHz, DMSO-*d*_6_) δ 161.4 (C=O), 161.2 (d, ^1^*J_CF_* = 244 Hz, FC-3′), 138.0 (C-2), 134.7 (ArC), 132.4 (d, ^3^J_CF_ = 4 Hz, C-6′), 132. 1, 132.2 (ArC), 129.2 (d, ^3^*J_CF_* = 9 Hz, C-5′), 129.4 (d, ^3^*J_CF_* = 8 Hz, C-1′), 128.2, 127.0, 121.5 (ArC), 118.1 (d, ^3^*J_CF_* = 23 Hz, C-4′), 115.1 (d, ^3^*J_CF_* = 21 Hz, C-2′); HRMS (ESI-TOF) *m*/*z*: [M + H]^+^ Calcd, for C_16_H_11_ClFN_2_O_2_ 317.0488, found 317.0497.

##### 1-(4-Chlorophenyl)-5-(3-methoxyphenyl)-1*H*-imidazole-4-carboxylic Acid **10i**

Yield: 526 mg, 80% as a white solid; ^1^H NMR (400 MHz, DMSO-*d*_6_) δ 11.9 (1H, br s, COOH), 9.23 (1H, s, 2-CH), 7.52 (2H, d, *J* = 8.4 Hz, ArH), 7.41 (2H, d, *J* = 8.4 Hz, ArH), 7.22 (1H, t, *J* = 8.0 Hz, ArH), 6.93 (2H, d, *J* = 6.2 Hz, ArH), 6.84 (2H, d, *J* = 6.2 Hz, ArH), 3.65 (3H, s, ArOCH_3_); ^13^C NMR (101 MHz, DMSO-*d*_6_) δ 160.6 (C=O), 158.5 (ArCOCH_3_), 138.0 (C-2), 137.3, 134.4, 133.1, 129.4, 129.2, 128.7, 127.2, 124.7, 123.1, 117.0, 115.1 (ArC), 55.1 (ArCOCH_3_); HRMS (ESI-TOF) *m*/*z*: [M + H]^+^ Calcd, for C_17_H_14_ClN_2_O_3_ 329.0687, found 329.0693.

##### 1-(4-Chlorophenyl)-5-(3-methylphenyl)-1*H*-imidazole-4-carboxylic Acid **10j**

Yield: 519 mg, 83% as a pale yellow solid; ^1^H NMR (400 MHz, DMSO-*d*_6_) δ 8.05 (1H, s, 2-CH), 7.44 (2H, d, *J* = 8.7 Hz, ArH), 7.23 (2H, d, *J* = 8.7 Hz, ArH), 7.09 (3H, m, ArH), 6.98 (1H, d, *J* = 7.4 Hz, ArH), 2.22 (3H, s, ArCH_3_); ^13^C NMR (101 MHz, DMSO-*d*_6_) δ 163.7 (C=O), 138.1 (C-2), 137.3, 136.8, 134.4, 133.1, 131.4, 130.3, 130.4, 129.2, 128.5, 128.0, 127.9, 127.6 (ArC), 20.9 (ArCCH_3_); HRMS (ESI-TOF) *m*/*z*: [M + H]^+^ Calcd, for C_17_H_14_ClN_2_O_2_ 313.0738, found 313.0744.

##### 1-(4-Chlorophenyl)-5-(4-methylphenyl)-1*H*-imidazole-4-carboxylic Acid **10k**

Yield: 494 mg, 79% as a pale yellow solid; ^1^H NMR (400 MHz, DMSO-*d*_6_) δ 12.0 (1H, br s, COOH), 9.38 (1H, s, 2-CH), 7.51 (2H, d, *J* = 8.5 Hz, ArH), 7.41 (2H, d, *J* = 8.5 Hz, ArH), 7.19 (2H, d, *J* = 8.0 Hz, ArH), 7.13 (2H, d, *J* = 8.0 Hz, ArH), 2.26 (3H, s, ArCH_3_); ^13^C NMR (101 MHz, DMSO-*d*_6_) δ 160.3 (C=O), 139.5, 138.0 (C-2), 137.9, 134.5, 133.0, 131.0, 129.4, 128.8, 128.7, 123.9, 122.7 (ArC), 20.9 (ArCCH_3_); HRMS (ESI-TOF) *m*/*z*: [M + H]^+^ Calcd, for C_17_H_14_ClN_2_O_2_ 313.0738, found 313.0747.

##### 1-(4-Chlorophenyl)-5-phenyl-1*H*-imidazole-4-carboxylic Acid **10l**

Yield: 460 mg, 77% as a white solid; ^1^H NMR (400 MHz, DMSO-*d*_6_) δ 8.75 (1H, s, 2-CH), 7.46 (2H, d, *J* = 10 Hz, ArH), 7.26–7.36 (7H, m, ArH); ^13^C NMR (101 MHz, DMSO-*d*_6_) δ 162.1 (C=O), 138.3 (C-2), 137.9, 134.4, 133.7, 131.2, 129.7, 128.7, 128.4, 128.3, 127.1, 126.9 (ArC); HRMS (ESI-TOF) *m*/*z*: [M + H]^+^ Calcd, for C_16_H_12_ClN_2_O_2_ 299.0582, found 299.0587.

#### 3.1.6. General Procedure for the Synthesis of 1,5-diaryl-1*H*-imidazole-4-carbohydrazide Derivatives **11a**–**b**, **11d**–**e**, **11g**–**l**

Ethyl 1,5-diaryl-1*H*-imidazole-4-carboxylate derivative **17** (2.55 mmol) was placed in a 25 mL carousel vessel followed by the addition of ethanol (15 mL) and hydrazine hydrate monohydrate (33.2 mmol). The resulting reaction mixture was heated at reflux using a Carousel 12 Plus Reaction Station™ set at 100 °C with stirring. After 24 h, the solution was allowed to cool to room temperature to form a precipitated solid which was collected by filtration and washed with a small amount of cooled ethyl alcohol and dried under vacuum to afford 1,5-diaryl-1*H*-imidazole-4- carbohydrazide derivative **11**.

##### 1-(4-Bromophenyl)-5-(4-fluorophenyl)-1*H*-imidazole-4-carbohydrazide **11a**

Yield: 727 mg, 76% as a white solid; ^1^H NMR (400 MHz, DMSO-*d*_6_) δ 9.24 (1H, br s, NH), 8.06 (1H, s, 2-CH), 7.63 (2H, d, *J* = 8.8 Hz, ArH), 7.26 (2H, t, *J* = 8.7 Hz, ArH). 7.10–7.19 (4H, m, ArH), 4.42 (1H, br s, NH_2_); ^13^C NMR (101 MHz, DMSO-*d*_6_) δ 161.4 (C=O), 161.9 (d, ^1^*J_CF_* = 244 Hz, FC-3′), 137.7 (C-2), 134.8, 133.2 (ArC), 133.2 (d, ^3^*J_CF_* = 9 Hz, C-2′ and C-6′), 132.6, 132.3,132.0, 128.2 (ArC), 124.7 (d, ^4^*J_CF_* = 3 Hz, C-1′); 121.5 (ArC), 114.5 (d, ^3^*J_CF_* = 22 Hz, C-4′); HRMS (ESI-TOF) *m*/*z*: [M + H]^+^ Calcd for C_16_H_13_F^79^BrN_4_O 375.0251, measured 375.0255.

##### 1-(4-Bromophenyl)-5-(3-fluorophenyl)-1*H*-imidazole-4-carbohydrazide **11b**

Yield: 698 mg, 73% as a white solid; ^1^H NMR (400 MHz, DMSO-*d*_6_) δ 9.25 (1H, br s, NH), 8.08 (1H, s, 2-CH), 7.61 (2H, d, *J* = 7.6 Hz, ArH), 7.27 (2H, d, *J* = 8.0 Hz, ArH), 7.12–7.22 (4H, m, ArH), 6.96 (2H, d, *J* = 8.0 Hz, ArH), 4.42 (1H, br s, NH_2_); ^13^C NMR (101 MHz, DMSO-*d*_6_) δ161.4 (C=O), 161.2 (d, ^1^*J_CF_* = 244 Hz, FC-3′), 137.9 (C-2), 134.7, 132.4, 133.2 (d, ^4^J_CF_ = 3 Hz, C-6′), 132.1 (ArC), 130.6 (d, ^3^*J_CF_* = 9 Hz, C-1′), 129.4 (d, ^3^*J_CF_* = 8 Hz, C-5′), 128.2, 126.9, 121.5 (ArC), 118.1 (d, ^3^*J_CF_* = 23 Hz, C-2′), 115.1 (d, ^3^*J_C_*_F_ = 21 Hz, C-4′); HRMS (ESI-TOF) *m*/*z*: [M + H]^+^ Calcd for C_16_H_13_F^79^BrN_4_O 375.0251, measured 375.0250.

##### 1-(4-Bromophenyl)-5-(3-methylphenyl)-1*H*-imidazole-4-carbohydrazide **11d**

Yield: 644 mg, 68% as a white solid; ^1^H NMR (400 MHz, DMSO-*d*_6_) δ 9.20 (1H, br s, NH), 8.07 (1H, s, 2-CH), 7.62 (2H, d, *J* = 8.8 Hz, ArH), 7.16–7.20 (5H, m, ArH), 6.96 (1H, d, *J* = 4.4 Hz, ArH), 4.42 (2H, br s, NH_2_), 2.26 (3H, s, ArCH_3_); ^13^C NMR (101 MHz, DMSO-*d*_6_) δ 161.6 (C=O), 137.6 (C-2), 136.6, 135.0, 133.6, 132.2, 131.9, 131.6, 128.9, 128.2, 128.1, 127.8, 127.5, 121.3 (ArC), 21.0 (ArCCH_3_); HRMS (ESI-TOF) *m*/*z*: [M + H]^+^ Calcd for C_17_H_16_^79^BrN_4_O 371.0502, measured 371.0499.

##### 1-(4-Bromophenyl)-5-(4-methylphenyl)-1*H*-imidazole-4-carbohydrazide **11e**

Yield: 606 mg, 64% as a white solid; ^1^H NMR (400 MHz, DMSO-*d*_6_) δ 9.16 (1H, br s, NH), 8.05 (1H, s, 2-CH), 7.49 (2H, d, *J* = 8.5 Hz, ArH), 7.24 (2H, d, *J* = 8.5 Hz, ArH), 7.09–7.15 (4H, m, ArH), 4.34 (2H, s, NH_2_), 2.29 (3H, s, ArCH_3_); ^13^C NMR (101 MHz, DMSO-*d*_6_) δ 161.7 (C=O), 137.6 (C-2), 137.6, 134.6, 133.6, 132.9, 131.8, 130.8, 129.3, 128.2, 127.9, 125.3 (ArC), 20.8 (ArCCH_3_); HRMS (ESI-TOF) *m*/*z*: [M + H]^+^ Calcd for C_17_H_16_^79^BrN_4_O 371.0502, measured 371.0504.

##### 1-(4-Chlorophenyl)-5-(4-fluorophenyl)-1*H*-imidazole-4-carbohydrazide **11g**

Yield: 557 mg, 66% as a white solid; ^1^H NMR (400 MHz, DMSO-*d*_6_) δ 9.23 (1H, br s, NH), 8.06 (1H, s, 2-CH), 7.47 (2H, d, *J* = 8.8 Hz, ArH), 7.23–7.31 (4H, m, ArH), 7.11 (2H, t, *J* = 8.7 Hz, ArH), 4.37 (1H, br s, NH_2_); ^13^C NMR (101 MHz, DMSO-*d*_6_) δ 163.1 (C=O), 160.1 (d, ^1^J_CF_ = 246 Hz, FC-4′), 137.7 (C-2), 134.3 (ArC), 133.2 (d, ^3^*J_CF_* = 8 Hz, C-2′ and C-6′), 133.0, 129.4, 132.6, 132.0, 129.4, 128.0 (ArC), 124.7 (d, ^4^*J_CF_* = 4 Hz, C-1′); 114.5 (d, ^3^*J_CF_* = 21 Hz, C-3′ and C-5′); HRMS (ESI-TOF) *m*/*z*: [M + H]^+^ Calcd for C_16_H_13_FClN_4_O 331.0756, measured 331.0850.

##### 1-(4-Chlorophenyl)-5-(3-fluorophenyl)-1*H*-imidazole-4-carbohydrazide **11h**

Yield: 615 mg, 73% as a white solid; ^1^H NMR (400 MHz, DMSO-*d*_6_) δ 9.26 (1H, br s, NH), 8.08 (1H, s, 2-CH), 7.48 (2H, d, *J* = 8 Hz, ArH), 7.26 (3H, t, *J* = 8 Hz, ArH), 7.15 (2H, dd, *J* = 8 Hz, ArH), 6.95 (1H, d, *J* = 8 Hz, ArH), 4.37 (1H, br s, NH_2_); ^13^C NMR (101 MHz, DMSO-*d*_6_) δ 162.1 (C=O), 161.9 (d, ^1^*J_CF_* = 244 Hz, FC-3′), 138.72 (C-2), 134.3, 134.1, 133.0, 132.2 (d, ^4^*J_CF_* = 3 Hz, C-6′), 130.6 (d, ^3^*J_CF_* = 8Hz, C-1′), 129.3 (d, ^3^J_CF_ = 8 Hz, C-5′), 128.2, 128.0, 127.0 (ArC), 118.1 (d, ^3^*J_CF_* = 22 Hz, C-2′), 115.5 (d, ^3^*J_CF_* = 20 Hz, C-4′); HRMS (ESI-TOF) *m*/*z*: [M + H]^+^ Calcd for C_16_H_13_FClN_4_O 331.0756, measured 331.0767.

##### 1-(4-Chlorophenyl)-5-(3-methoxyphenyl)-1*H*-imidazole-4-carbohydrazide **11i**

Yield: 594 mg, 68% as a white solid; ^1^H NMR (400 MHz, DMSO-*d*_6_) δ 9.19 (1H, br s, NH), 8.05 (1H, s, 2-CH), 7.48 (2H, d, *J* = 2.0 Hz, ArH), 7.46 (2H, d, *J* = 2.0 Hz, ArH), 7.15–6.90 (4H, m, ArH), 4.34 (2H, br s, NH_2_), 3.66 (3H, s, ArOCH_3_); ^13^C NMR (101 MHz, DMSO-*d*_6_) δ 161.6 (C=O), 158.3 (ArCOCH_3_), 137.7 (C-2), 134.6, 133.3, 132.9, 132.0, 129.5, 129.3, 128.6, 127.9, 123.1, 116.9, 113.8 (ArC), 54.9 (ArCOCH_3_); HRMS (ESI-TOF) *m*/*z*: [M + H]^+^ Calcd for C_17_H_16_ClN_4_O_2_ 343.0956, measured 343.0957.

##### 1-(4-Chlorophenyl)-5-(3-methylphenyl)-1*H*-imidazole-4-carbohydrazide **11j**

Yield: 517 mg, 62% as a white solid; ^1^H NMR (400 MHz, DMSO-*d*_6_) δ 9.18 (1H, br s, NH), 8.06 (1H, s, 2-CH), 7.47 (2H, d, *J* = 8.8 Hz, ArH), 7.23 (2H, d, *J* = 8.8 Hz, ArH), 7.16 (3H, m, ArH), 7.13 (1H, d, *J* = 4.4 Hz, ArH), 4.47 (2H, br s, NH_2_), 2.24 (3H, s, ArCH_3_); ^13^C NMR (101 MHz, DMSO-*d*_6_) δ 161.6 (C=O), 137.6 (C-2), 136.6, 134.6, 133.7, 132.9, 132.0, 131.9, 131.7, 129.3, 128.9, 128.3, 127.9, 127.5 (ArC), 21.0 (ArCCH_3_). HRMS (ESI-TOF) *m*/*z*: [M + H]^+^ Calcd for C_17_H_16_ClN_4_O 327.1007, measured 327.1013.

##### 1-(4-Chlorophenyl)-5-(4-methylphenyl)-1*H*-imidazole-4-carbohydrazide **11k**

Yield: 528 mg, 63% as a white solid; ^1^H NMR (400 MHz, DMSO-*d*_6_): δ = 9.13 (1H, br s, NH), 8.02 (1H, s, 2-CH), 7.46 (2H, d, *J* = 8.8 Hz, ArH), 7.21 (2H, d, *J* = 8.4 Hz, ArH), 7.07–7.13 (4H, m, ArH), 4.31 (2H, br s, NH_2_), 2.28 (3H, s, ArCH_3_); ^13^C NMR (101 MHz, DMSO-*d*_6_) δ 161.7 (C=O), 137.6 (C-2), 137.5, 134.6, 133.6, 132.9, 131.8, 130.8, 129.3, 128.2, 127.9, 125.3 (ArC), 20.8 (ArCCH_3_); HRMS (ESI-TOF) *m*/*z*: [M + H]^+^ Calcd for C_17_H_16_ClN_4_O 327.1007, measured 327.1011.

##### 1-(4-Chlorophenyl)-5-phenyl-1*H*-imidazole-4-carbohydrazide **11l**

Yield: 463 mg, 58% as a white solid; ^1^H NMR (400 MHz, DMSO-*d*_6_) δ 9.18 (1H, br s, NH), 8.06 (1H, s, 2-CH), 7.45 (2H, d, *J* = 8.8 Hz, ArH), 7.22–7.30 (7H, m, ArH), 4.33 (2H, br s, NH_2_); ^13^C NMR (101 MHz, DMSO-*d*_6_) δ 161.6 (C=O), 137.7 (C-2), 134.5, 133.5, 132.9, 132.0, 130.9, 129.3, 128.3, 128.2, 127.9, 127.6 (ArC). HRMS (ESI-TOF) *m*/*z*: [M + H]^+^ Calcd for C_16_H_14_ClN_4_O 313.0851, measured 313.0841.

### 3.2. Modeling Protocols

The X-ray crystal structure of HIV-1 IN dimer (PDB code: 4NYF) in complex with the small molecule, BI 224436 (2) was used for a better understanding of binding interactions of the tested ligands [27]. The HIV-1 IN protein used in the modeling was prepared using the “Protein Preparation Wizard” in Maestro (Schrodinger suite, Schrödinger, Inc, Cambridge, MA, USA) [66]. This protocol involves the addition of hydrogen atoms, bond order assignment, and optimization of bond lengths, bond angles, torsion angles, and non-bonded interactions using the OPLS force field. Furthermore, missing loops and side chains were filled in using Prime with a length of axes set to 30 Å and a value of the scaling factor chosen to be 1.00. Docking grids were produced with the default settings in Glide utilizing the co-crystalized ligand, BI-224436 binding site to express the focus of the grid box. Before docking, structures were minimized to their lowest energy and all possible 3D structure of molecules were created utilizing Ligprep protocol in Maestro [67]. The protocol encompasses the addition of hydrogen, transforming 2D structures to 3D, creating stereoisomers, neutralizing charges, and forecasting the probable ionization state using an OPLS3 force field. The scaffolds were then primarily positioned to the LEDGF/p75 binding site in HIV-1 integrase using the Schrödinger Glide protocol with the standard precision (SP) mode, which resulted in the generation of hundreds for poses of each compound [68].

### 3.3. Biological Evaluation Protocols

#### 3.3.1. HIV-1 IN-LEDGF/p75 AlphaScreen^TM^ Assay

An AlphaScreen^TM^ assay was developed and used to determine the percentage inhibition of the newly synthesized 1,5-diaryl-1*H*-imidazole-4-carboxylic acids **10** and 1,5-diaryl-1*H*-imidazole-4-carbohydrazide derivatives **11**. Assay buffer (50 mM Tris pH 7.4, 1 mM MgCl_2_, 1 mM DTT, 0.01% Tween 20, and 2.5% Glycerol) was used to make 10× working stocks of FLAG-IN, His-LEDGF/p75 and test compounds with concentrations of 1 µM, 5 µM, and 1 mM, respectively. Total reaction volumes were 50 µL. First, 35 µL of assay buffer was added to wells of a 1/2 area optiplate. Then, 5 µL of a working stock of FLAG-tagged IN was added to each well containing assay buffer, except for control wells. Then, 5 µL of test compounds were added to each well containing FLAG-IN and assay buffer, except for control wells. Plates were incubated for 30 min at room temperature with gentle shaking. Subsequently, 5 µL of His-tagged LEDGF/p75 was added to each test well, except to control wells, and incubated for 1 h at 30 °C with gentle shaking. A final concentration of 10 µg/mL nickel chelating acceptor beads (Perkin Elmer, MA, USA) and Anti-FLAG donor beads (Perkin Elmer, MA, USA) were added and incubated in the dark at 30 °C with gentle shaking. The plate was subsequently read between 520–620 nm on the EnSpire^®^ plate reader (Perkin Elmer, MA, USA). The control (3) ran on the plate to ensure accurate reading of the compounds effects were: beads alone, FLAG-IN plus beads alone, His-LEDGF/p75 plus beads alone, FLAG-IN, and His-LEDGF/p75 alone.

#### 3.3.2. Strand Transfer Assay

Reaction buffer (50 mM HEPES pH 7.5, 10 mM NaCl, 5 mM MnCl_2_/MgCl_2_, 15 mM DTT) was added to wells of a round bottom 96 well plate at 10 μL per well. IN and donor DNA were mixed in reaction buffer and 10 μL each was added to the wells containing pre-aliquoted reaction buffer. The final concentrations of IN and donor DNA was 200 nM and 100 nM, respectively, in the context of the total reaction volume. The IN was allowed to complex the DNA for 15 min. Tested compounds were added at 10 µL, to a final of 100 µM, and mixed in wells containing pre-aliquoted reaction buffer. This was incubated at ambient temperature for 15 min. Target DNA was added in a 10 μL volume to a 200 nM final concentration. Total reaction volume was 50 µL. The reaction was immediately incubated at 37 °C for 90 min in a shaking plate incubator. Reactions were stopped by addition of 50 μL ELISA Binding buffer (50 mM Tris-HCl pH 7.5, 4M NaCl, 20 mM EDTA), and immediately added to Streptavidin coated plates (RD Biosystems) and incubated at ambient temperature for 30 min. Non-bound reaction products were removed by washing the wells with TBS-T (25 mM Tris- HCl pH 7.5, 150 mM NaCl, Tween 0.005%) two times with no incubations between washes. Anti FITC-AP linked antibody (Sigma-Aldrich) was added at 200 μL at a 1:5000 dilution in TBS-T and incubated at 37 °C for 120 min. The wells were then washed three times with TBS-T with 5 min incubation per wash. SIGMAFASTTM *p*-Nitrophenyl phosphate Tablets (Sigma-Aldrich), a phosphatase substrate, was prepared and added to each test well at 100 μL and incubated at 37 °C for 30 min. Absorbance readings were determined at 405 nm on the X-mark spectrophotometer (Bio-Rad, Rosebank, South Africa) [59].

#### 3.3.3. Cellular Toxicity Assay

The day of cytotoxicity testing, MT-4 cells (obtained through the NIH AIDS Reagent Program, Division of AIDS, NIAID, NIH: MT-4 catalogue number 120) were counted and seeded at 5 × 10^4^ cells per well. A total of 100 µL of cells were added to each well of a 96-well cell culture plates (TTP) for testing. The volume in the last row of wells of each plate was further increased to 197 µL of media containing MT-4 cells. The plate was placed into the incubator to equilibrate to 37 °C and 5% CO_2_. Test compounds were prepared in plate format and each compound was at 25 mM stock concentration in DMSO. A total of 3.2 μL compound was added to the final row of wells containing 197 µL cells and mixed to ensure the solution was homogeneous with the cells. A 2-fold serial dilution of an 8 times series was performed and the final 100 µL was discarded. The plate was placed at 37 °C and 5% CO_2_ for 5 days. On the 5th day the cell viability was assessed using the Cell Titre 96^®^ Aqueous One Solution Cell Proliferation assay (Promega) as per manufacturer’s instructions. Briefly, 20 µL of MTS solution was added to each well and incubated at 37 °C for 4 h. The absorbance of the plates was then read at 490 nm on a SpectraMax spectrophotometer (Molecular Devices, LLC. San Jose, CA, USA). The data analysis was completed on Origin 8.1 with the log value of the compound molar concentration plotted against the percentage cell viability determined by the absorbance level by sigmoidal fitting. From the curve, a half maximal cytotoxicity CC_50_ for compounds was determined. Auranofin was used as a cytotoxic control compound starting at 100 µM across a two-fold serial dilution of an 8 dilution series. DMSO was used as background control and 2-fold serially diluted from 1.5% [60].

#### 3.3.4. Antiviral Assay

A subtype C virus isolate was obtained through the NIH HIV Reagent Program, Division of AIDS, NIAID, NIH: Human Immunodeficiency Virus Type 1 strain 98/IN/022, ARP-4167, contributed by UNAIDS Network for HIV Isolation and Characterization. MT-4 cells were grown and maintained in RPMI (Gibco) supplemented with 2 mM L-glutamine, non-essential amino acids and 10% heat inactivated fetal bovine serum at 37 °C and 5% CO_2_. Subtype C virus was added to MT4 cells and incubated at 37 °C and 5% CO_2_ for two weeks. Supernatants were harvested by centrifugation at 4000× *g* and stored at −80 °C until use. Harvested supernatant containing HIV-1 was added to fresh MT-4 cells and viral absorption was allowed for 24 h similar to the method by Rosenwirth et al. [69]. After viral absorption, the cells were washed once with fresh media, a sample was taken, and HIV-1 infection was allowed to develop. Seven days after infection, MT-4 supernatant samples were tested for the presence of p24 antigen using the bioelisa HIV-1+2 Ag/Ab kit (biokit) as per manufacturer’s instructions. After HIV-1 infection was confirmed, supernatant samples were isolated by centrifugation at 4000× *g* and immediately used to infect 1 × 10^5^ cells/mL of MT4 cells. Virus was allowed to absorb and 24 h later the cells were washed with fresh media and 100 µL of cells, 1 × 10^4^ cells, were aliquoted into a round bottom 96 well plate (TPP). Uninfected cells were also prepared. A day 1 sample was taken and stored at −80 °C until final testing. Compounds were diluted in fresh media to 200 µM concentrations and 100 µL was added to each test well, the final concentration of compounds was 100 µM. Raltegravir (NIH, ARP-11680) was used as a control compound at 10 µM, blank wells contained equal volumes of DMSO. Upper and lower asymptotes were determined by untreated MT4 cells either infected with HIV (100%) or not infected with HIV (0%). Four days after infection, p24 presence was determined using the bioelisa HIV-1+2 Ag/Ab kit as per manufacturer’s instructions. The data was used to determine if the selected concentration of compounds would reduce the amount of p24 and thereby indicate a reduction in HIV infectivity [62].

#### 3.3.5. Multimerization

Crosslinking reactions were prepared in Oligomerization Buffer [50 mM HEPES, pH 7.1, 300 mM NaCl, 2 mM MgCl_2_, 1 mM DTT] with varying concentrations of compounds and IN protein. Tested compounds were used at a range of 50 µM, 100 µM, and 200 µM. Mut-101 was included as a control and used at 50 µM only. IN enzymes were incubated with compounds for 30 min at ambient temperature. The bi-functional reagent BS3 (Thermo-Pierce, IL, USA) was added at 12.5 μM final concentration, and the mixture was incubated for a further 30 min at ambient temperature. Reactions were stopped by the addition of Tris-HCl to 50 mM final concentration, and incubated at ambient temperature for a final 15 min. Samples were boiled in SDS-PAGE loading buffer under reducing conditions. IN multimers were visualized by SDS-PAGE and Western blot analysis [63].

## 4. Conclusions

A series of novel 1,5-diaryl-1*H*-imidazole-4-carboxylic acids **10** and 1,5-diaryl-1*H*-imidazole-4-carbohydrazides **11** were designed and synthesized via the formation of 1,5-diaryl-1*H*-imidazole-4-carboxylate ester intermediates **17** obtained from cycloaddition of ethyl isocyanoacetate **16** and stable diarylimidoyl chlorides **15** under basic conditions. This study was conducted to evaluate the hypothesis that the HIV-1 IN activity of the hit compound **9** could be greatly improved through the addition of carboxylic acid and carbohydrazide motifs. This idea was demonstrated to be productive, since seventeen scaffolds (i.e., **10a**–**d**, **10f**, **10h**–**k**, **11a**–**b**, **11d**–**e**, **11h**–**k**) exhibited more than 50% inhibitory activity in disrupting the HIV-1 IN- LEDGF/p75 protein-protein interaction in a direct AlphaScreen^TM^ assay at 100 μM. The specific binding of these compounds to the LEDGF/p75-binding pocket was also established by their further assessment in the HIV-1 IN strand transfer assay at 100 μM, which concluded that none of the compounds (with the exception of **10a**, **10l**, and **11k** with marginal inhibitory percentage activities of 44%, 39%, and 43%, respectively) were actively bound to the active site. In a cell-based assay, the newly targeted compounds exhibited varying degrees of toxicity, with 13 compounds having a CC_50_ value of greater than or equal to 100 µM, while 9 compounds were found to exhibit CC_50_ values of below 100 µM. Further evaluation of the compounds in an antiviral HIV-1 assay revealed four compounds (**11a**, **11b**, **11g**, and **11h**) with moderate antiviral percentage activities ranging from 33 to 45% and with CC_50_ values ranging from 50.4 µM to >200 µM. The antiviral percentage activity displayed by **11h** was credited to its relatively high toxicity of 50.4 µM. Compounds **11a**, **11b**, **11g**, and **11h** were further validated for their ability to induce multimerization in a Western blot gel assay, and three compounds, **11a**, **11b**, and **11h** seemed to increase higher-order forms of IN. These data serve as a good starting point for the further optimization of these scaffolds as possible anti-HIV-1 IN drugs. Therefore, efforts are currently underway in our laboratories to improve the potency and enhance the drug-like properties of these compounds.

## Figures and Tables

**Scheme 4:**
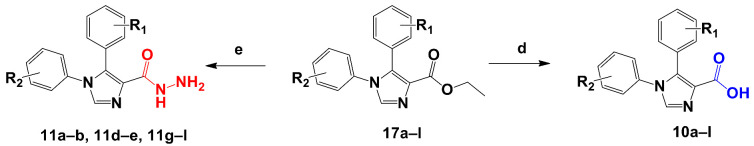
Synthesis of compounds **10** and **11**. Reagents and conditions: (d) i: NaOH, H_2_O, MeOH [40], ii: HCl, H_2_O; (e) NH_2_NH_2_.H_2_O, EtOH, reflux, 24 h [57].

**Table 1:** Isolated yields of ethyl 1,5-diaryl-1*H*-imidazole-4-carboxylates **17a**–**l**.

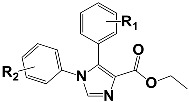			
Compound	R_1_	R_2_	Yield % ^1^
**17a**	4-F	4-Br	64
**17b**	3-F	4-Br	58
**17c**	3-OMe	4-Br	18
**17d**	3-Me	4-Br	30
**17e**	4-Me	4-Br	35
**17f**	H	4-Br	22
**17g**	4-F	4-Cl	72
**17h**	3-F	4-Cl	67
**17i**	3-OMe	4-Cl	32
**17j**	3-Me	4-Cl	41
**17k**	4-Me	4-Cl	38
**17l**	H	4-Cl	42

^1^ Yields were determined after column chromatographic purification.

**Table 2:** Isolated yields of 1,5-diaryl-1*H*-imidazole-4-carboxylic acids **10** and 1,5-diaryl-1*H*-imidazole-4-carbohydrazides **11**.

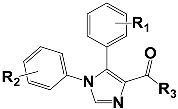				
Compound	R_1_	R_2_	R_3_	Yield %
**10a**	4-F	4-Br	OH	69
**10b**	3-F	4-Br	OH	72
**10c**	3-OMe	4-Br	OH	78
**10d**	3-Me	4-Br	OH	75
**10e**	4-Me	4-Br	OH	70
**10f**	H	4-Br	OH	63
**10g**	4-F	4-Cl	OH	69
**10h**	3-F	4-Cl	OH	70
**10i**	3-OMe	4-Cl	OH	80
**10j**	3-Me	4-Cl	OH	83
**10k**	4-Me	4-Cl	OH	79
**10l**	H	4-Cl	OH	77
**11a**	4-F	4-Br	NHNH_2_	76
**11b**	3-F	4-Br	NHNH_2_	73
**11d**	3-Me	4-Br	NHNH_2_	64
**11e**	4-Me	4-Br	NHNH_2_	68
**11g**	4-F	4-Cl	NHNH_2_	66
**11h**	3-F	4-Cl	NHNH_2_	73
**11i**	3-OMe	4-Cl	NHNH_2_	68
**11j**	3-Me	4-Cl	NHNH_2_	62
**11k**	4-Me	4-Cl	NHNH_2_	63
**11l**	H	4-Cl	NHNH_2_	58

**Table 3:** The HIV-1 IN-LEDGF/p75 inhibition activities observed for 1,5-diaryl-1*H*-imidazole-4-carboxylic acids **10** and 1,5-diaryl-1*H*-imidazole-4-carbohydrazides **11**.

Compound	Biochemical Assays	
	HIV-1 IN-LEDGF (%) Inhibition at 100 µM (AlphaScreen^TM^) ^1^*	Strand Transfer Activity (%) Inhibition at 100 µM (ELISA) ^1^*
**10a**	83 ± 3.36	44 ± 1.13
**10b**	76 ± 2.83	24 ± 3.39
**10c**	51 ± 4.16	0
**10d**	84 ± 0.29	9 ± 9.66
**10e**	82 ± 0.36	2 ± 9.00
**10f**	17 ± 2.55	16 ± 4.89
**10g**	47 ± 3.44	0
**10h**	54 ± 10.05	0
**10i**	74 ± 9.29	0
**10j**	78 ± 8.99	12 ± 4.26
**10k**	85 ± 3.79	1.4 ± 6.07
**10l**	35 ± 2.26	39 ± 1.80
**11a**	51 ± 9.16	0
**11b**	50 ± 2.01	0
**11d**	67 ± 9.00	1 ± 5.89
**11e**	89 ± 1.38	0
**11g**	0	0
**11h**	59 ± 3.22	0
**11i**	50 ± 9.61	0
**11j**	33 ± 6.89	9±
**11k**	78 ± 8.96	43 ± 6.20
**11l**	0	0
**MUT 101**	90 ± 0.52	-
**Raltegravir**	-	95 ± 0.98

^1^* All compounds were tested at 100 µM concentration in both the AlphaScreen and the ELISA assay unless otherwise stated.

**Table 4:** The HIV-1 IN-LEDGF/p75 cell-based assay results for 1,5-diaryl-1*H*-imidazole-4-carboxylic acids **10** and 1,5-diaryl-1*H*-imidazole-4-carbohydrazide **11**.

Compound	Antiviral Activity at 100 µM (%) HIV-1 Subtype C ^1^*	Cytotoxicity CC_50_ (μM)
**10a**	15	113.9
**10b**	9	67.1
**10c**	32	81.5
**10d**	11	100.1
**10e**	20	108.1
**10f**	0	39.0
**10g**	0	199.7
**10h**	4	168.8
**10i**	0	>200
**10j**	0	135.7
**10k**	5	103.2
**10l**	0	>200
**11a**	36	>200
**11b**	35	158.2
**11d**	33	58.4
**11e**	24	78.6
**11g**	31	>200
**11h**	40	50.4
**11i**	10	91.2
**11j**	18	37.9
**11k**	23	47.6
**11l**	26	172.7
**Raltegravir**	83 at 10 µM	-
**Auranofin**		1.6

^1^* All compounds tested at 100 µM unless otherwise stated.

## Data Availability

Data is contained within the article or Supplementary Material.

## Supplementary Materials

The following are available online. ^1^H- and ^13^C-NMR spectra (Figures S1–S68) of all novel synthesized molecules.

## Sample Availability

Samples of the compounds are available on request from the corresponding authors.
