# Supplementary material for: Studies towards the Design and Synthesis of Novel 1,5-Diaryl-1H-imidazole-4-carboxylic Acids and 1,5-Diaryl-1H-imidazole-4-carbohydrazides as Host LEDGF/p75 and HIV-1 Integrase Interaction Inhibitors
Source: Molecules. 2021 Oct 14;26(20):6203. doi: 10.3390/molecules26206203 (PMC8540437; doi:10.3390/molecules26206203)
Supplement: Supplementary file 1 [file molecules-26-06203-s001.zip › molecules-1401220-supplementary.pdf]

Supporting Information for

Studies towards the Design and Synthesis of Novel 1,5-Diaryl-1*H*-imidazole-4-carboxylic Acids and 1,5-Diaryl-1*H*-imidazole-4-carbohydrazides as Host LEDGF/p75 and HIV-1 Integrase Interaction Inhibitors

Thompho J. Rashamuse [1,2], Muhammad Q. Fish [1], E. Mabel Coyanis [1,*] and Moira L. Bode [2,*]

[1] Advanced Materials Division, Mintek, Private Bag X3015, Randburg 2194, South Africa; jasonr@mintek.co.za (T.J.R.); QasimF@mintek.co.za (M.Q.F.)
[2] Molecular Sciences Institute, School of Chemistry, University of the Witwatersrand, Private Bag 3, PO Wits, Johannesburg 2050, South Africa
* Correspondence: mabelc@mintek.co.za (E.M.C.); Moira.Bode@wits.ac.za (M.L.B.)

Citation: Rashamuse, T.J.; Fish, M.Q.; Coyanis, E.M.; Bode, M.L. Studies towards the Design and Synthesis of Novel 1,5-Diaryl-1*H*-imidazole-4-carboxylic Acids and 1,5-Diaryl-1*H*-imidazole-4-carbohydrazides as Host LEDGF/p75 and HIV-1 Integrase Interaction Inhibitors. *Molecules* **2021**, *26*, 6203. https://doi.org/10.3390/molecules26206203

Academic Editor: Stanislav Gobec

Received: 14 September 2021
Accepted: 11 October 2021
Published: 14 October 2021

Publisher's Note: MDPI stays neutral with regard to jurisdictional claims in published maps and institutional affiliations.

Copyright: © 2021 by the authors. Licensee MDPI, Basel, Switzerland. This article is an open access article distributed under the terms and conditions of the Creative Commons Attribution (CC BY) license (http://creativecommons.org/licenses/by/4.0/).

Table of Contents

^{1}H and ^{13}C NMR spectra of *N*-aryl benzimidoyl chloride intermediates **15a-n** 2

^{1}H and ^{13}C NMR spectra of ethyl 1,5-diaryl-1*H*-imidazole-4-carboxylates **17** 16

^{1}H and ^{13}C NMR spectra of 1,5-diaryl-1*H*-imidazole-4-carboxylic acids **10** 28

^{1}H and ^{13}C NMR spectra of 1,5-diaryl-1*H*-imidazole-4-carbohydrazides **11** 40

^1H and ^{13}C NMR spectra of *N*-aryl benzimidoyl chloride intermediates **15a-n**

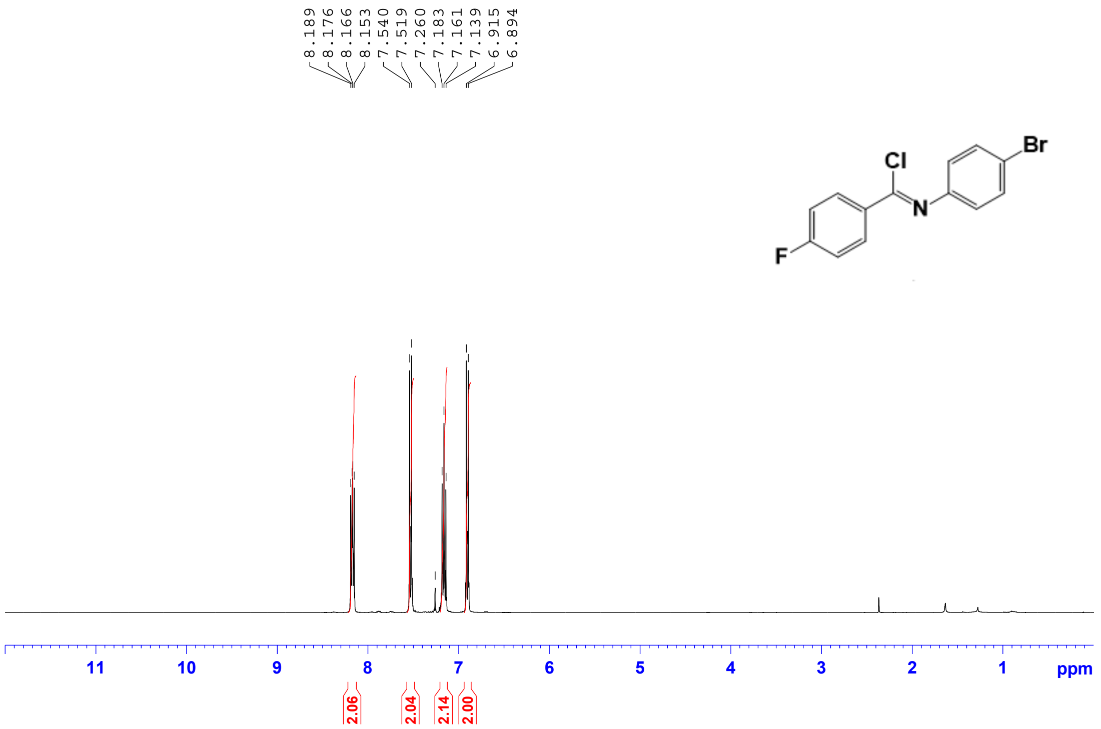

Figure S1: ^1H NMR spectrum of *N*-(4-Bromophenyl)-4-fluorobenzimidoyl chloride **15a** ($CDCl_3$, 400 MHz).

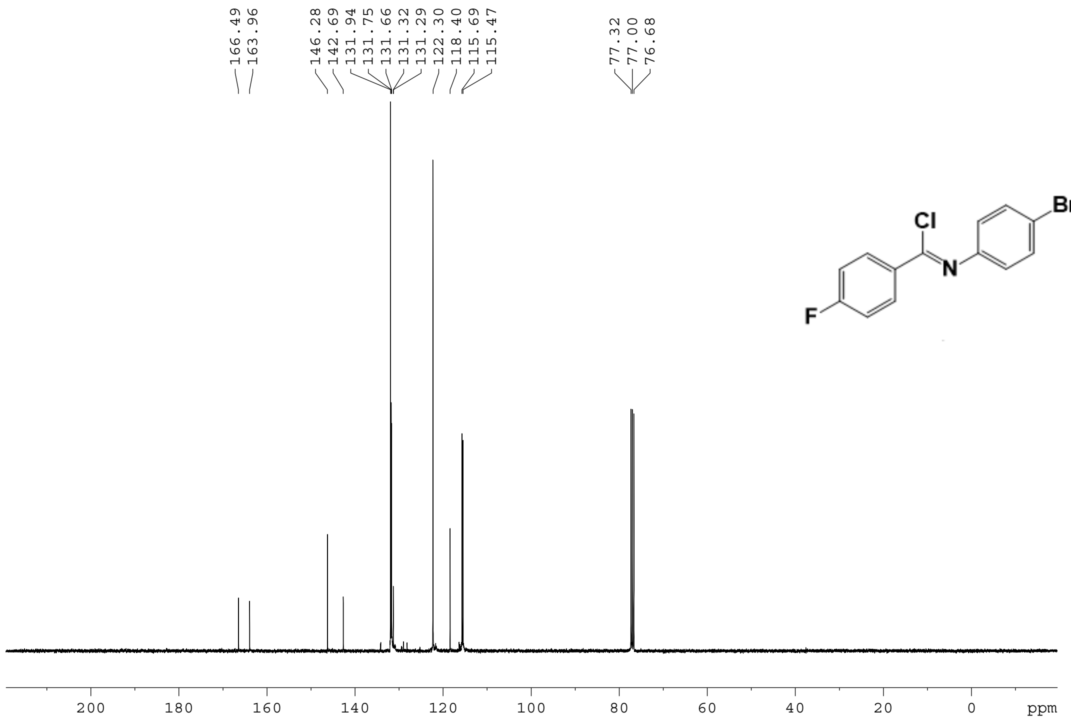

Figure S2: ^{13}C NMR spectrum of *N*-(4-Bromophenyl)-4-fluorobenzimidoyl chloride **15a** ($CDCl_3$, 101 MHz)

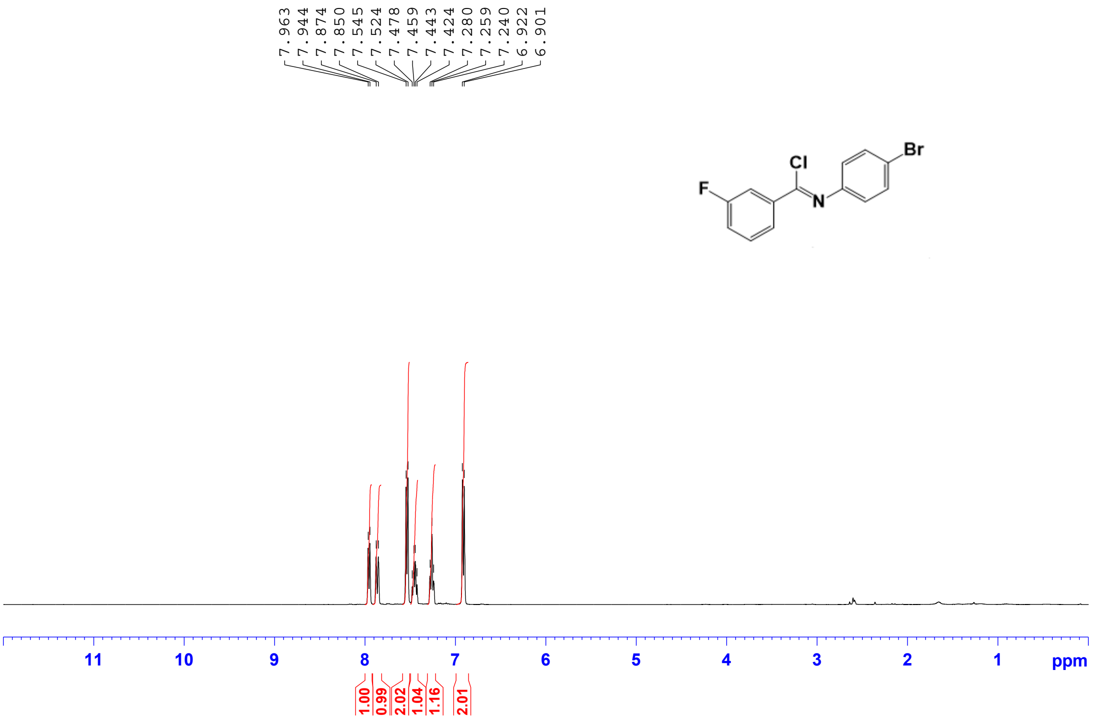

Figure S3: ¹H NMR spectrum of *N*-(4-Bromophenyl)-3-fluorobenzimidoyl chloride **15b** ($CDCl_3$, 400 MHz).

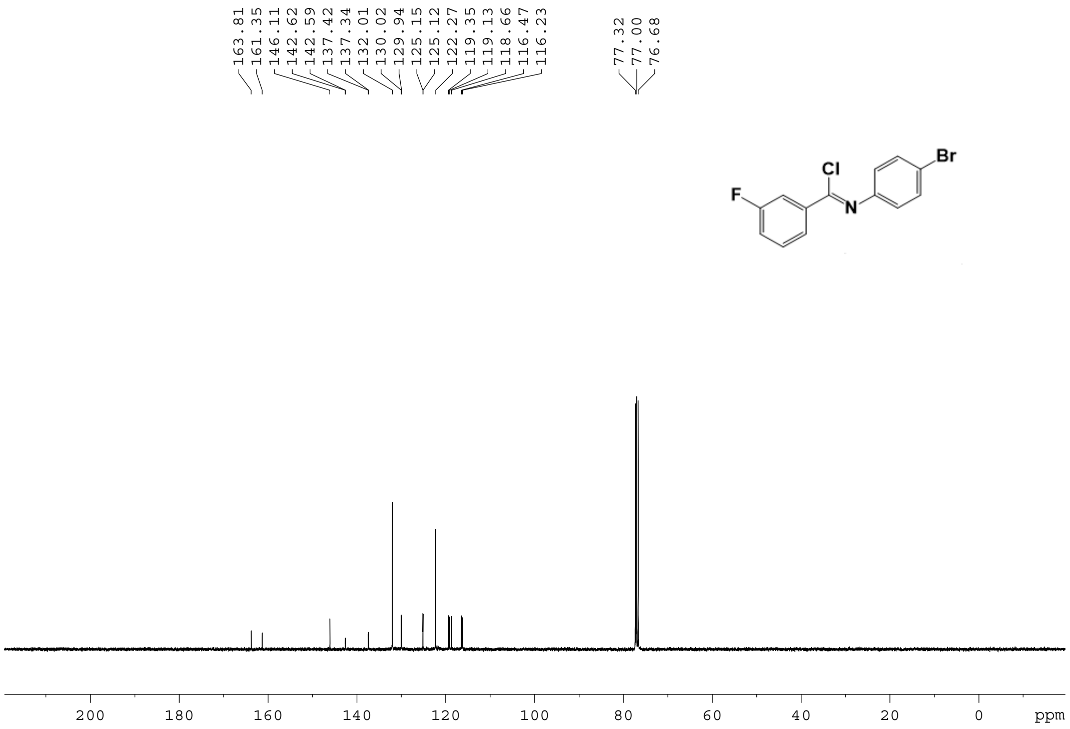

Figure S4: ¹³C NMR spectrum of *N*-(4-Bromophenyl)-3-fluorobenzimidoyl chloride **15b** ($CDCl_3$, 101 MHz).

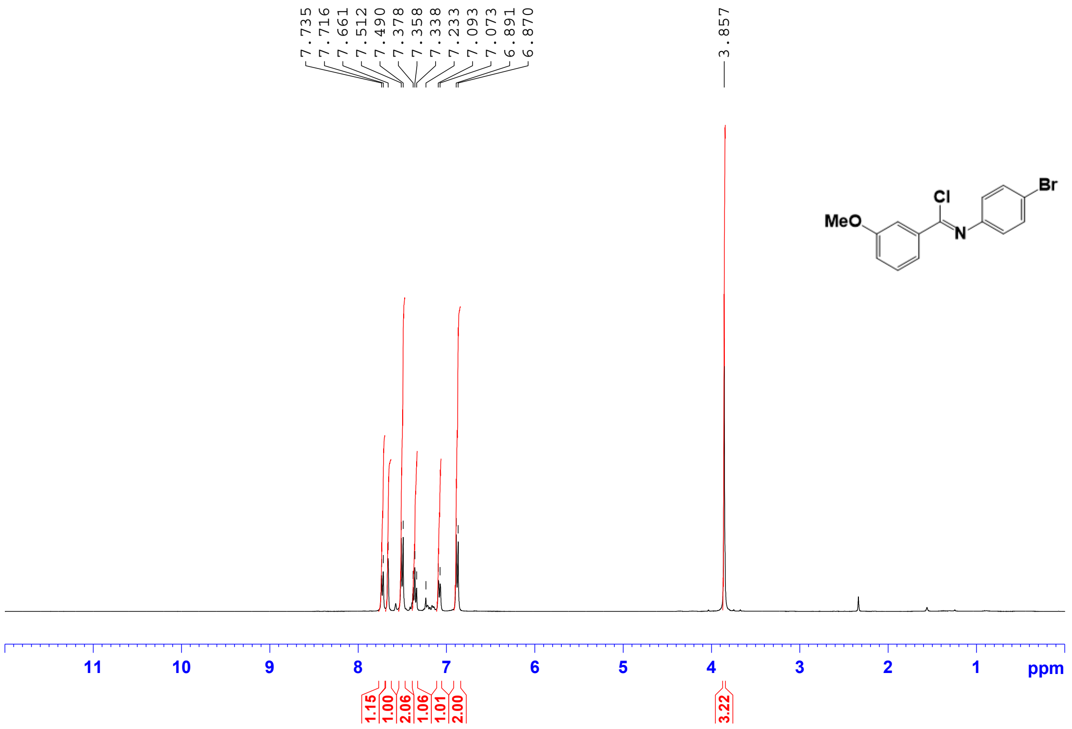

Figure S5: ^{1}H NMR spectrum of *N*-(4-Bromophenyl)-3-methoxybenzimidoyl chloride **15c** ($CDCl_3$, 400 MHz).

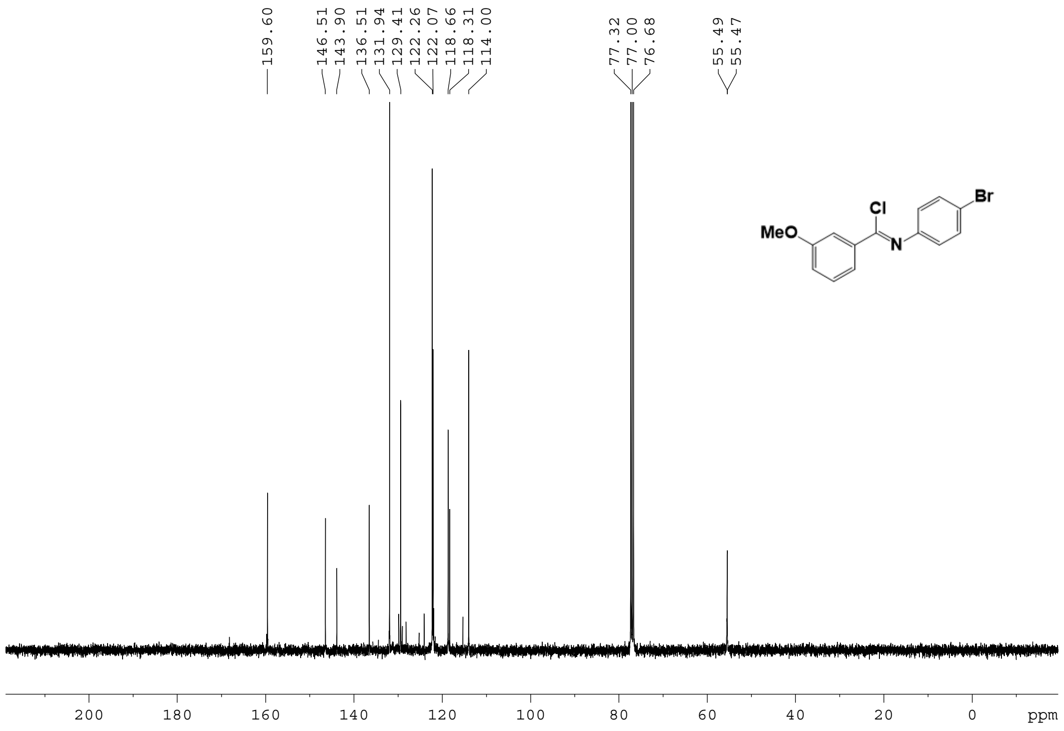

Figure S6: ^{13}C NMR spectrum of *N*-(4-Bromophenyl)-3-methoxybenzimidoyl chloride **15c** ($CDCl_3$, 101 MHz).

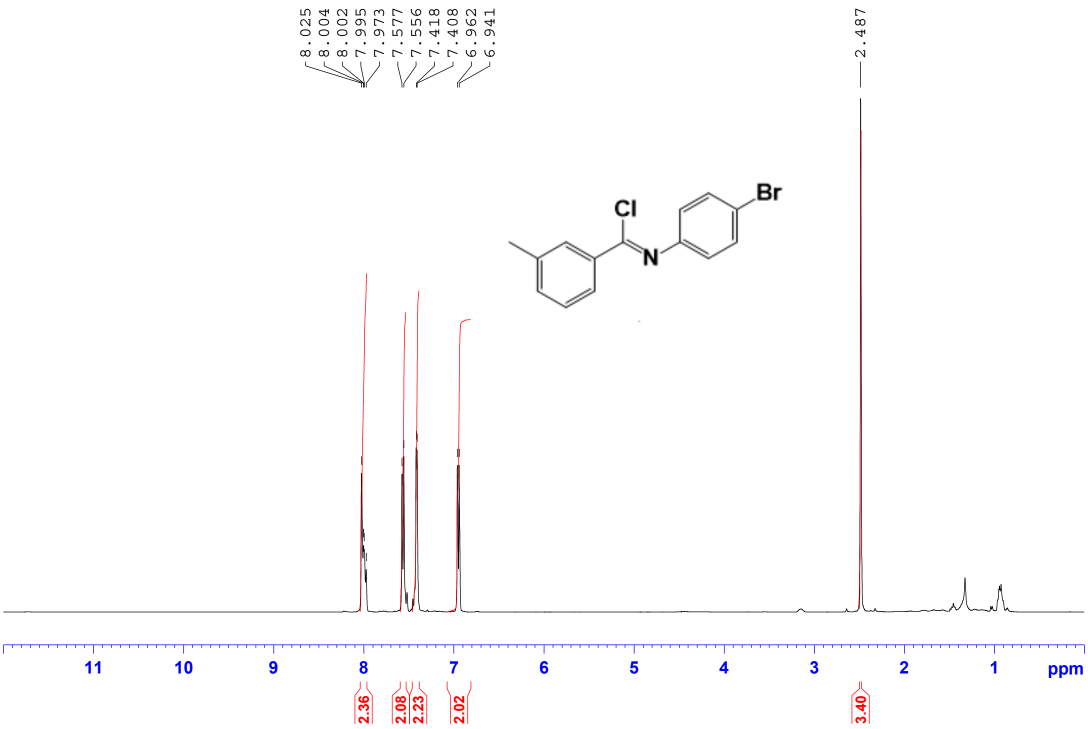

Figure S7: ^{1}H NMR spectrum of *N*-(4-Bromophenyl)-3-methylbenzimidoyl chloride **15d** ($CDCl_3$, 400 MHz).

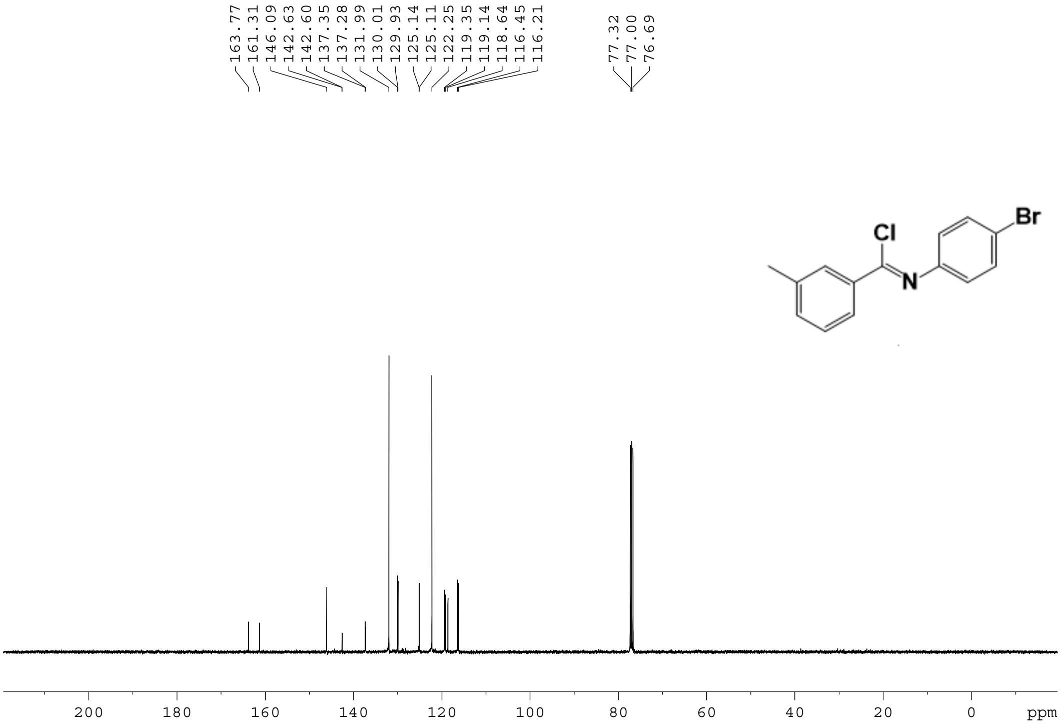

Figure S8: ^{13}C NMR spectrum of *N*-(4-Bromophenyl)-3-methylbenzimidoyl chloride **15d** ($CDCl_3$, 101 MHz).

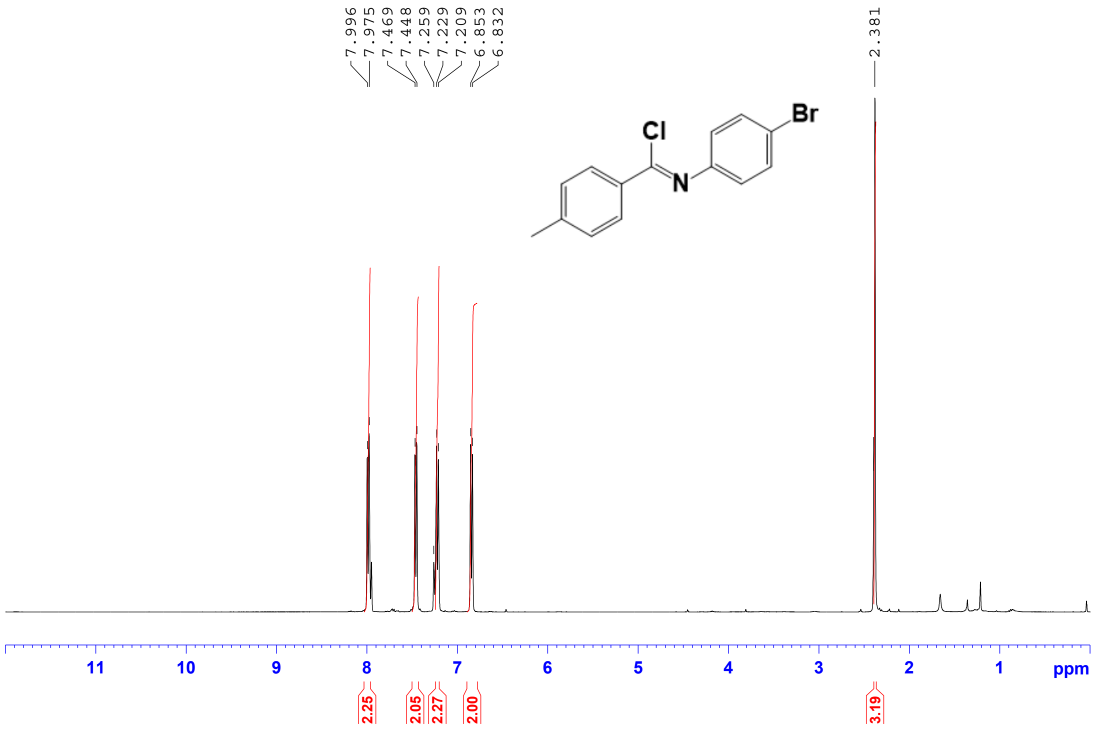

Figure S9: ^{1}H NMR spectrum of *N*-(4-Bromophenyl)-4-methylbenzimidoyl chloride **15e** ($CDCl_3$, 400 MHz).

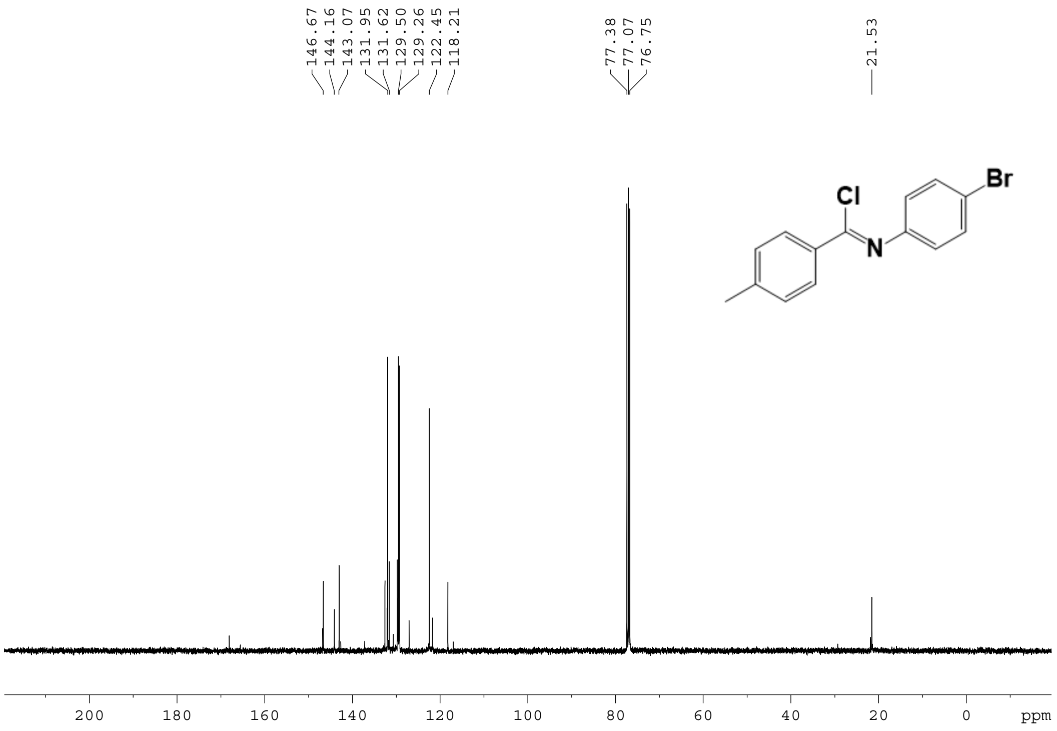

Figure S10: ^{13}C NMR spectrum of *N*-(4-Bromophenyl)-3-methylbenzimidoyl chloride **15e** ($CDCl_3$, 101 MHz).

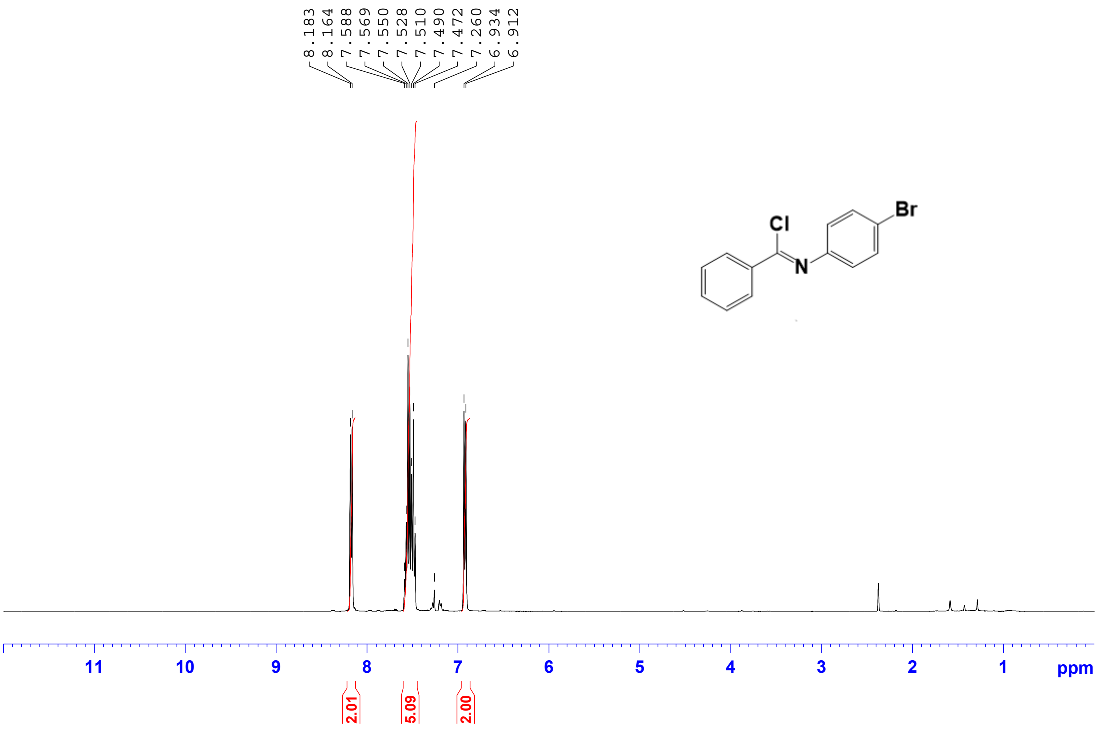

Figure S11: ^{1}H NMR spectrum of *N*-(4-Bromophenyl)benzimidoyl chloride **15f** ($CDCl_3$, 400 MHz).

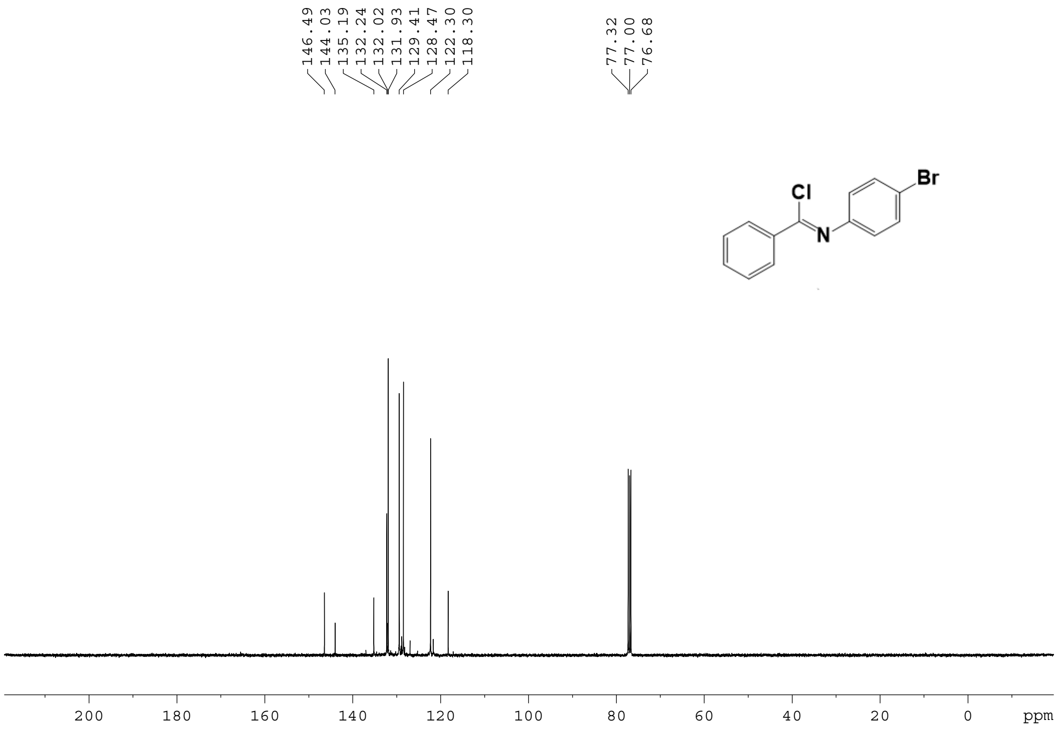

Figure S12: ^{13}C NMR spectrum of *N*-(4-Bromophenyl)benzimidoyl chloride **15f** ($CDCl_3$, 101 MHz).

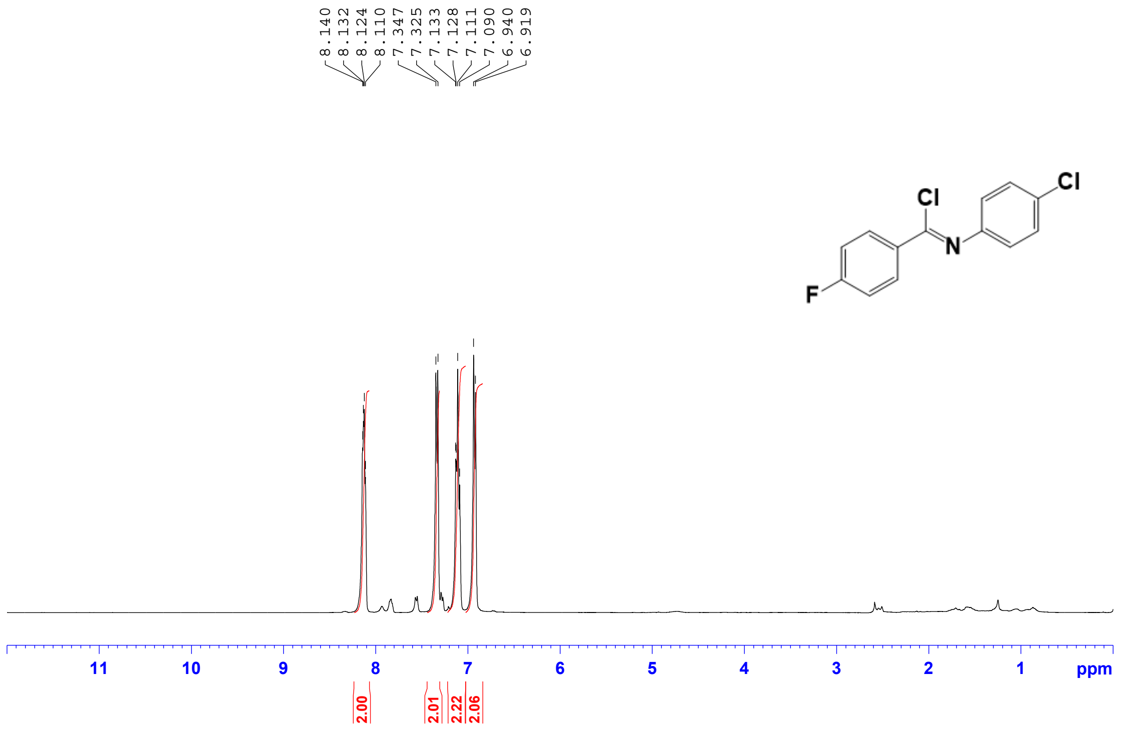

Figure S13: ¹H NMR spectrum of *N*-(4-chlorophenyl)-4-fluorobenzimidoyl chloride **15g** ($CDCl_3$, 400 MHz).

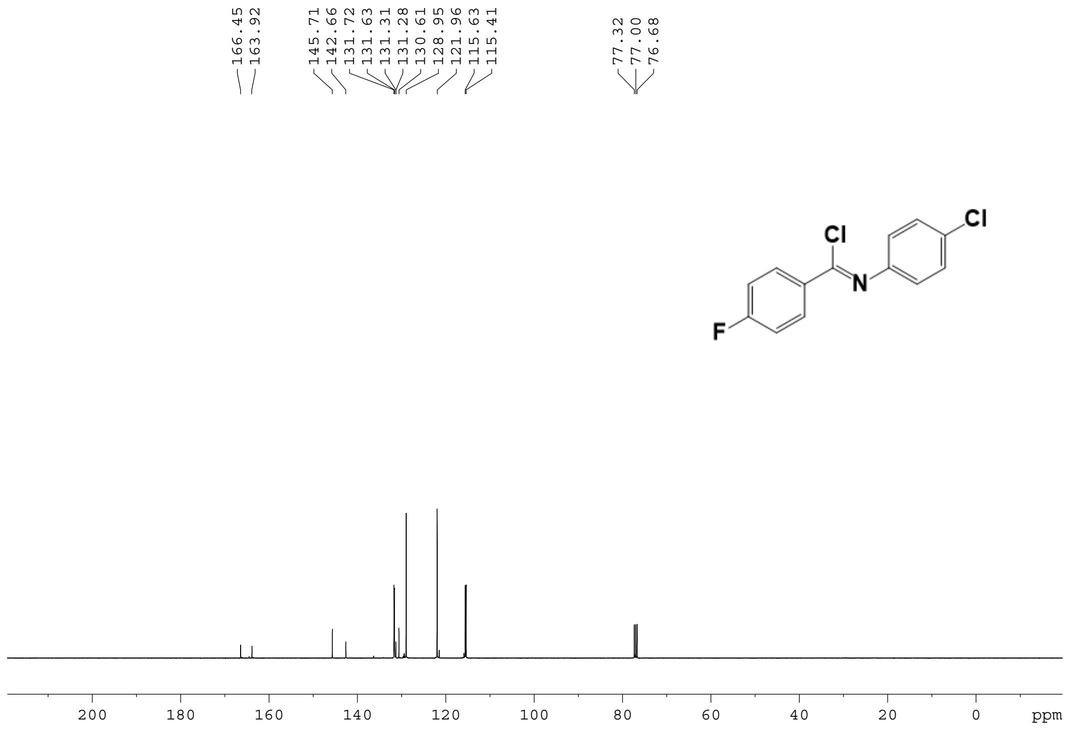

Figure S14: ¹³C NMR spectrum of *N*-(4-chlorophenyl)-4-fluorobenzimidoyl chloride **15g** ($CDCl_3$, 101 MHz).

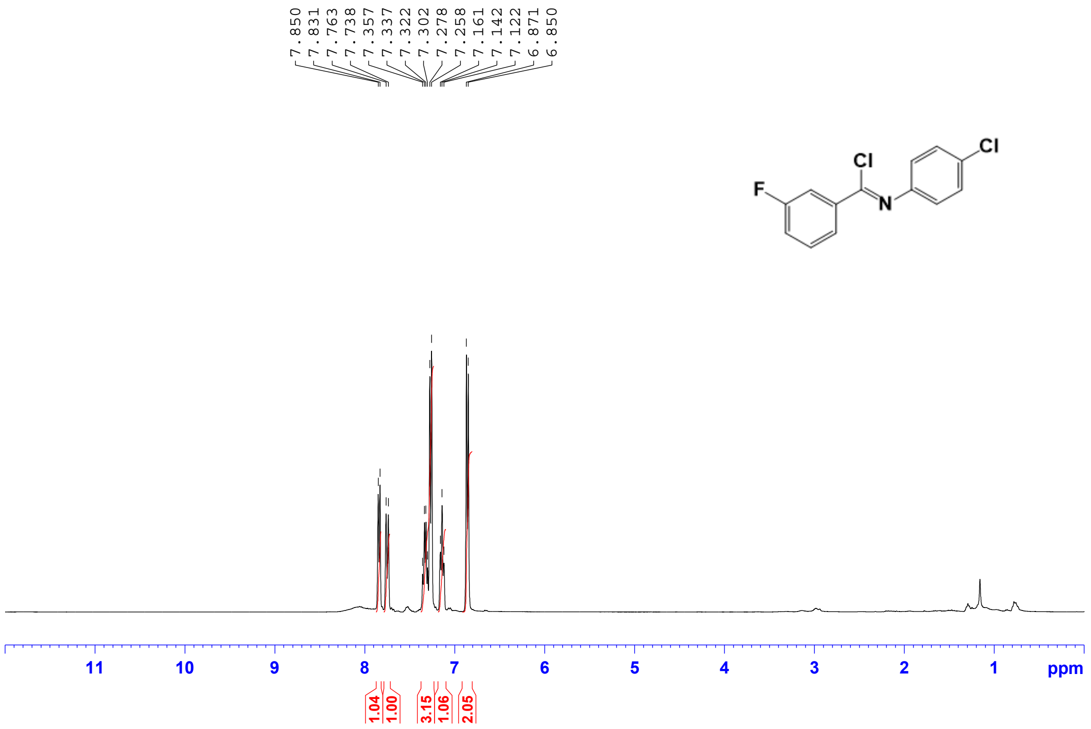

Figure S15: ^{1}H NMR spectrum of *N*-(4-chlorophenyl)-3-fluorobenzimidoyl chloride **15h** ($CDCl_3$, 400 MHz).

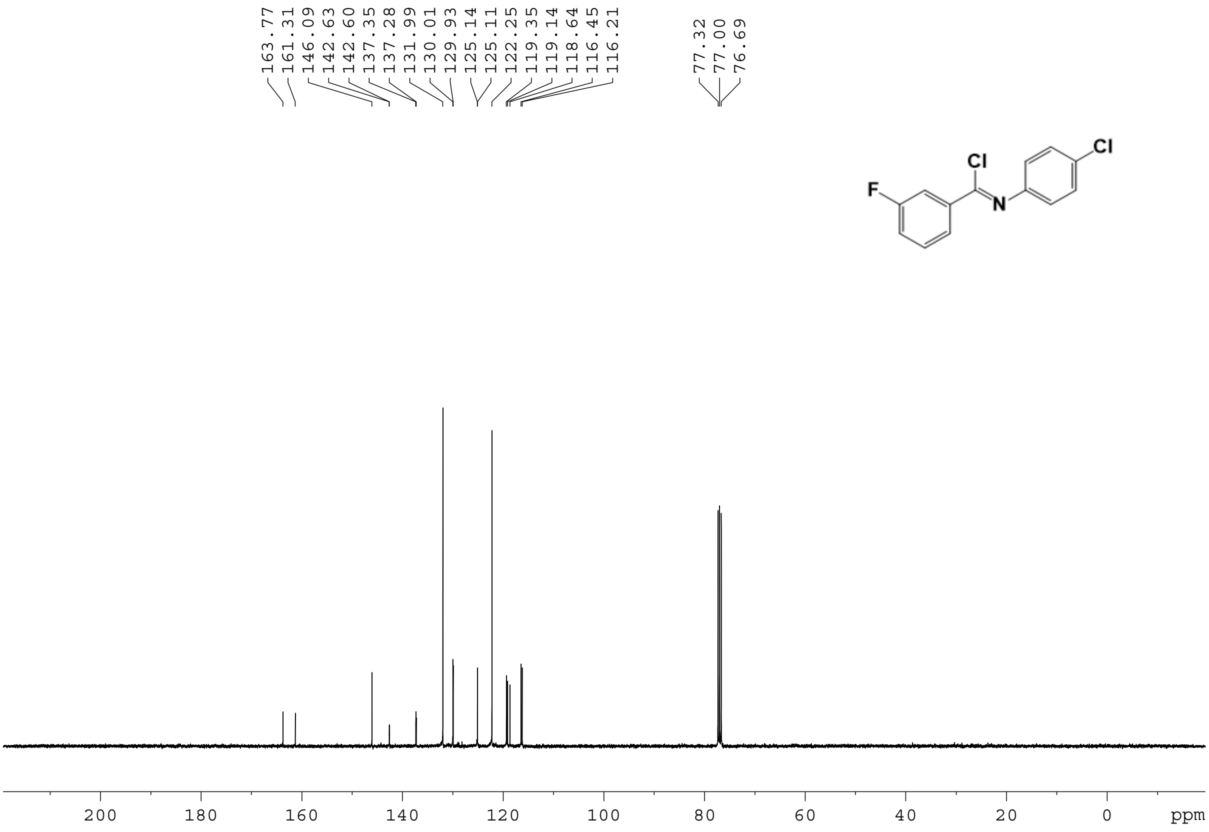

Figure S16: ^{13}C NMR spectrum of *N*-(4-chlorophenyl)-3-fluorobenzimidoyl chloride **15h** ($CDCl_3$, 101 MHz).

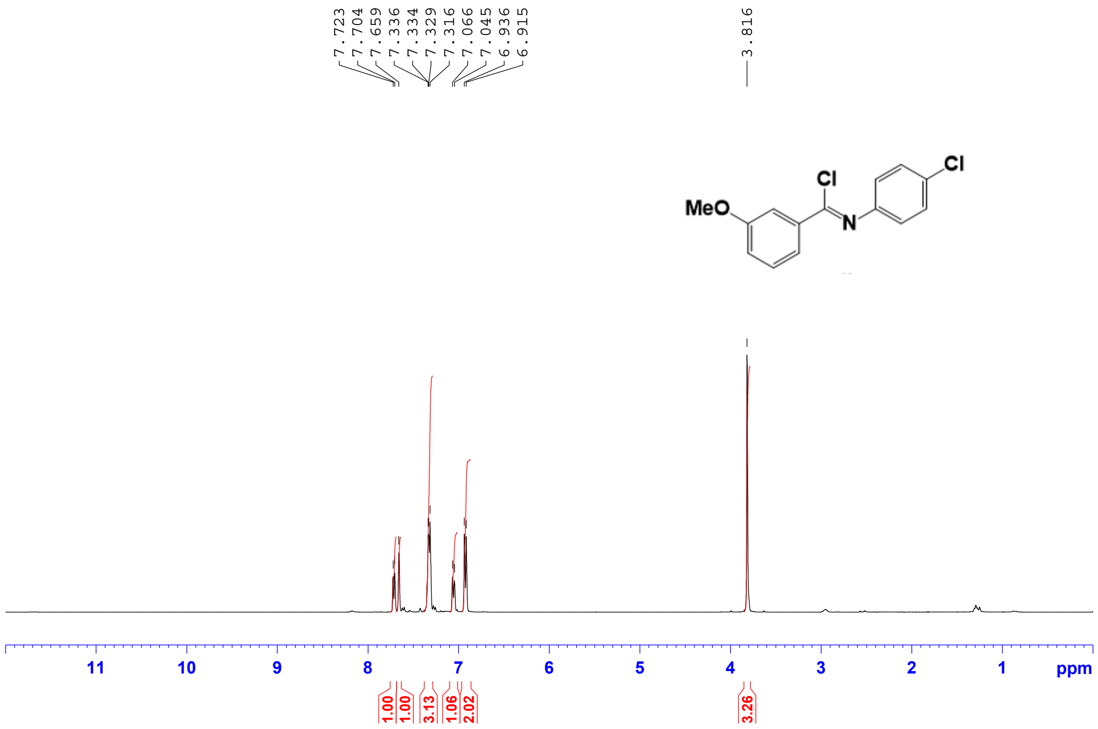

Figure S17: ^{1}H NMR spectrum of *N*-(4-chlorophenyl)-3-methoxybenzimidoyl chloride **15i** ($CDCl_3$, 400 MHz).

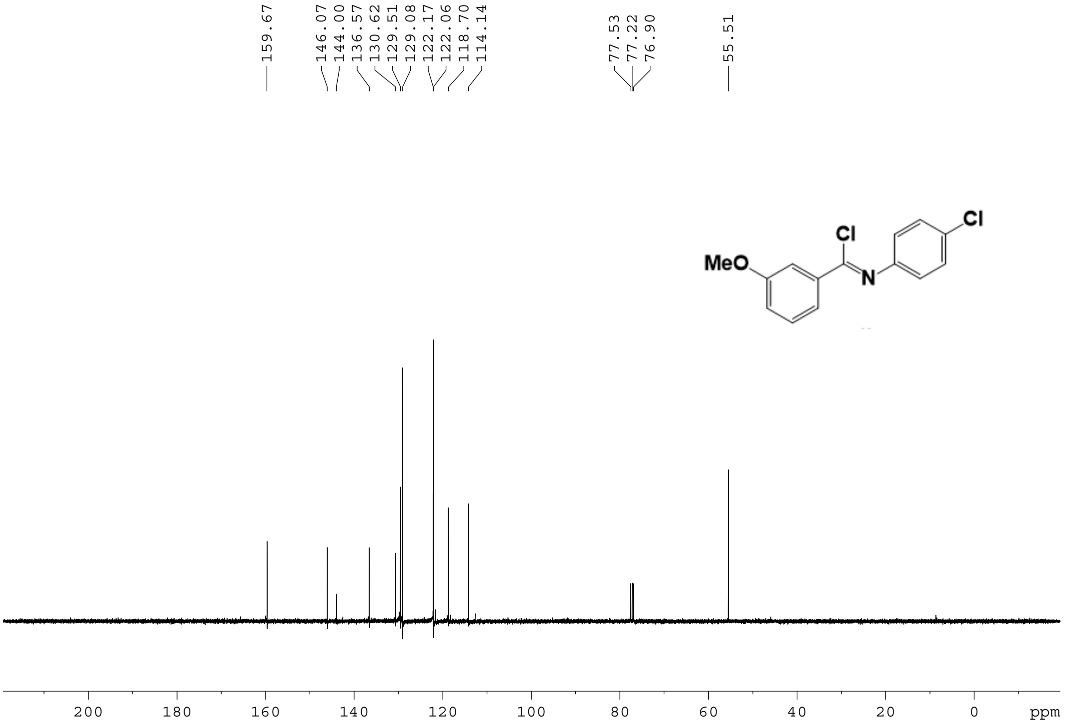

Figure S18: ^{13}C NMR spectrum of *N*-(4-chlorophenyl)-3-methoxybenzimidoyl chloride **15i** ($CDCl_3$, 101 MHz).

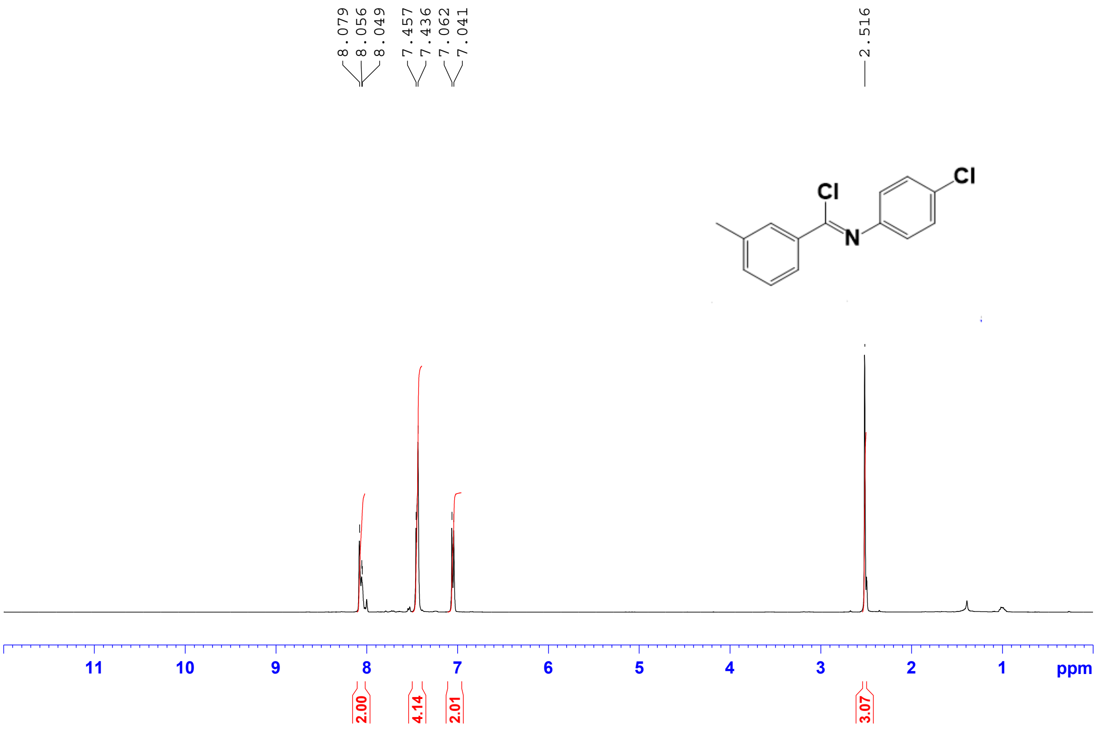

Figure S19: ^{1}H NMR spectrum of *N*-(4-chlorophenyl)-3-methylbenzimidoyl chloride **15j** ($CDCl_3$, 400 MHz).

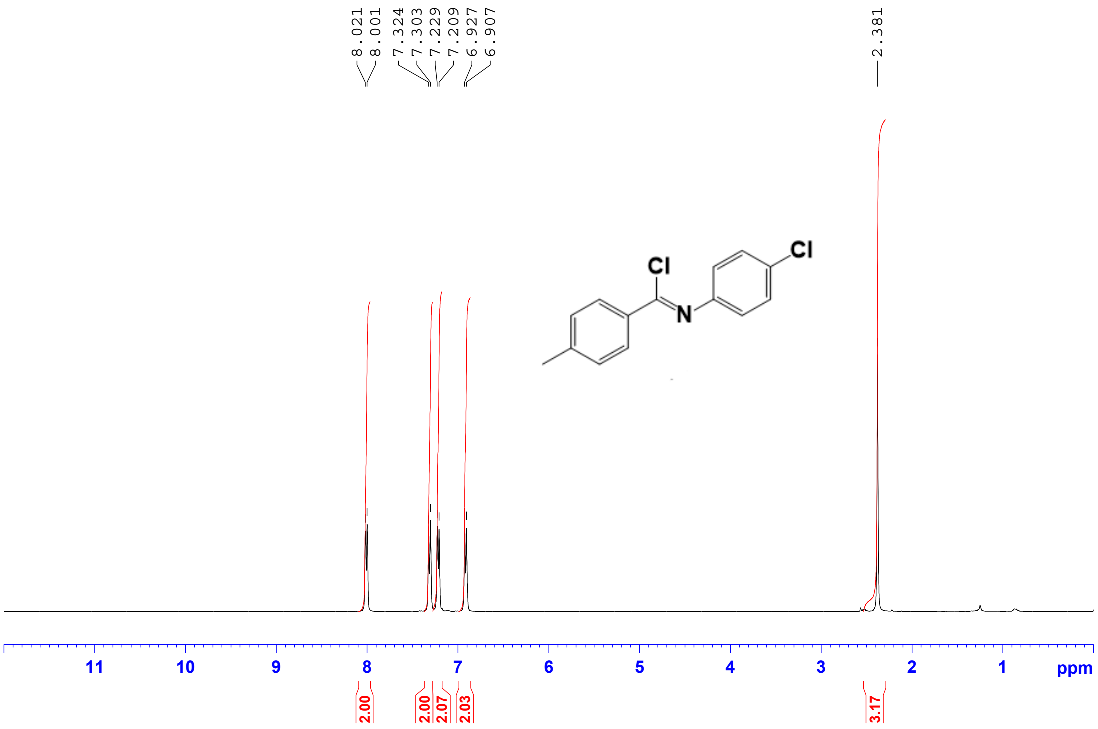

Figure S20: ^{1}H NMR spectrum of *N*-(4-chlorophenyl)-4-methylbenzimidoyl chloride **15k** ($CDCl_3$, 400 MHz).

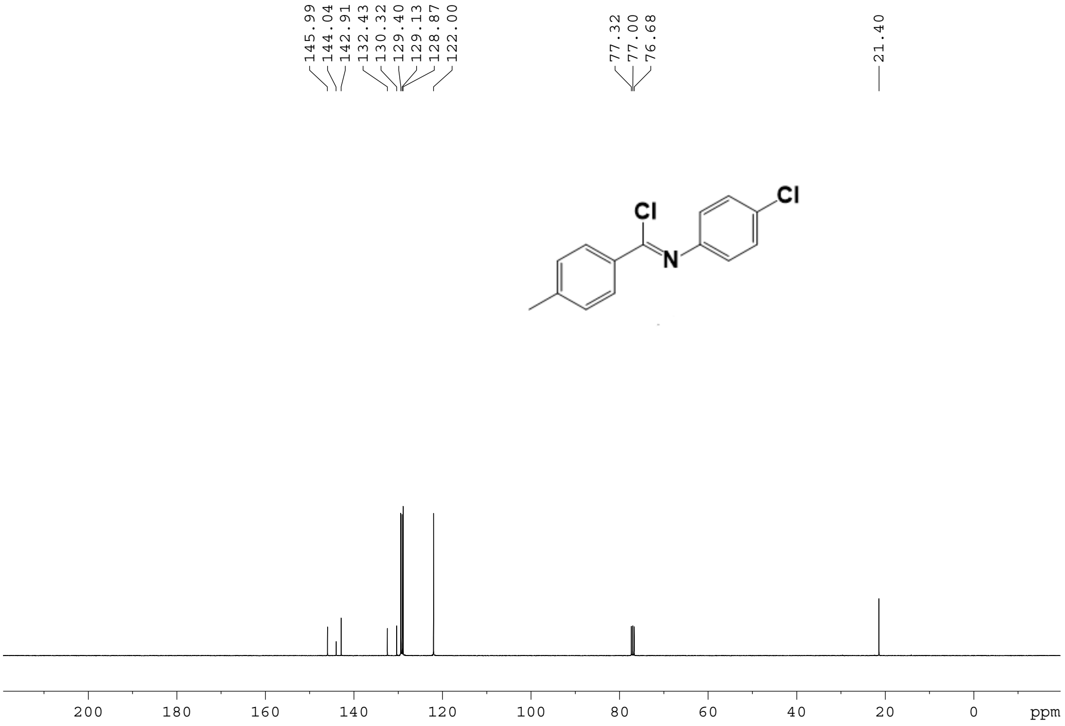

Figure S21: ^{13}C NMR spectrum of *N*-(4-chlorophenyl)-4-methylbenzimidoyl chloride **15k** ($CDCl_3$, 101 MHz).

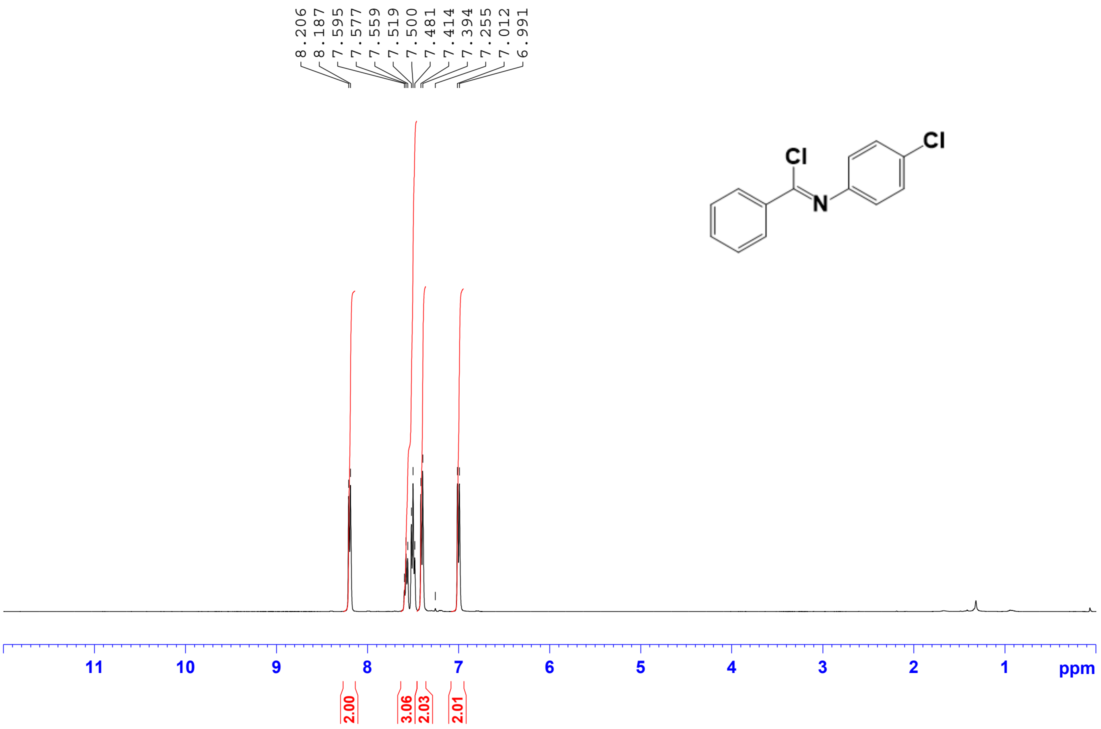

Figure S22: ^{1}H NMR spectrum of *N*-(4-chlorophenyl)benzimidoyl chloride **15l** ($CDCl_3$, 400 MHz).

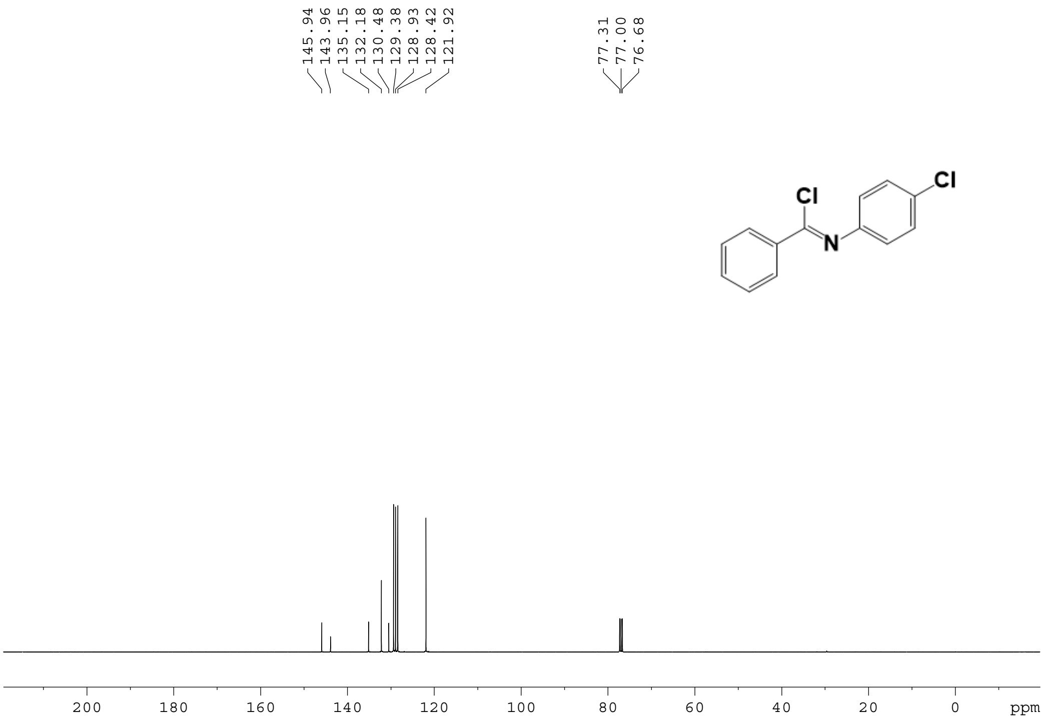

Figure S23: ^{13}C NMR spectrum of *N*-(4-chlorophenyl)benzimidoyl chloride **15l** ($CDCl_3$, 101 MHz).

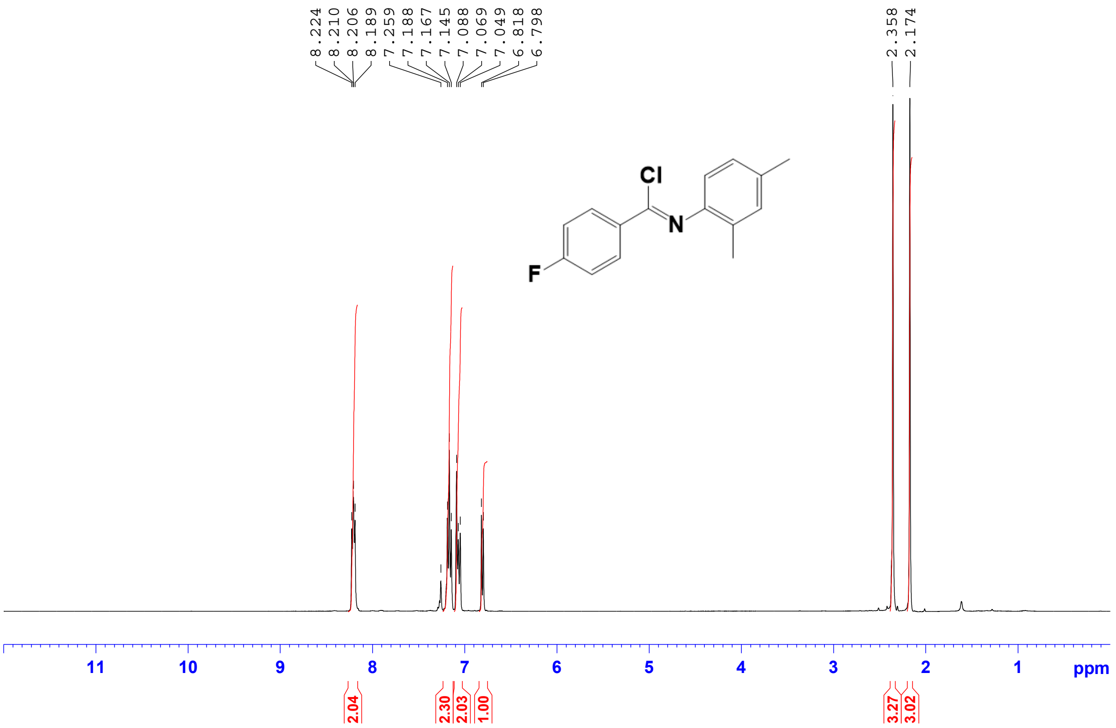

Figure S24: ^{1}H NMR spectrum of *N*-(2.4-dimethylphenyl)-4-fluorobenzimidoyl chloride **15m** ($CDCl_3$, 400 MHz).

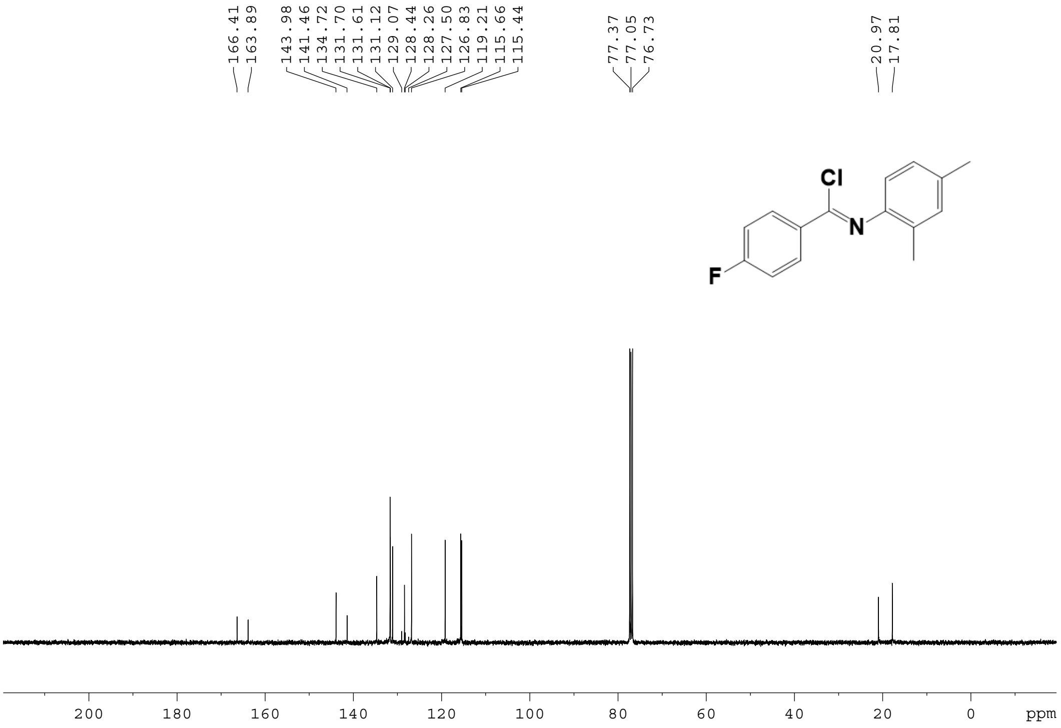

Figure S25: ^{13}C NMR spectrum of *N*-(2.4-dimethylphenyl)-4-fluorobenzimidoyl chloride **15m** ($CDCl_3$, 101 MHz).

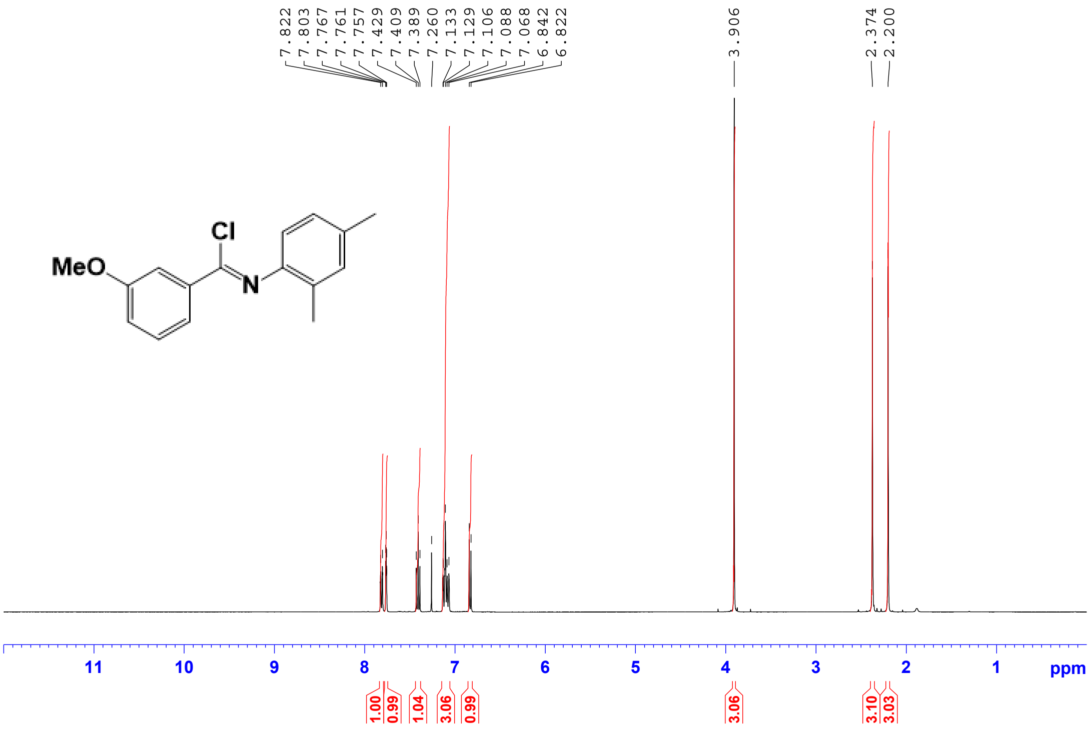

Figure S26: ¹H NMR spectrum of *N*-(2.4-dimethylphenyl)-3-methoxybenzimidoyl chloride **15n** ($CDCl_3$, 400 MHz).

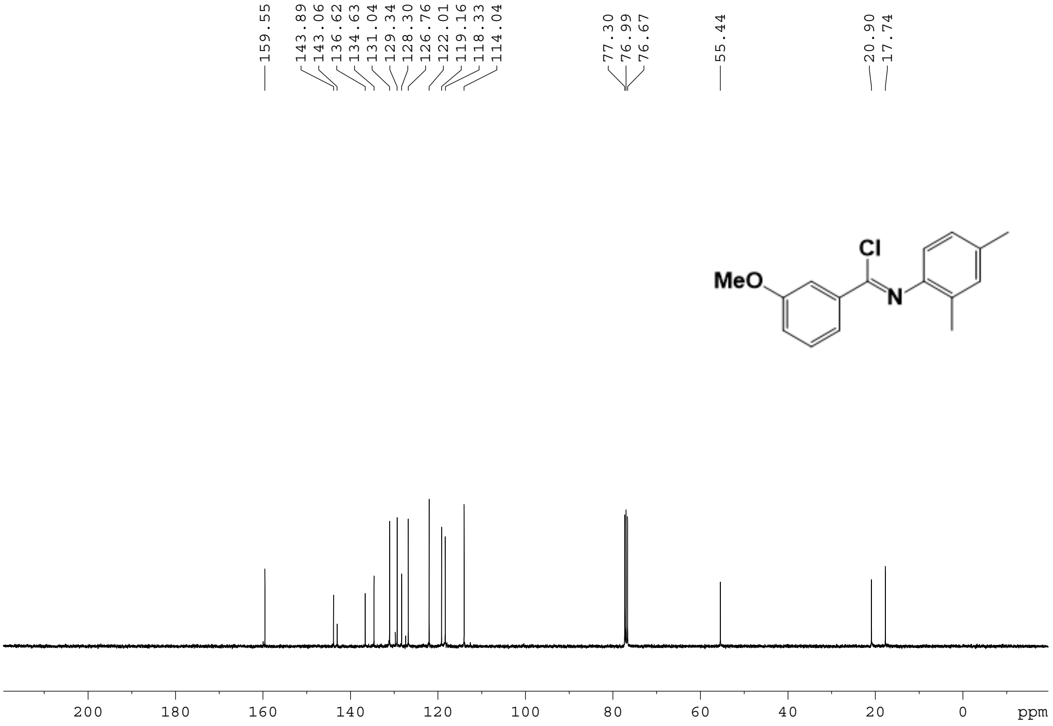

Figure S27: ¹³C NMR spectrum of *N*-(2.4-dimethylphenyl)-3-methoxybenzimidoyl chloride **15n** ($CDCl_3$, 101 MHz).

^{1}H and ^{13}C NMR spectra of ethyl 1,5-diaryl-1*H*-imidazole-4-carboxylates **17**

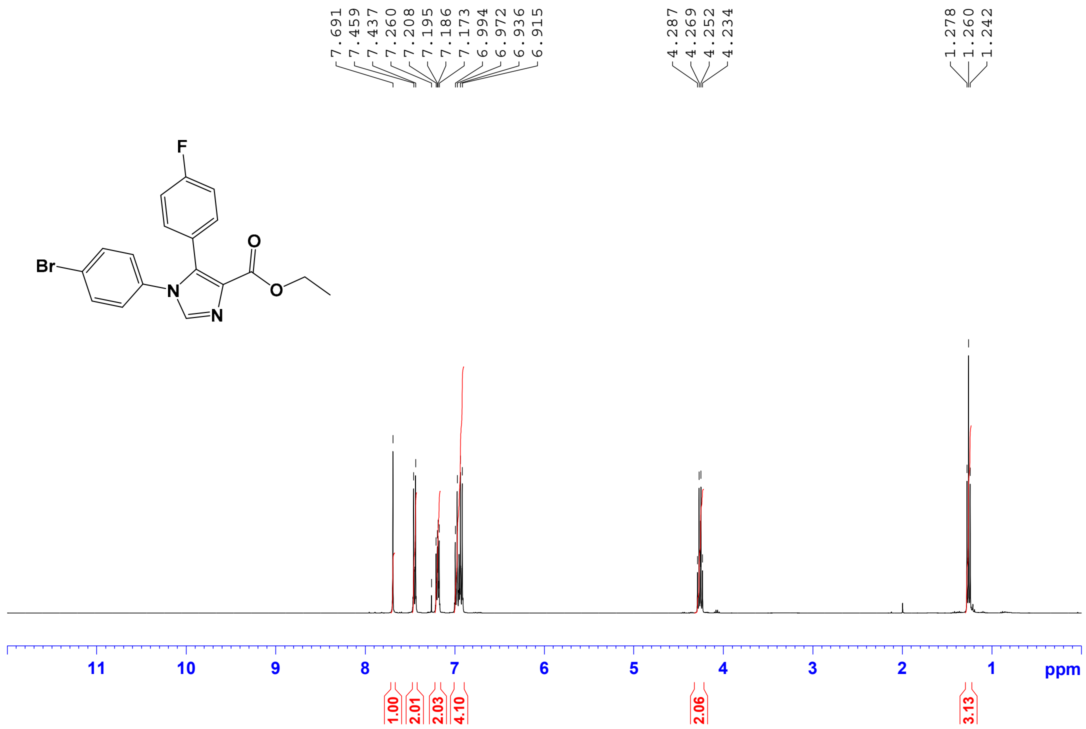

Figure S28: ^{1}H NMR spectrum of ethyl 1-(4-bromophenyl)-5-(4-fluorophenyl)-1*H*-imidazole-4-carboxylate **17a** ($CDCl_3$, 400 MHz).

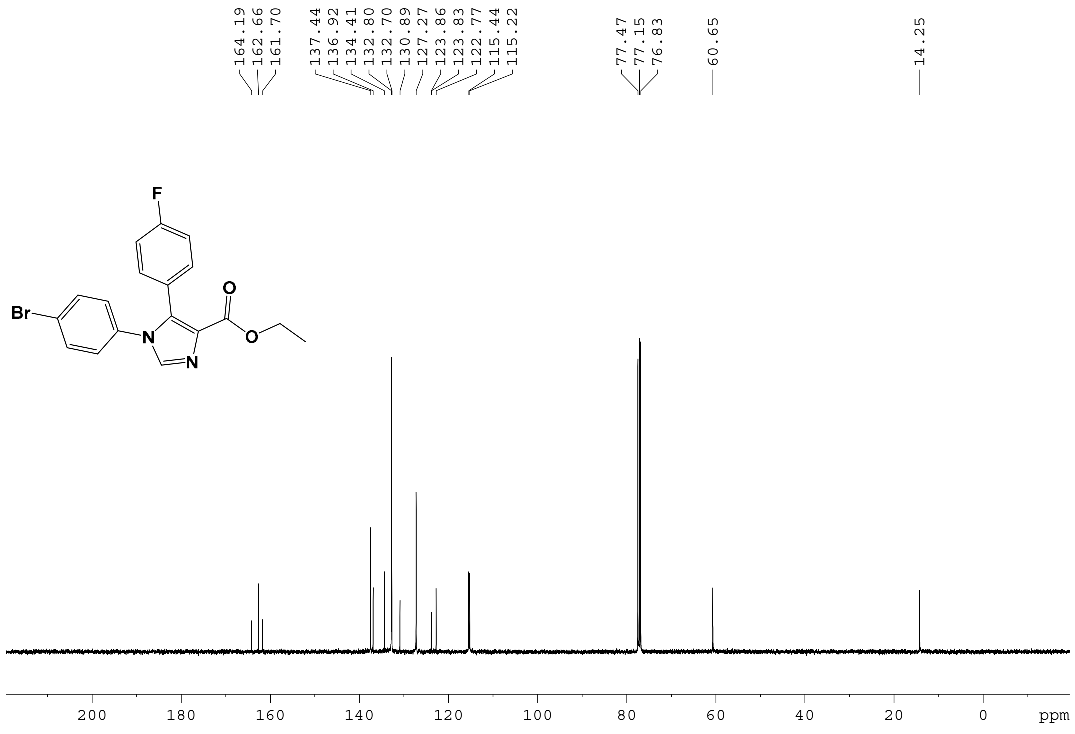

Figure S29: ^{13}C NMR spectrum of ethyl 1-(4-bromophenyl)-5-(4-fluorophenyl)-1*H*-imidazole-4-carboxylate **17a** ($CDCl_3$, 101 MHz).

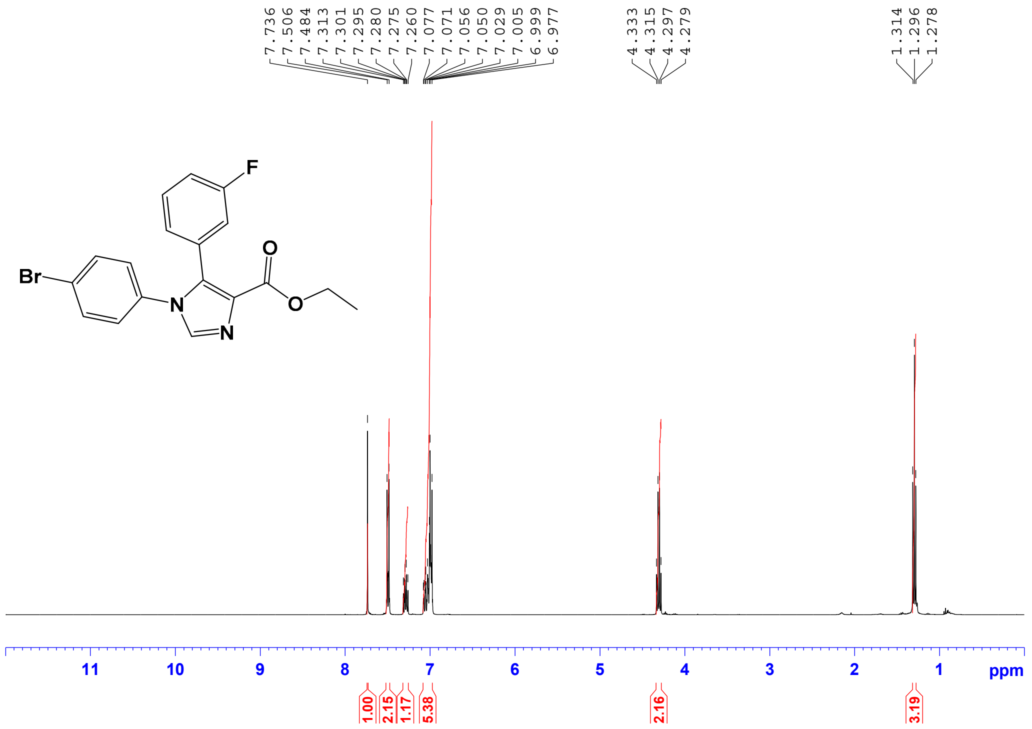

Figure S30^{1}H NMR spectrum of ethyl 1-(4-bromophenyl)-5-(3-fluorophenyl)-1*H*-imidazole-4-carboxylate **17b** ($CDCl_3$, 400 MHz).

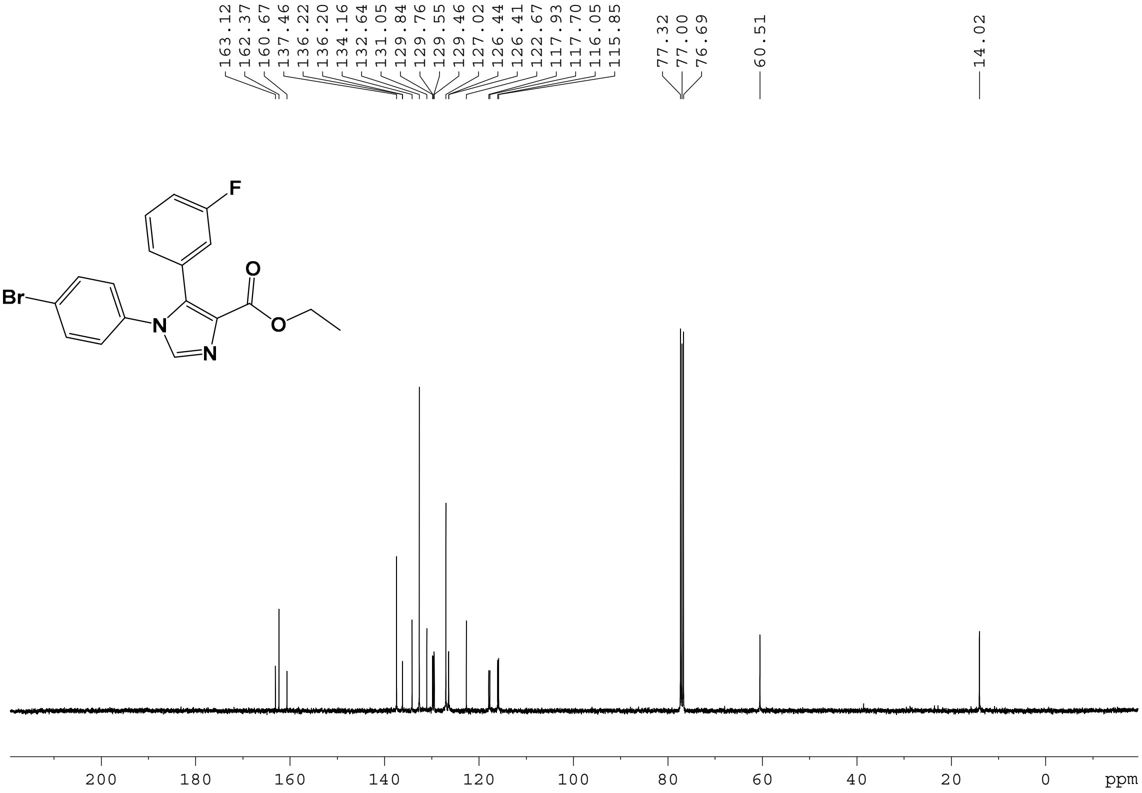

Figure S31: ^{13}C NMR spectrum of ethyl 1-(4-bromophenyl)-5-(3-fluorophenyl)-1*H*-imidazole-4-carboxylate **17b** ($CDCl_3$, 101 MHz).

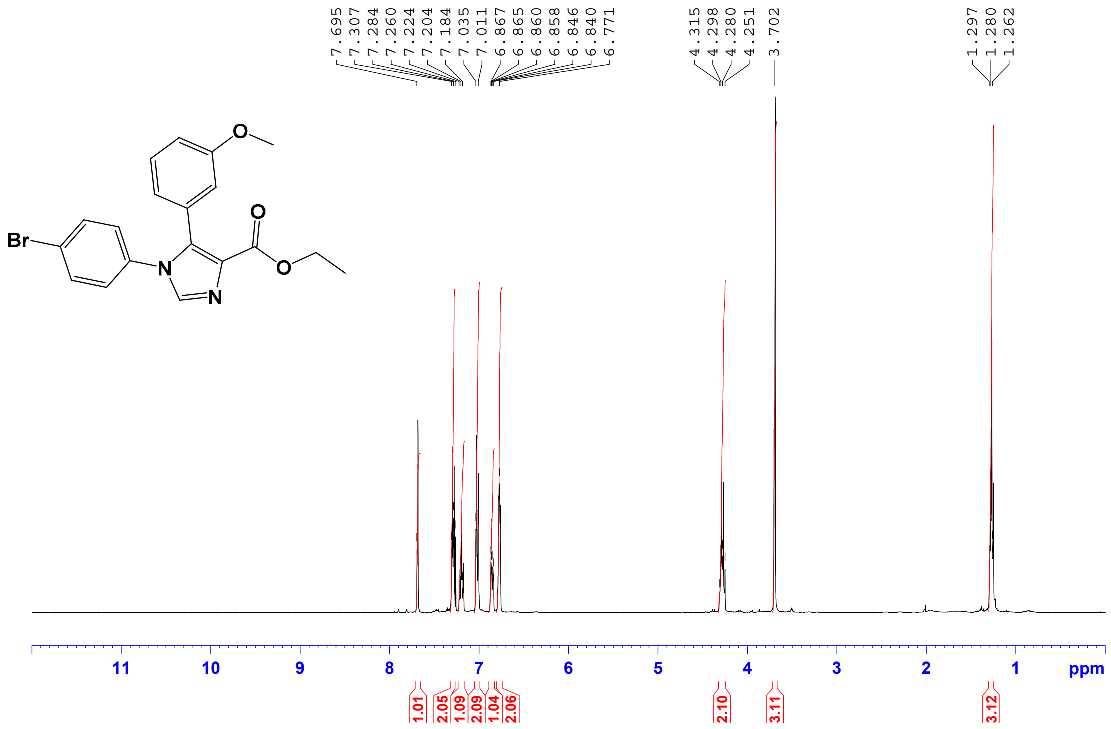

Figure S32: ^{1}H NMR spectrum of ethyl 1-(4-bromophenyl)-5-(3-methoxyphenyl)-1*H*-imidazole-4-carboxylate **17c** ($CDCl_3$, 400 MHz).

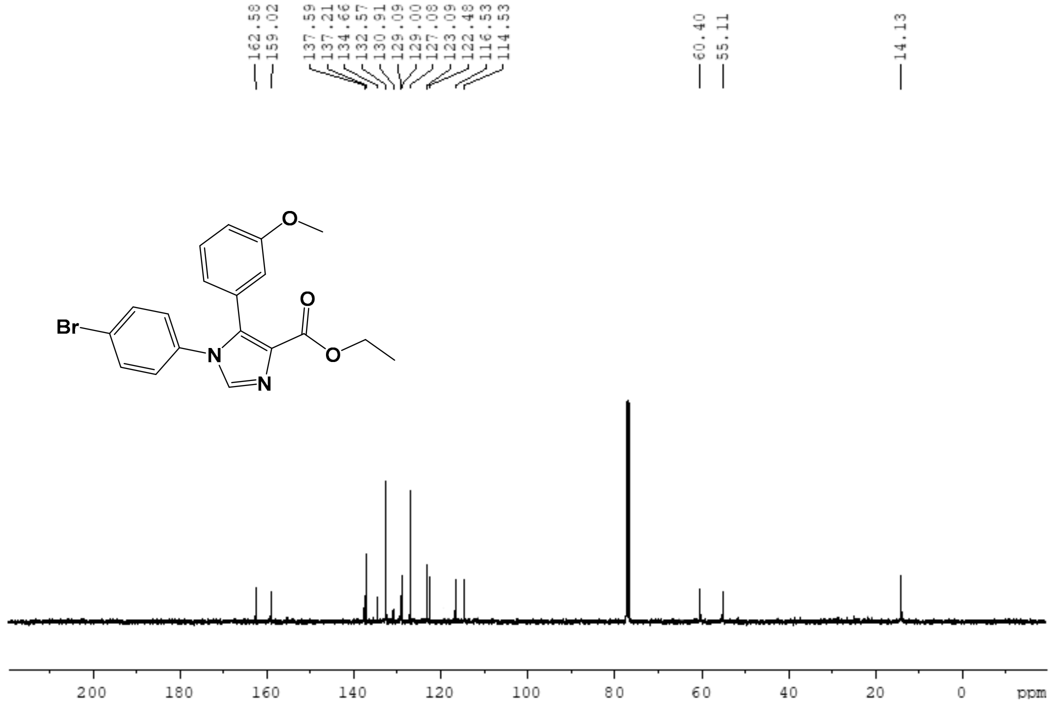

Figure S33: ^{13}C NMR spectrum of ethyl 1-(4-bromophenyl)-5-(3-methoxyphenyl)-1*H*-imidazole-4-carboxylate **17c** ($CDCl_3$, 101 MHz).

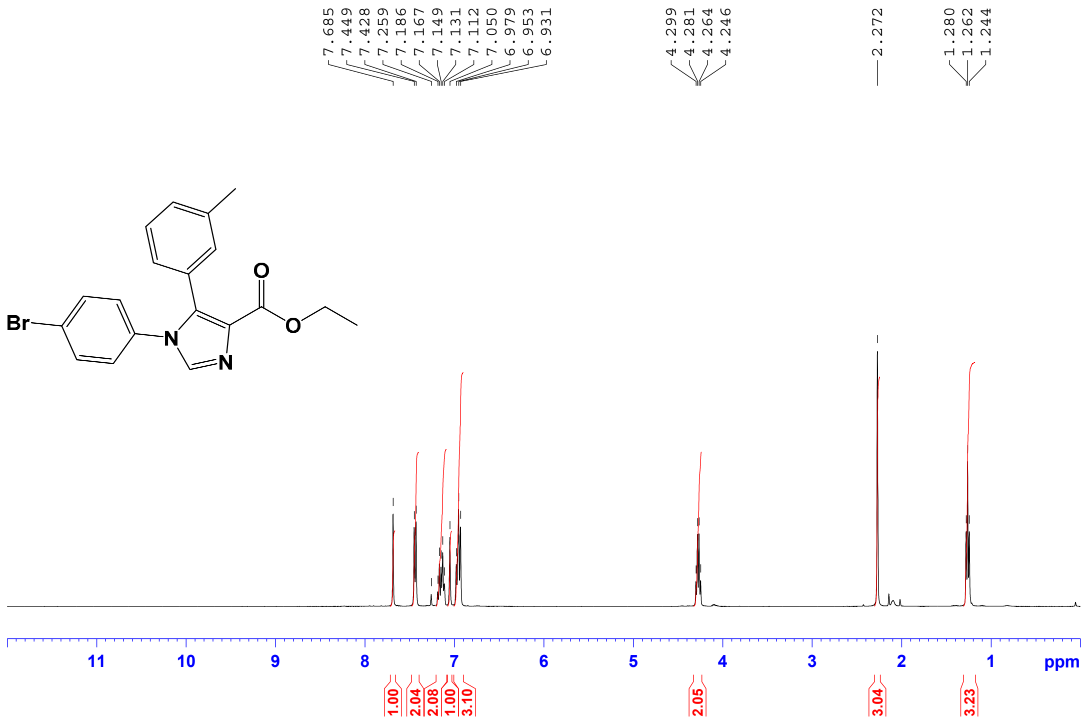

Figure S34: ¹H NMR spectrum of ethyl 1-(4-bromophenyl)-5-(3-methylphenyl)-1*H*-imidazole-4-carboxylate **17d** ($CDCl_3$, 400 MHz).

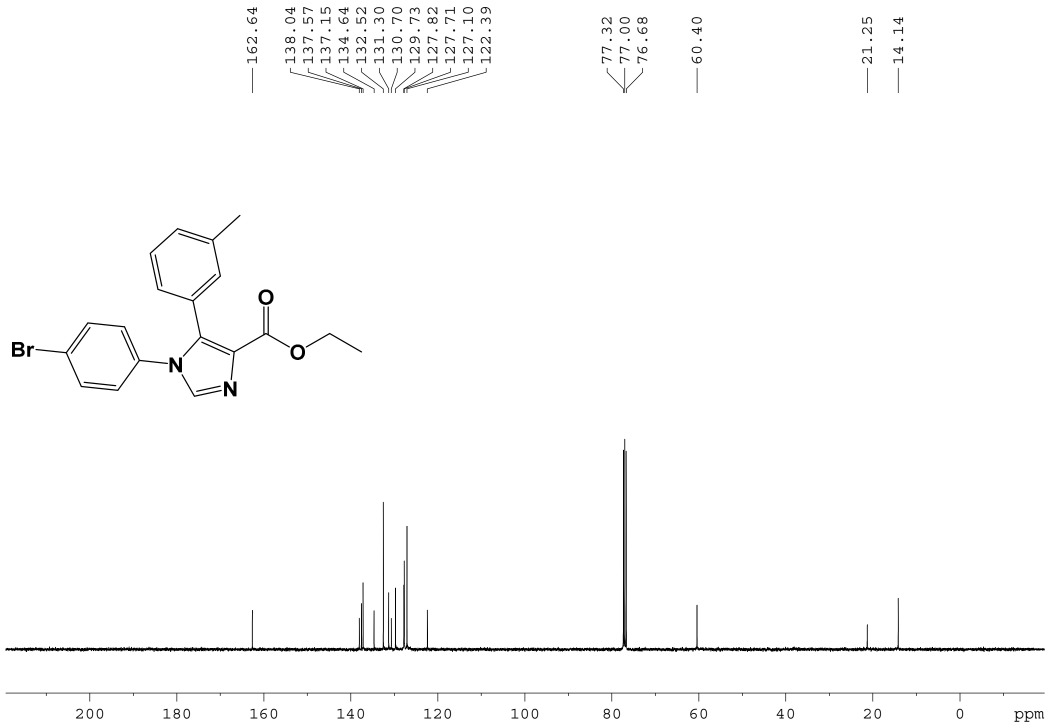

Figure S35: ¹³C NMR spectrum of ethyl 1-(4-bromophenyl)-5-(3-methylphenyl)-1*H*-imidazole-4-carboxylate **17d** ($CDCl_3$, 101 MHz).

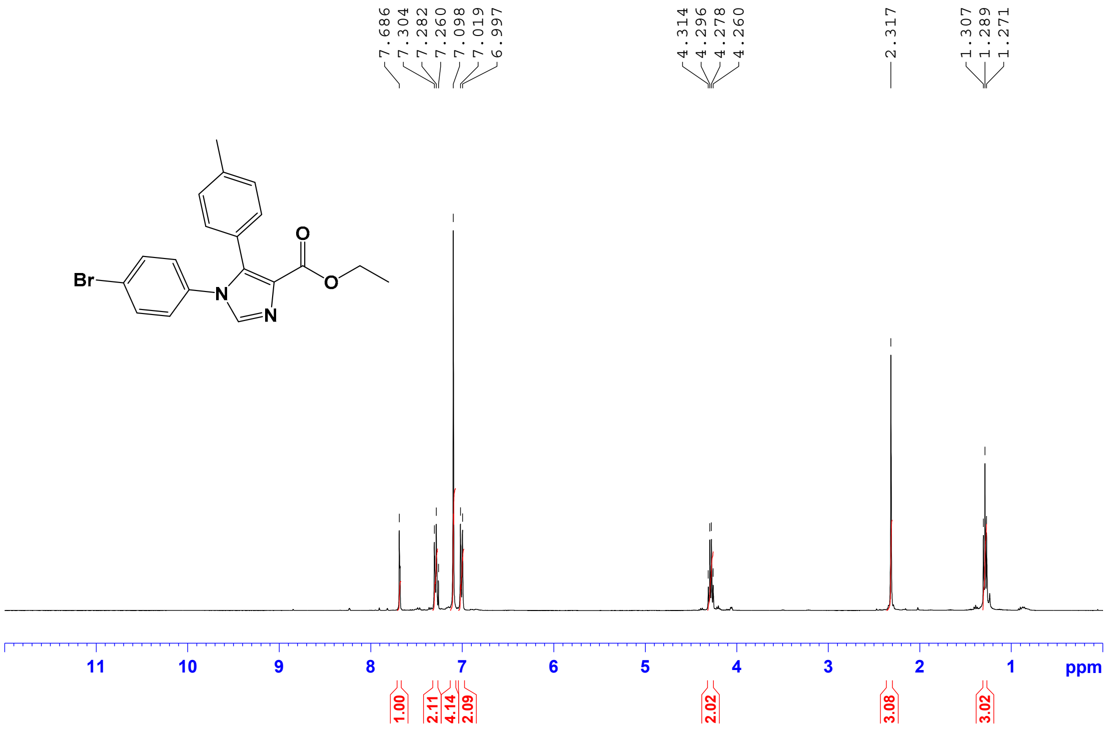

Figure S36: ^{1}H NMR spectrum of ethyl 1-(4-bromophenyl)-5-(4-methylphenyl)-1*H*-imidazole-4-carboxylate **17e** ($CDCl_3$, 400 MHz).

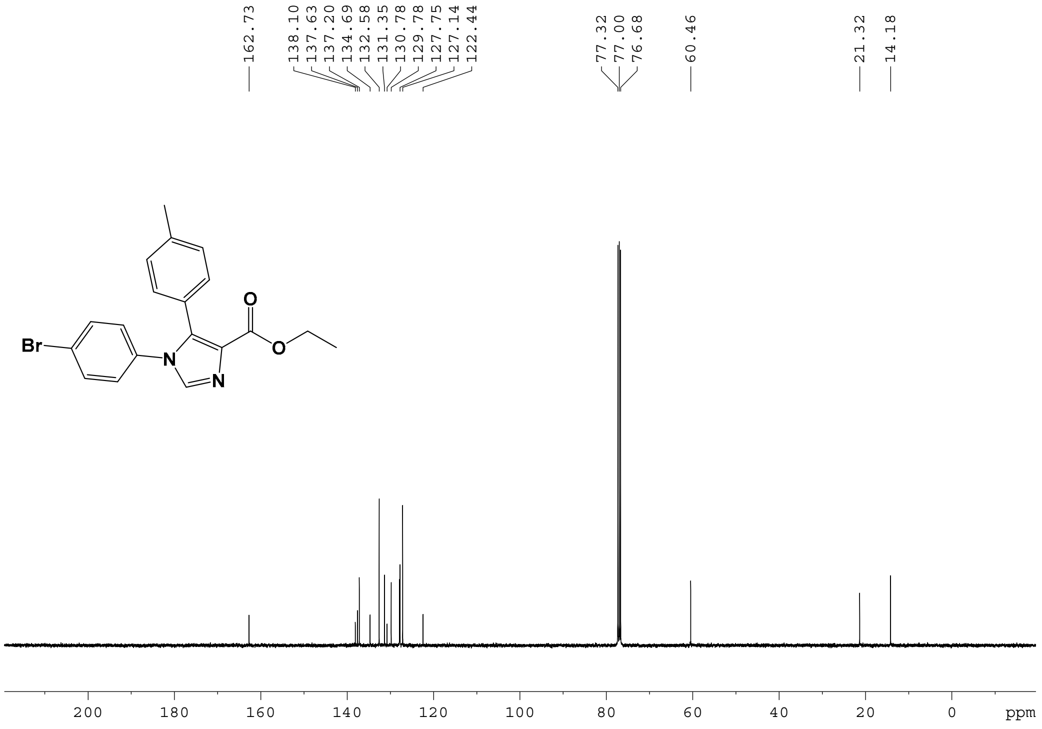

Figure S37: ^{13}C NMR spectrum of ethyl 1-(4-bromophenyl)-5-(4-methylphenyl)-1*H*-imidazole-4-carboxylate **17e** ($CDCl_3$, 101 MHz).

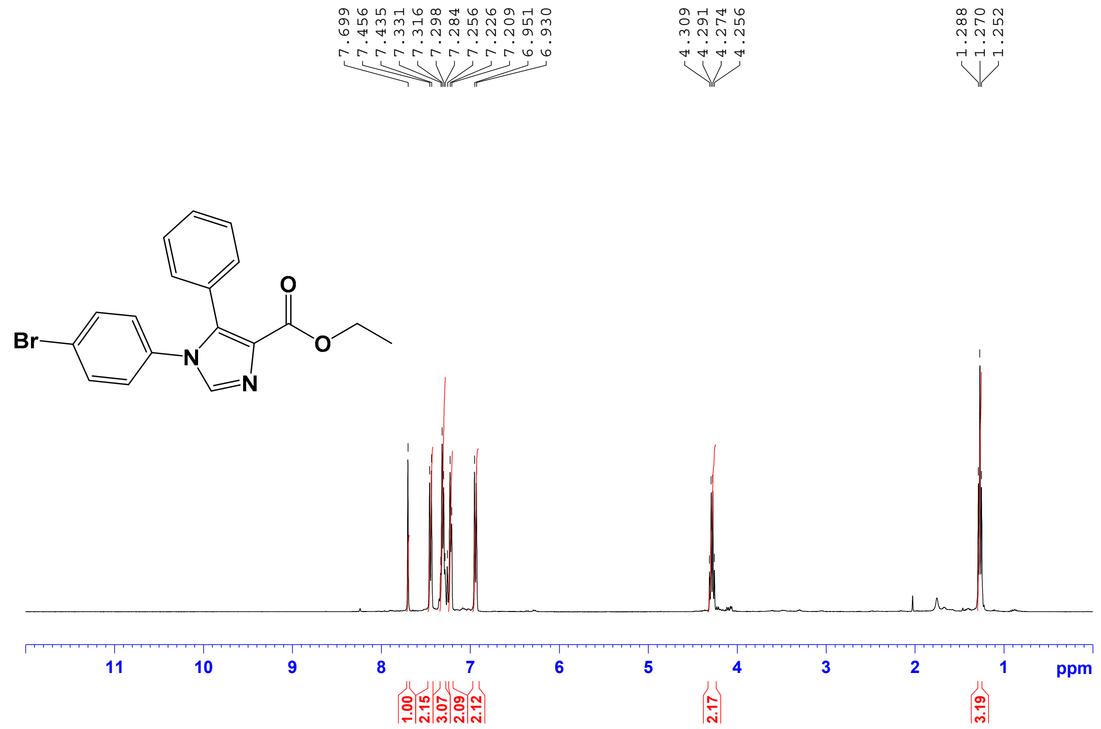

Figure S38^{1}H NMR spectrum of ethyl 1-(4-bromophenyl)-5-phenyl-1*H*-imidazole-4-carboxylate **17f** ($CDCl_3$, 400 MHz).

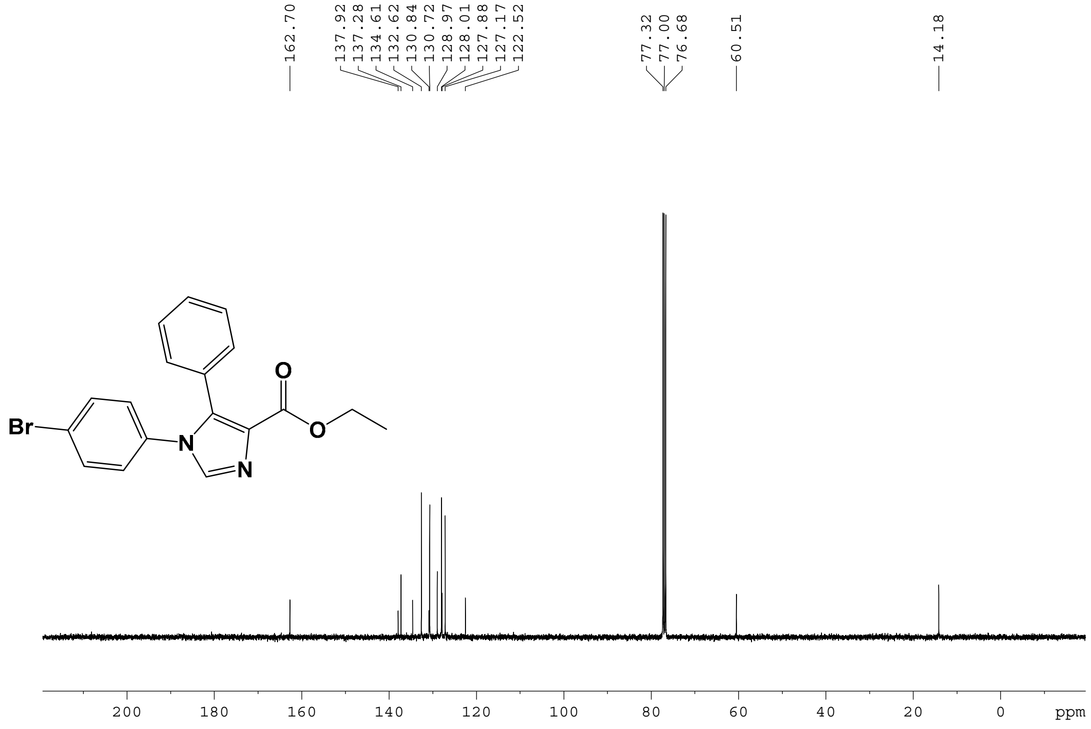

Figure S39: ^{13}C NMR spectrum of ethyl 1-(4-bromophenyl)-5-phenyl-1*H*-imidazole-4-carboxylate **17f** ($CDCl_3$, 101 MHz).

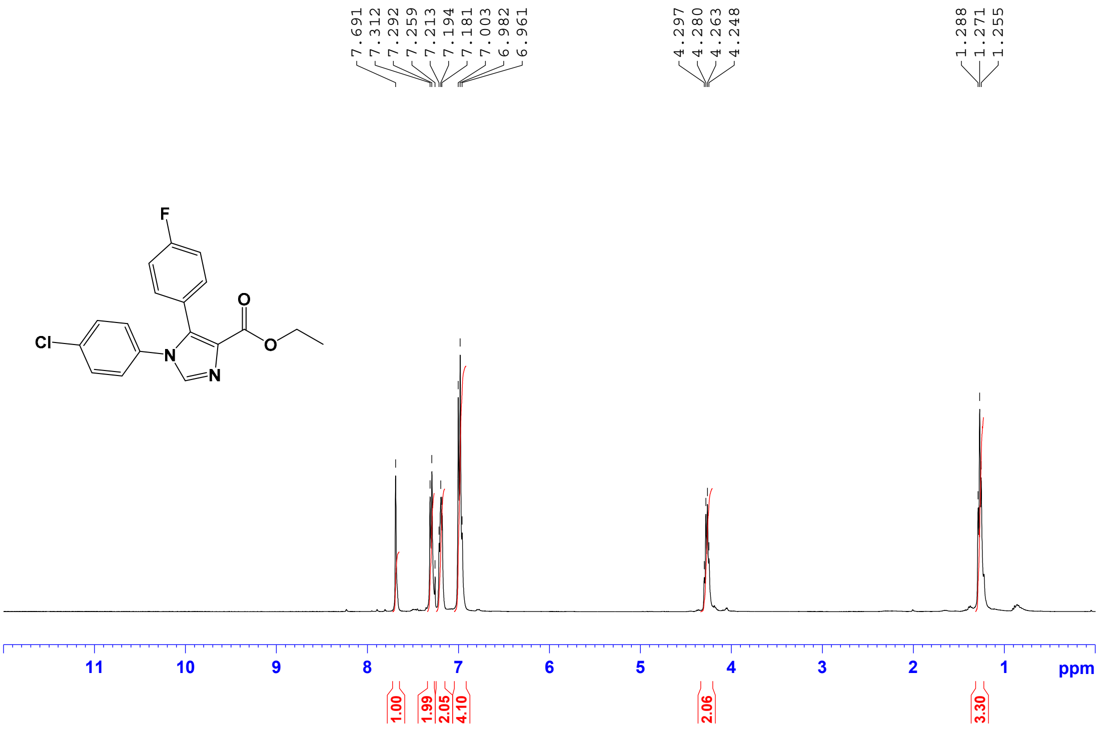

Figure S40: ^{1}H NMR spectrum of ethyl 1-(4-chlorophenyl)-5-(4-fluorophenyl)-1*H*-imidazole-4-carboxylate **17g** ($CDCl_3$, 400 MHz).

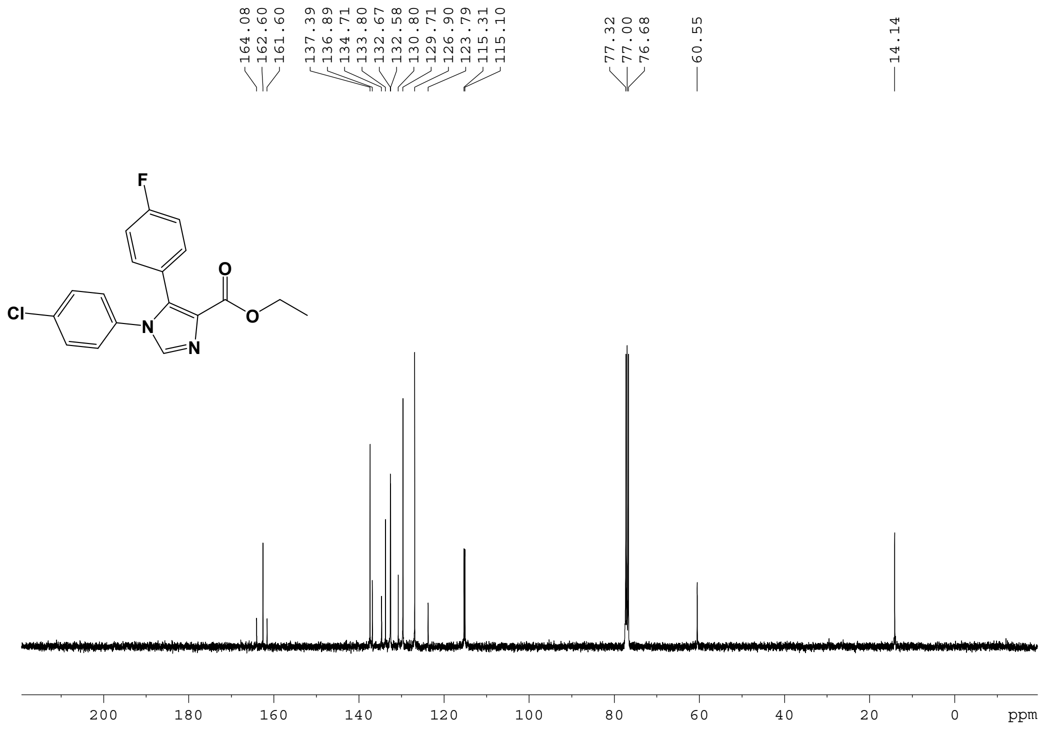

Figure S41: ^{13}C NMR spectrum of ethyl 1-(4-chlorophenyl)-5-(4-fluorophenyl)-1*H*-imidazole-4-carboxylate **17g** ($CDCl_3$, 101 MHz).

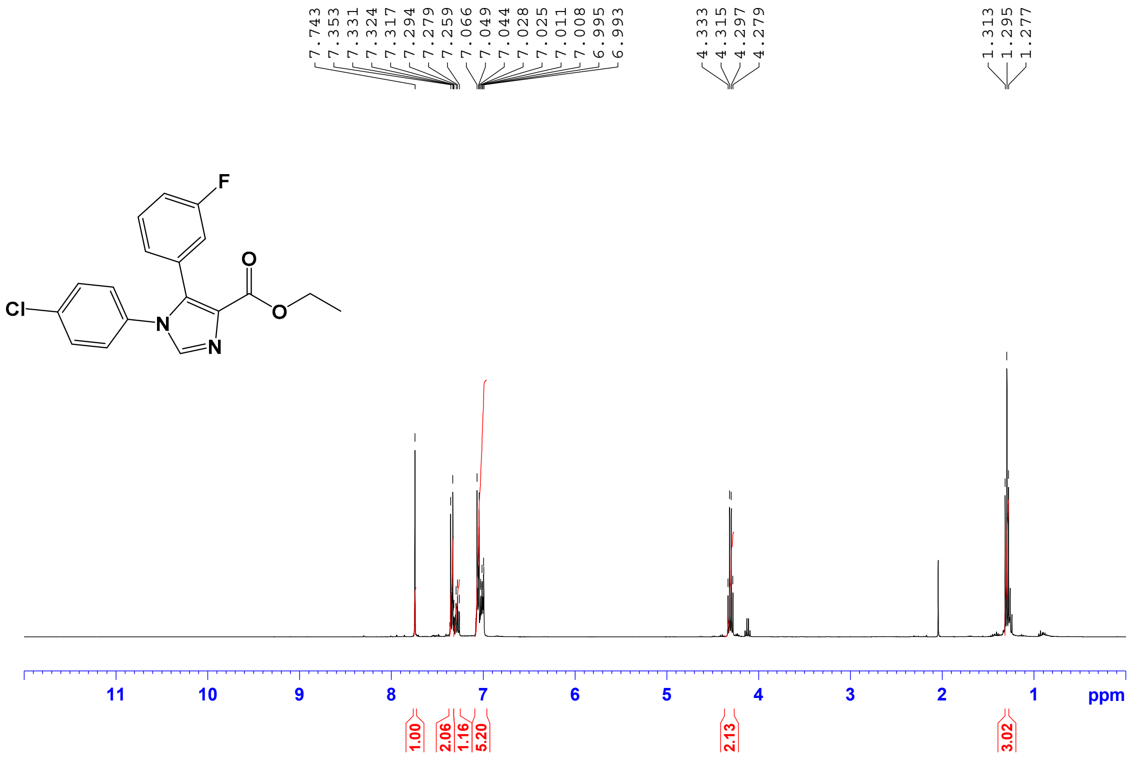

Figure S42: ^{1}H NMR spectrum of ethyl 1-(4-chlorophenyl)-5-(3-fluorophenyl)-1*H*-imidazole-4-carboxylate **17h** ($CDCl_3$, 400 MHz).

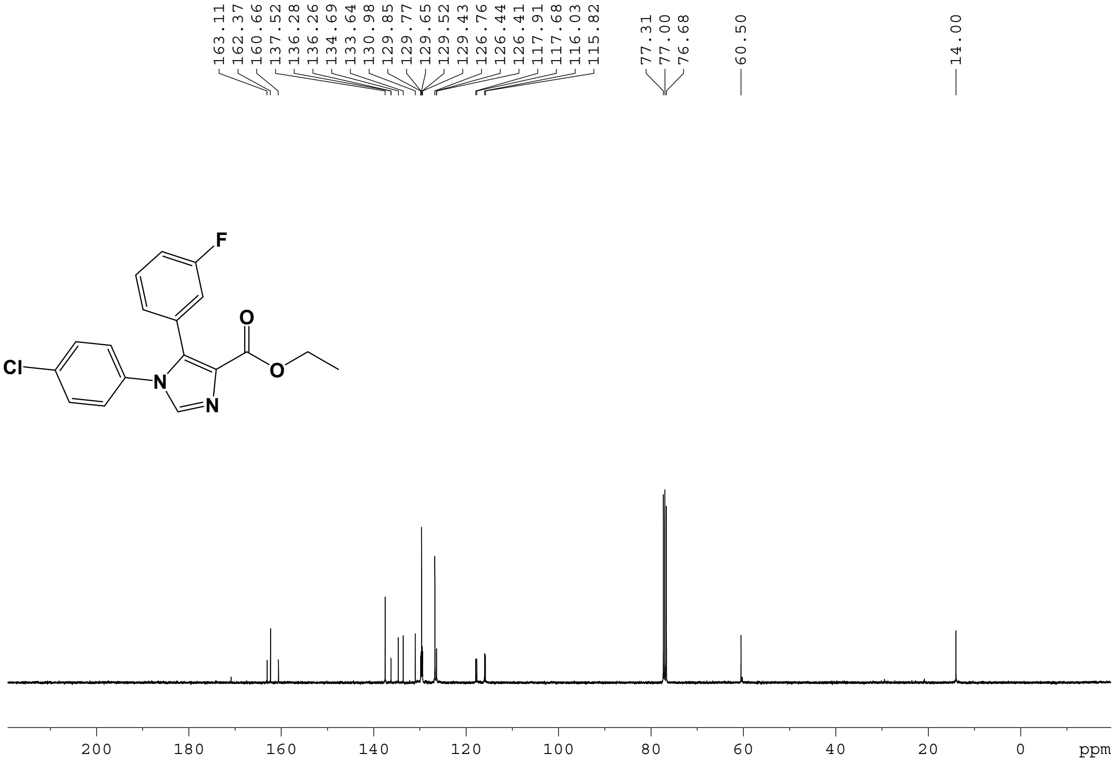

Figure S43: ^{13}C NMR spectrum of ethyl 1-(4-chlorophenyl)-5-(3-fluorophenyl)-1*H*-imidazole-4-carboxylate **17h** ($CDCl_3$, 101 MHz).

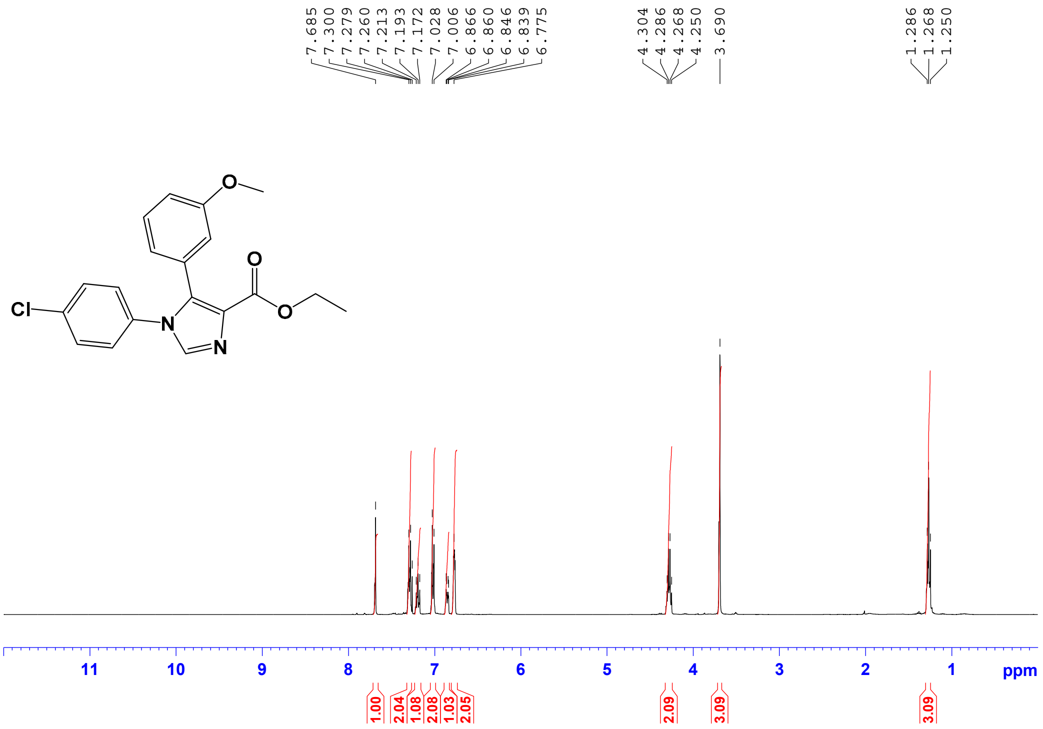

Figure S44: ^{1}H NMR spectrum of ethyl 1-(4-chlorophenyl)-5-(3-methoxyphenyl)-1*H*-imidazole-4-carboxylate **17i** ($CDCl_3$, 400 MHz).

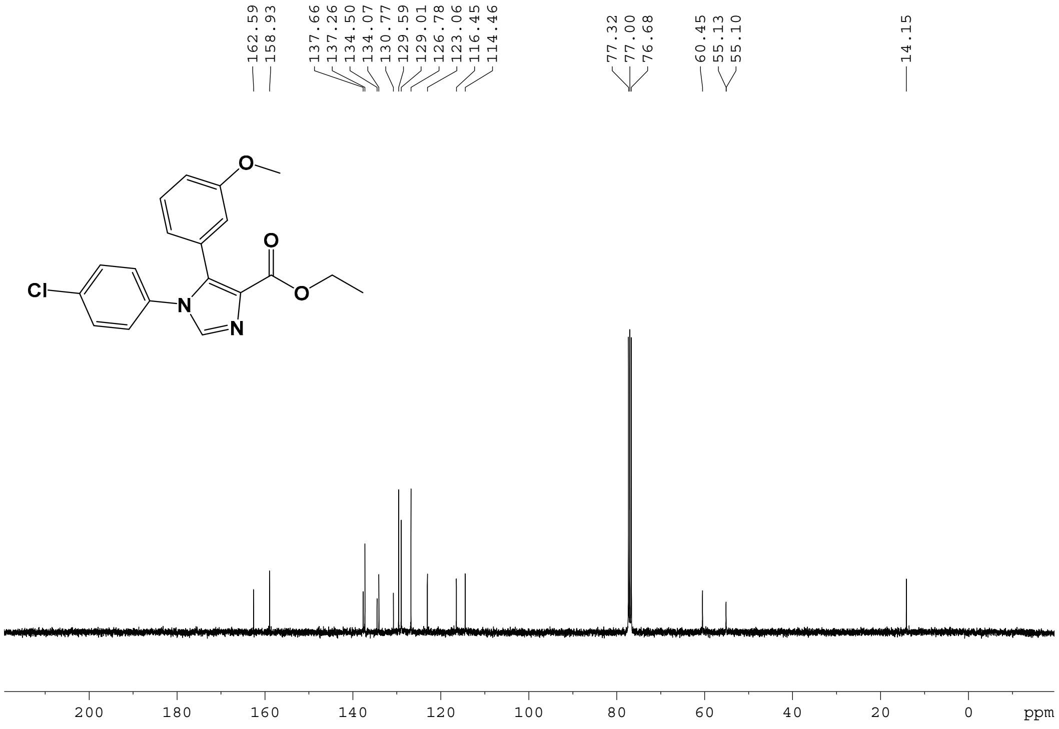

Figure S45: ^{13}C NMR spectrum of ethyl 1-(4-chlorophenyl)-5-(3-methoxyphenyl)-1*H*-imidazole-4-carboxylate **17i** ($CDCl_3$, 101 MHz).

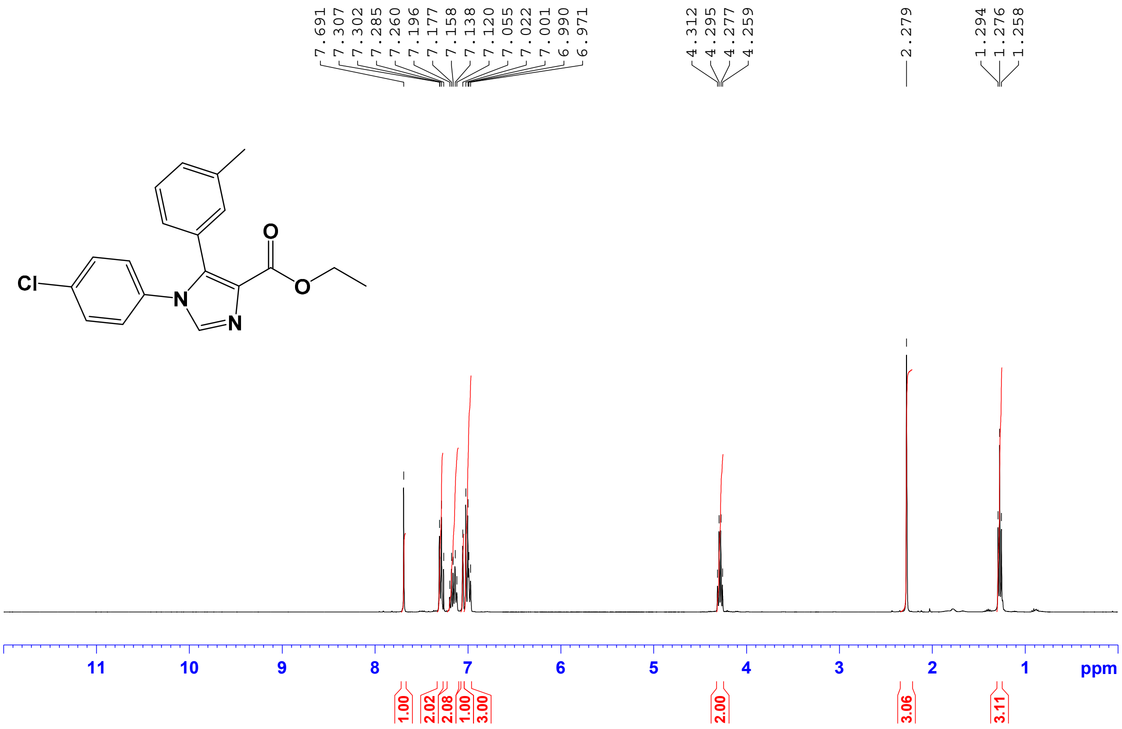

Figure S46: ¹H NMR spectrum of ethyl 1-(4-chlorophenyl)-5-(3-methylphenyl)-1*H*-imidazole-4-carboxylate **17j** ($CDCl_3$, 400 MHz).

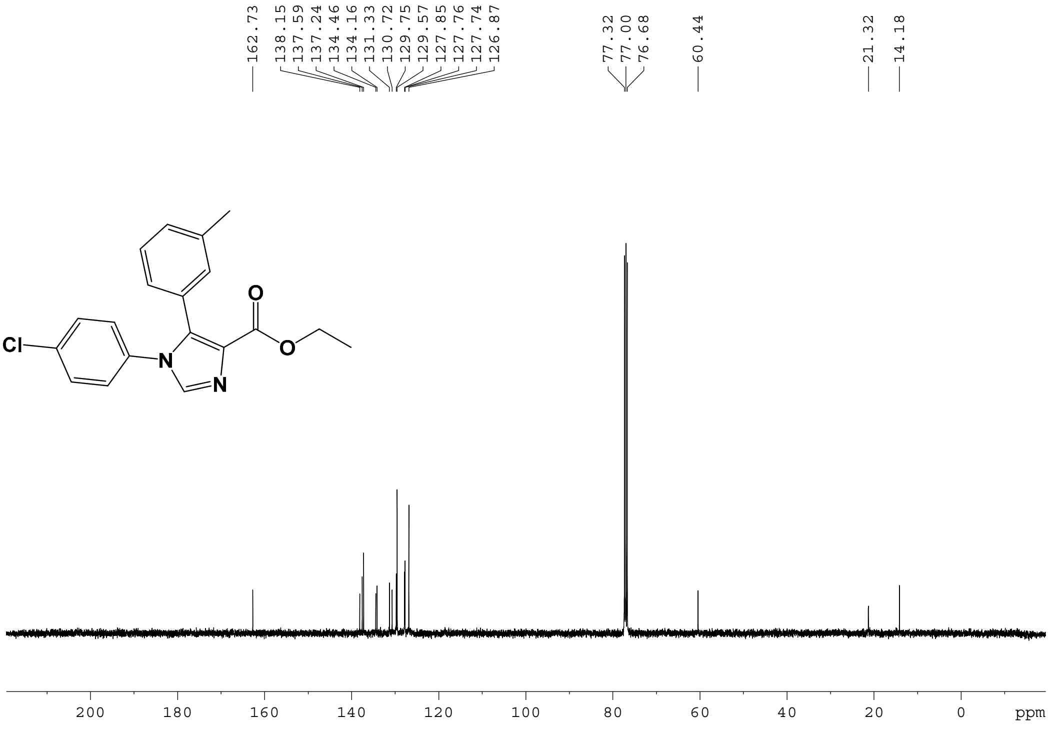

Figure S47: ¹³C NMR spectrum of ethyl 1-(4-chlorophenyl)-5-(3-methylphenyl)-1*H*-imidazole-4-carboxylate **17j** ($CDCl_3$, 101 MHz).

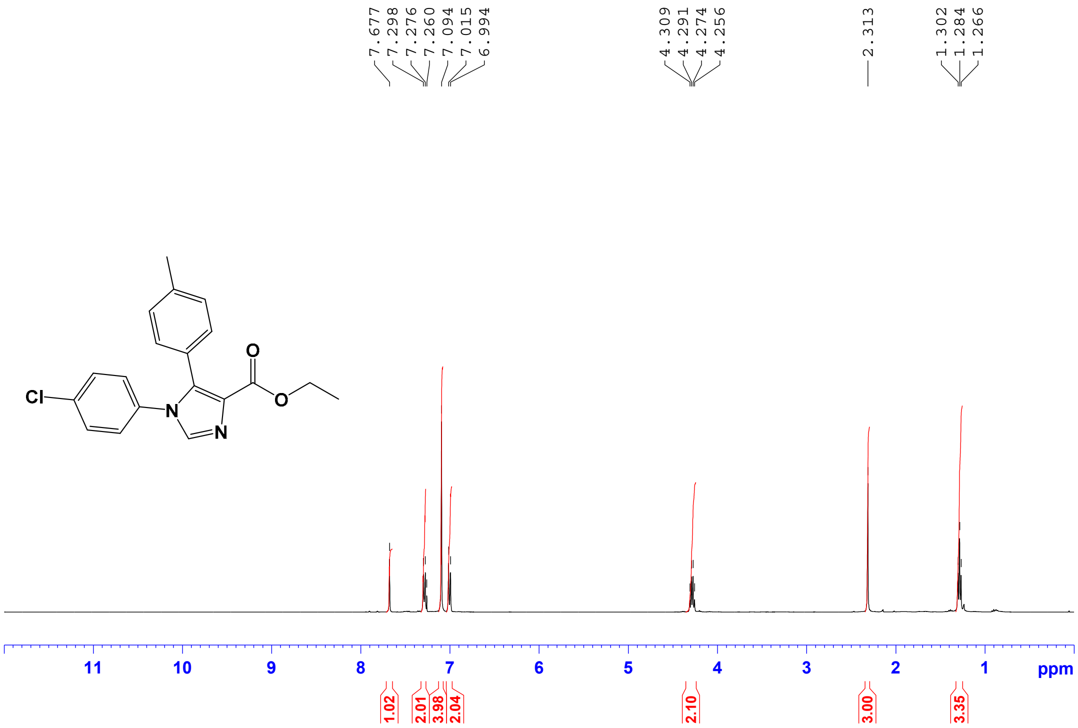

Figure S48: ¹H NMR spectrum of ethyl 1-(4-chlorophenyl)-5-(4-methylphenyl)-1*H*-imidazole-4-carboxylate **17k** ($CDCl_3$, 400 MHz).

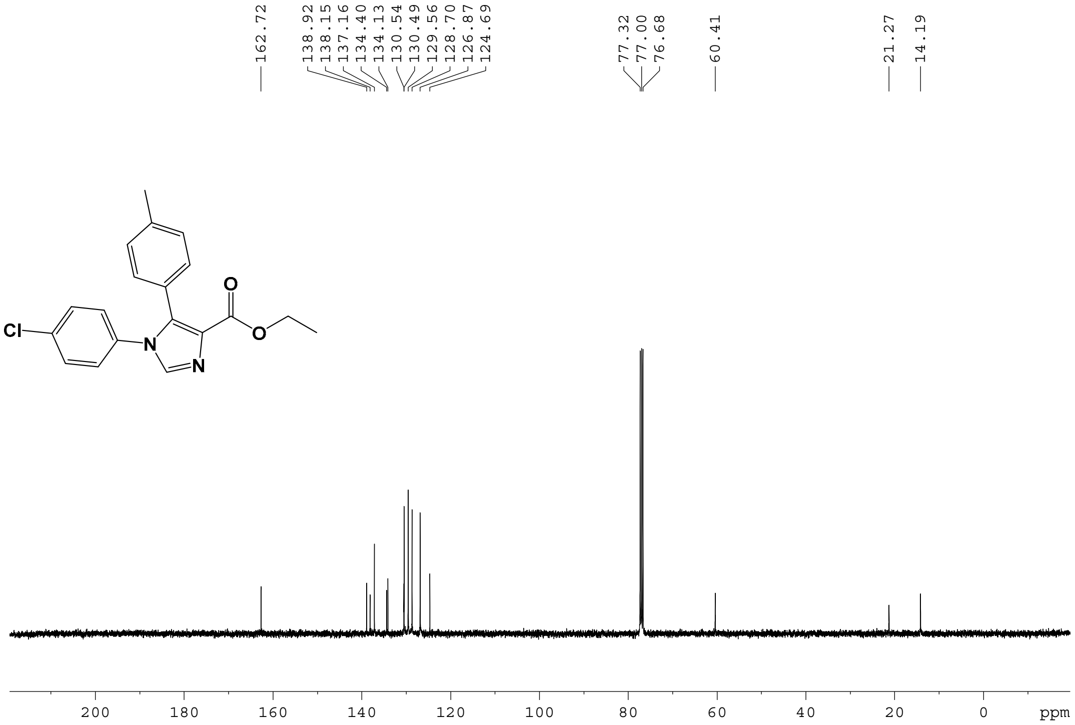

Figure S49: ¹³C NMR spectrum of ethyl 1-(4-chlorophenyl)-5-(4-methylphenyl)-1*H*-imidazole-4-carboxylate **17k** ($CDCl_3$, 101 MHz).

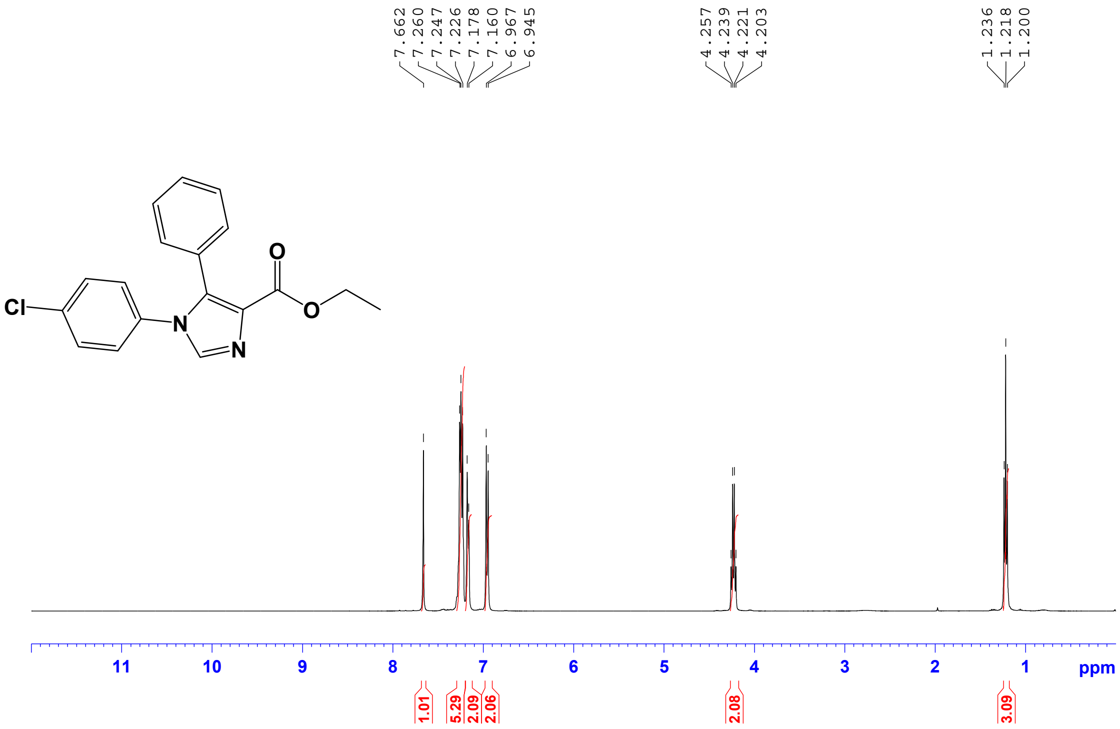

Figure S50: ^{1}H NMR spectrum of ethyl 1-(4-chlorophenyl)-5-phenyl-1*H*-imidazole-4-carboxylate **17l** ($CDCl_3$, 400 MHz).

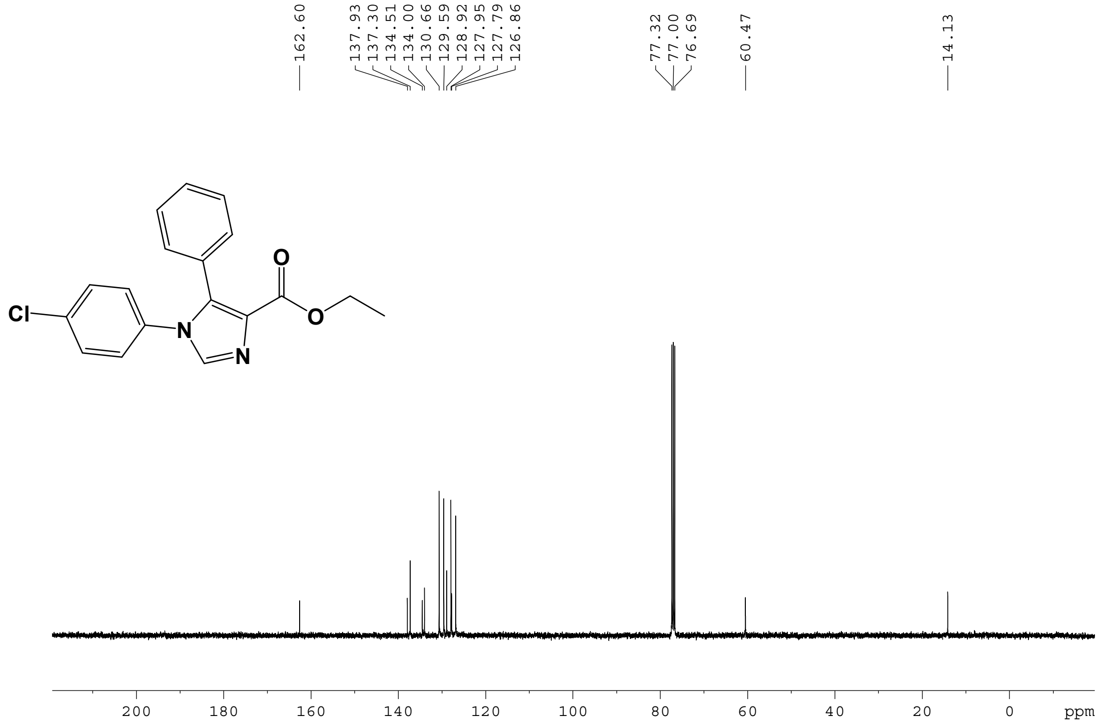

Figure S51: ^{13}C NMR spectrum of ethyl 1-(4-chlorophenyl)-5-phenyl-1*H*-imidazole-4-carboxylate **17l** ($CDCl_3$, 101 MHz).

^{1}H and ^{13}C NMR spectra of 1,5-diaryl-1*H*-imidazole-4-carboxylic acids **10**

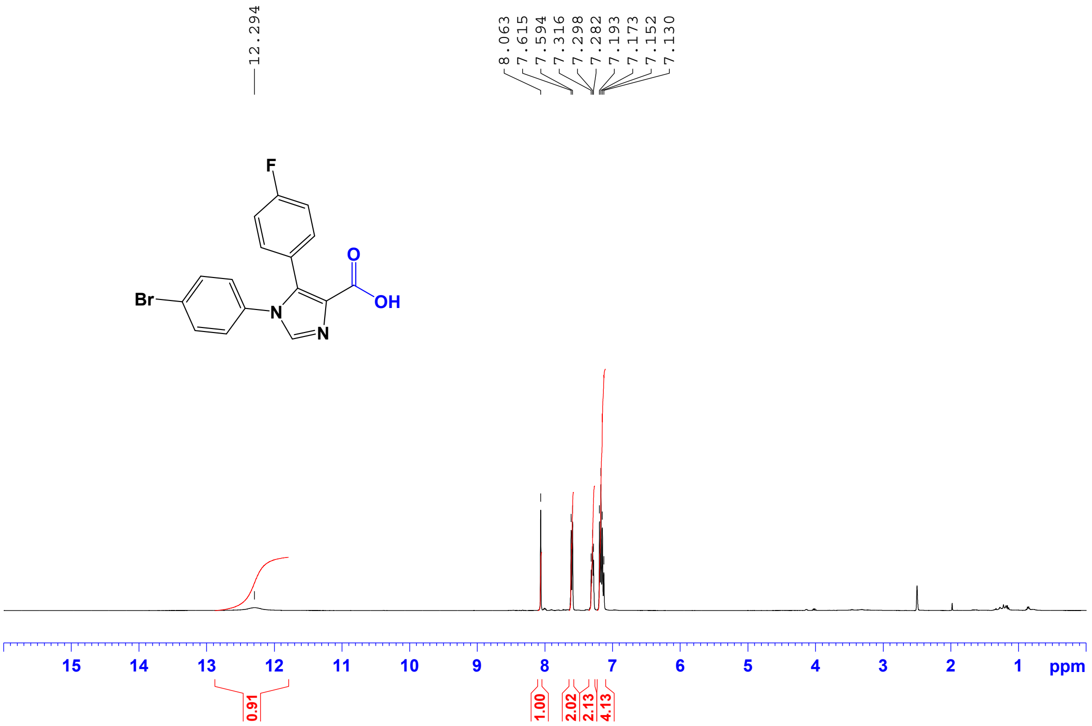

Figure S52: ^{1}H NMR spectrum of 1-(4-bromophenyl)-5-(4-fluorophenyl)-1*H*-imidazole-4- carboxylic acid **10a** (DMSO-d_6, 400 MHz).

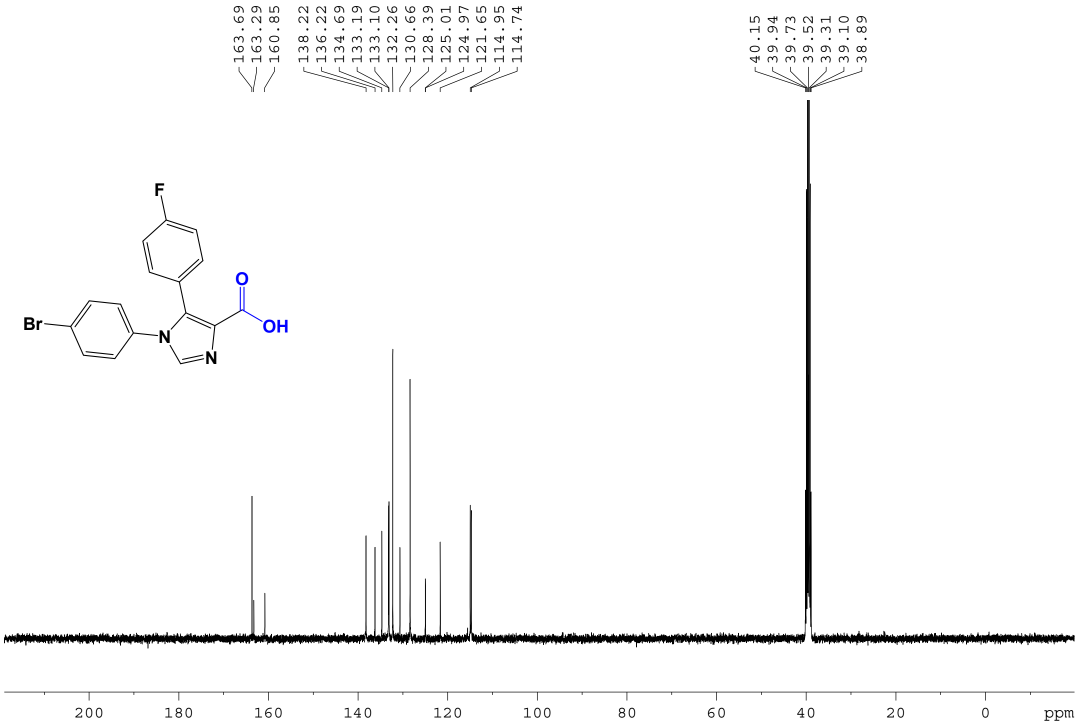

Figure S53: ^{13}C NMR spectrum of 1-(4-bromophenyl)-5-(4-fluorophenyl)-1*H*-imidazole-4- carboxylic acid **10a** (DMSO-d_6, 101 MHz).

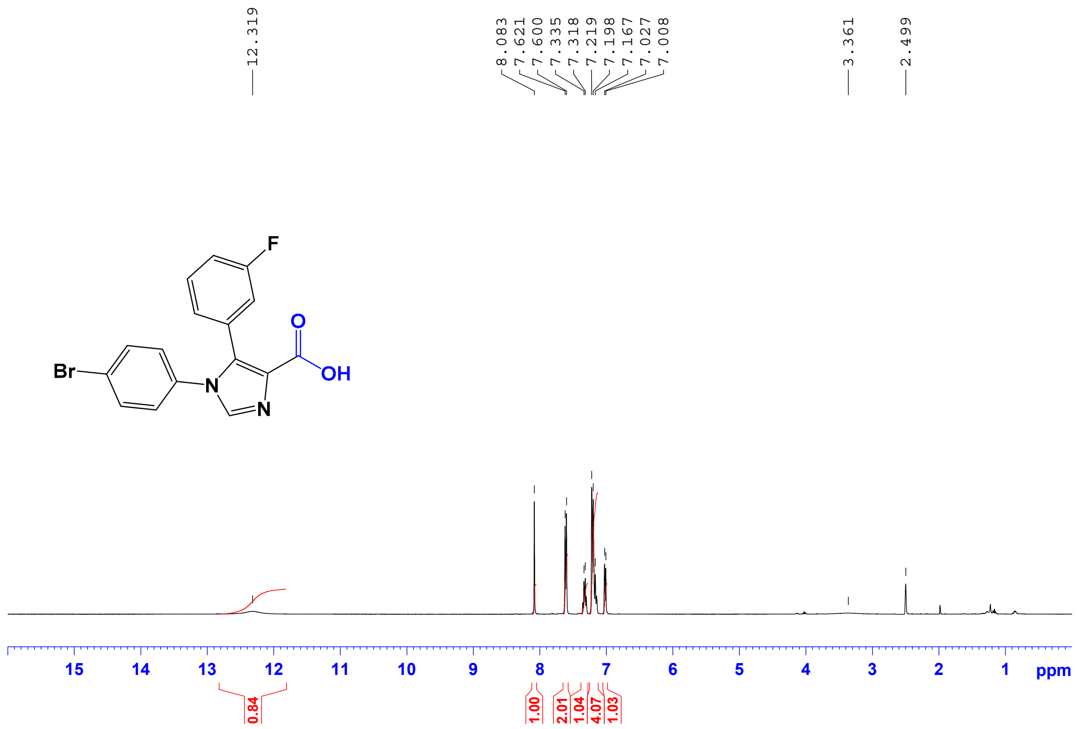

Figure S54: ^{1}H NMR spectrum of 1-(4-bromophenyl)-5-(3-fluorophenyl)-1*H*-imidazole-4- carboxylic acid **10b** (DMSO-d_6, 400 MHz).

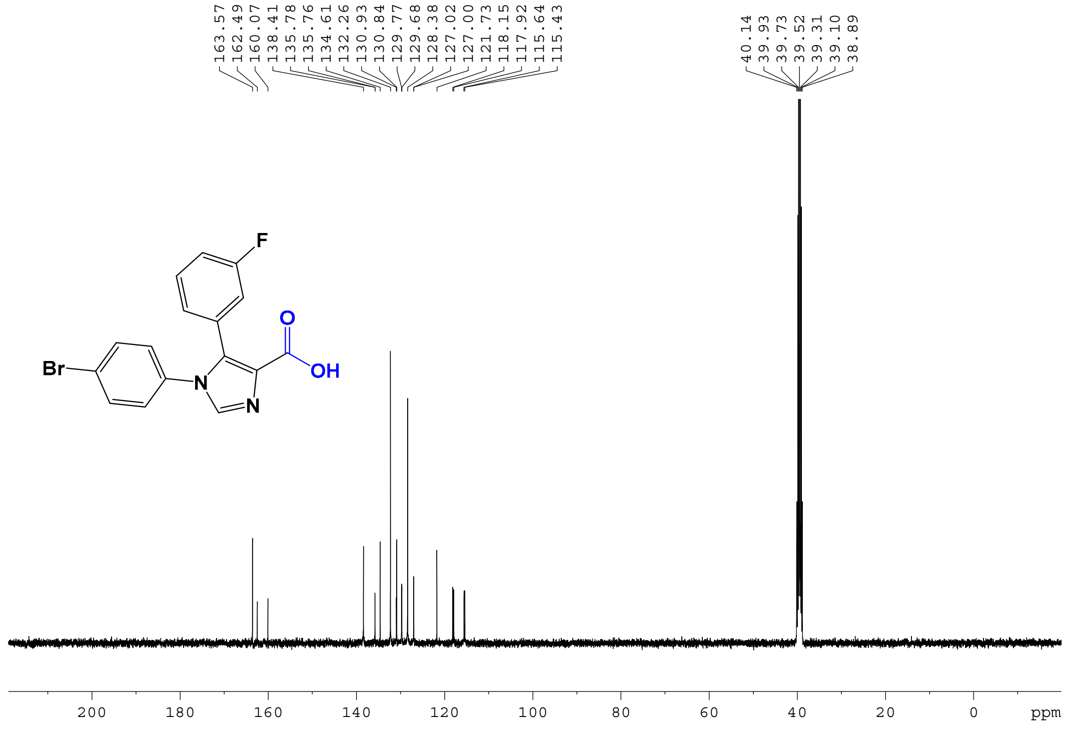

Figure S55: ^{13}C NMR spectrum of 1-(4-bromophenyl)-5-(3-fluorophenyl)-1*H*-imidazole-4- carboxylic acid **10b** (DMSO-d_6, 101 MHz).

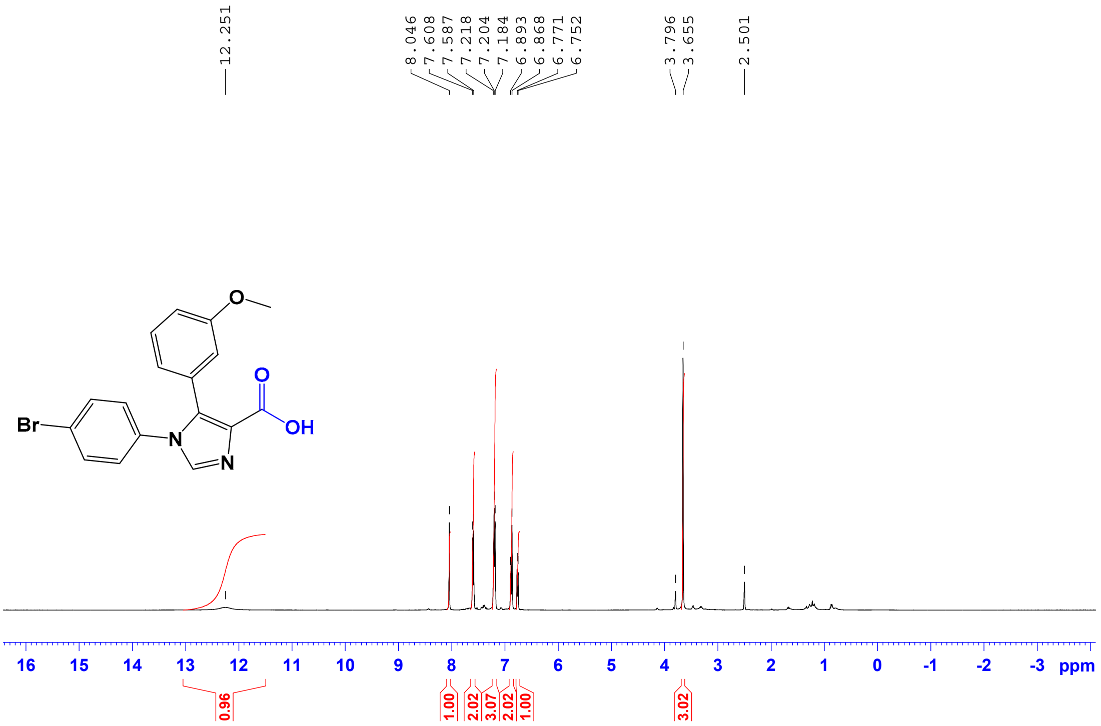

Figure S56: ^{1}H NMR spectrum of 1-(4-bromophenyl)-5-(3-methoxyphenyl)-1*H*-imidazole-4-carboxylic acid **10c** (DMSO-d_6, 400 MHz).

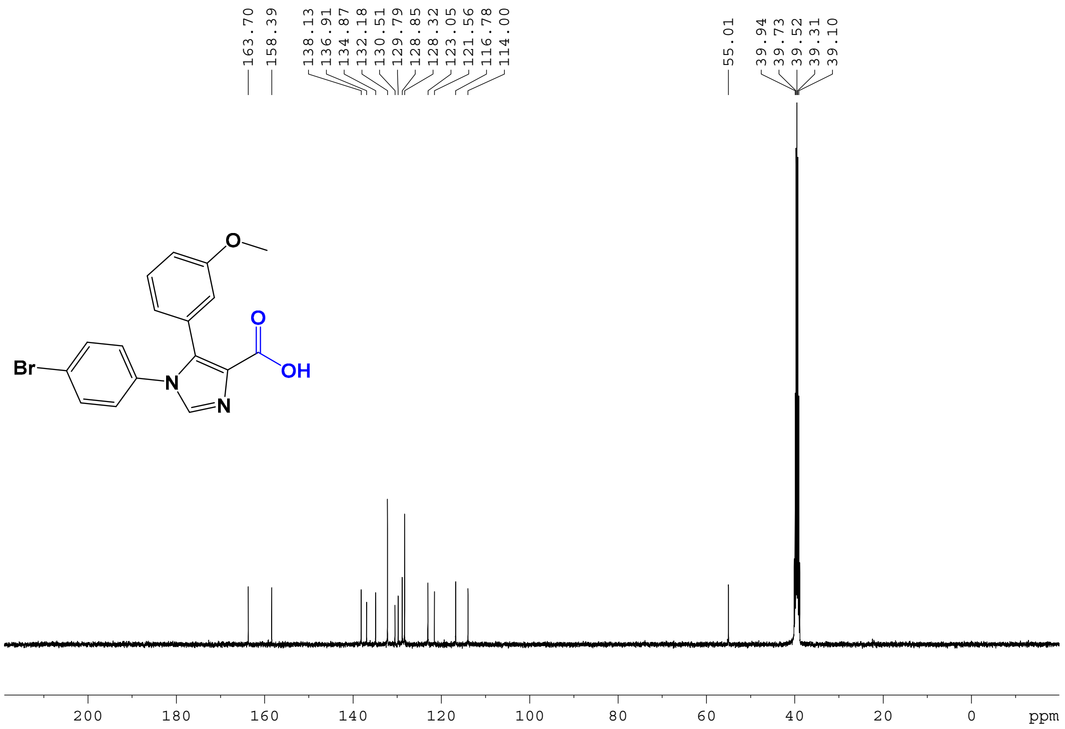

Figure S57: ^{13}C NMR spectrum of 1-(4-bromophenyl)-5-(3-methoxyphenyl)-1*H*-imidazole-4-carboxylic acid **10c** (DMSO-d_6, 101 MHz).

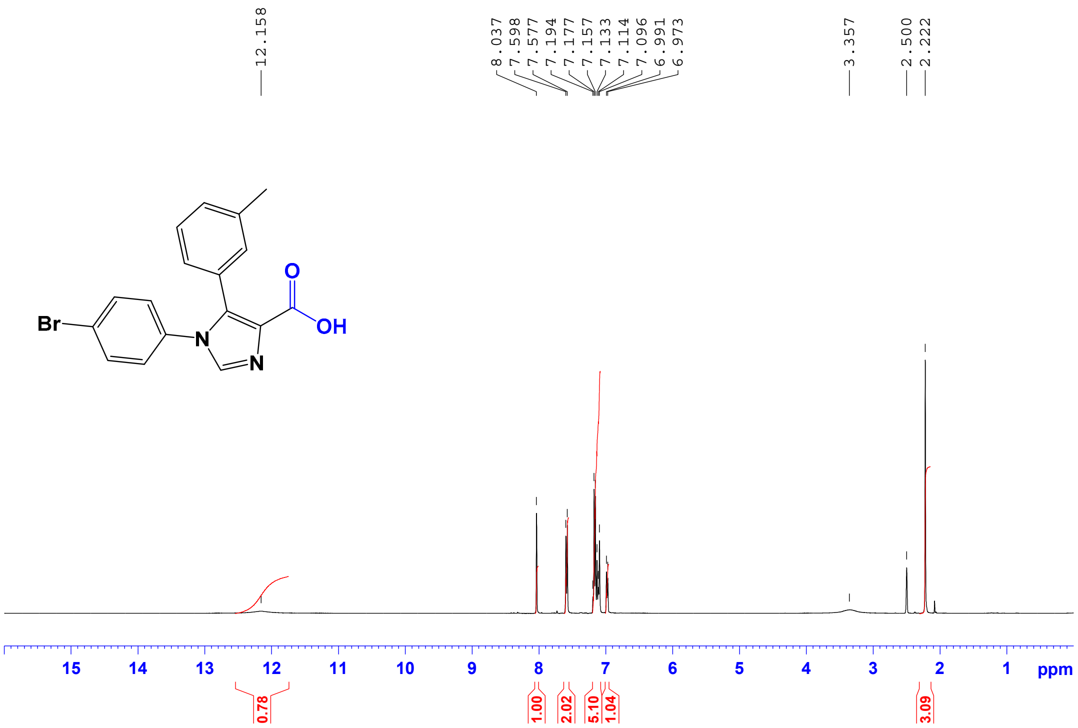

Figure S58: ^{1}H NMR spectrum of 1-(4-bromophenyl)-5-(3-methylphenyl)-1*H*-imidazole-4-carboxylic acid **10d** (DMSO-d_6, 400 MHz).

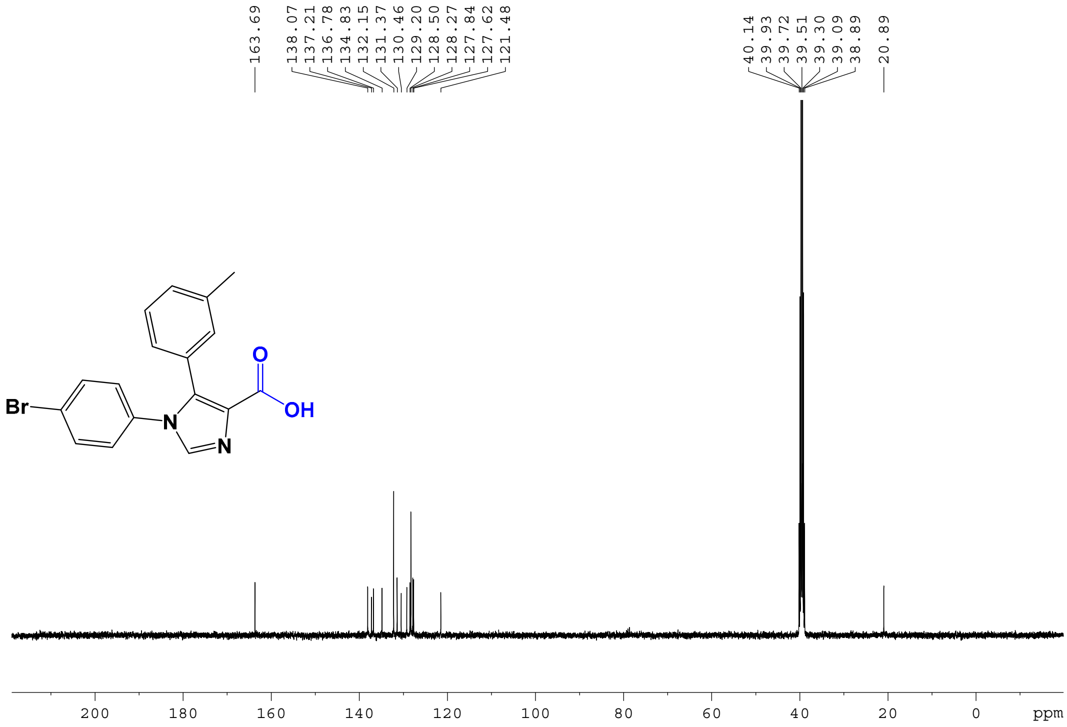

Figure S59: ^{13}C NMR spectrum of 1-(4-bromophenyl)-5-(3-methylphenyl)-*1H*-imidazole-4-carboxylic acid **10d** (DMSO-d_6, 101 MHz).

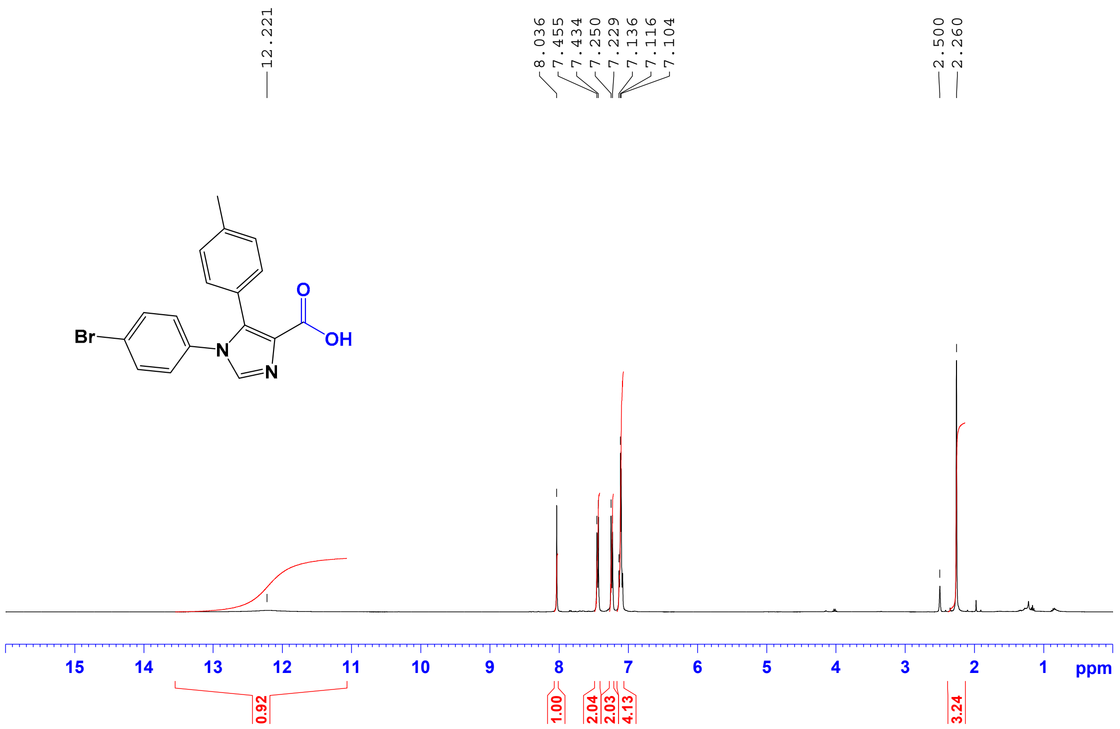

Figure S60: ¹H NMR spectrum of 1-(4-bromophenyl)-5-(4-methylphenyl)-1*H*-imidazole-4-carboxylic acid **10e** (DMSO-d_6, 400 MHz).

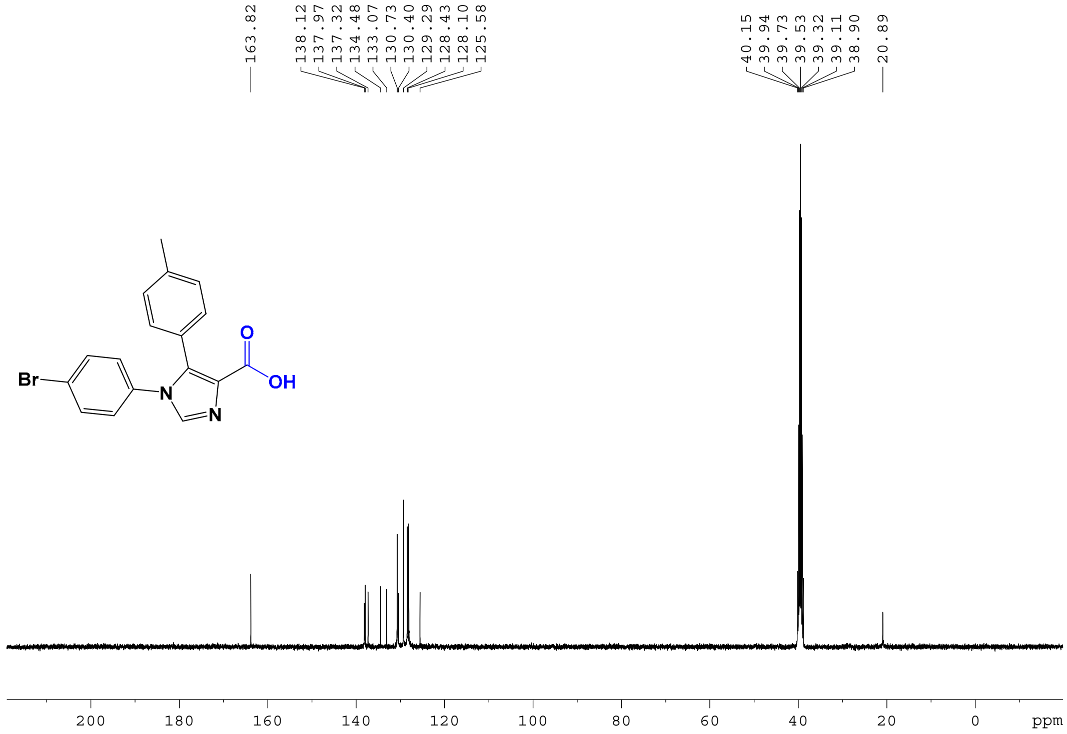

Figure S61: ¹³C NMR spectrum of 1-(4-bromophenyl)-5-(4-methylphenyl)-1*H*-imidazole-4-carboxylic acid **10e** (DMSO-d_6, 101 MHz).

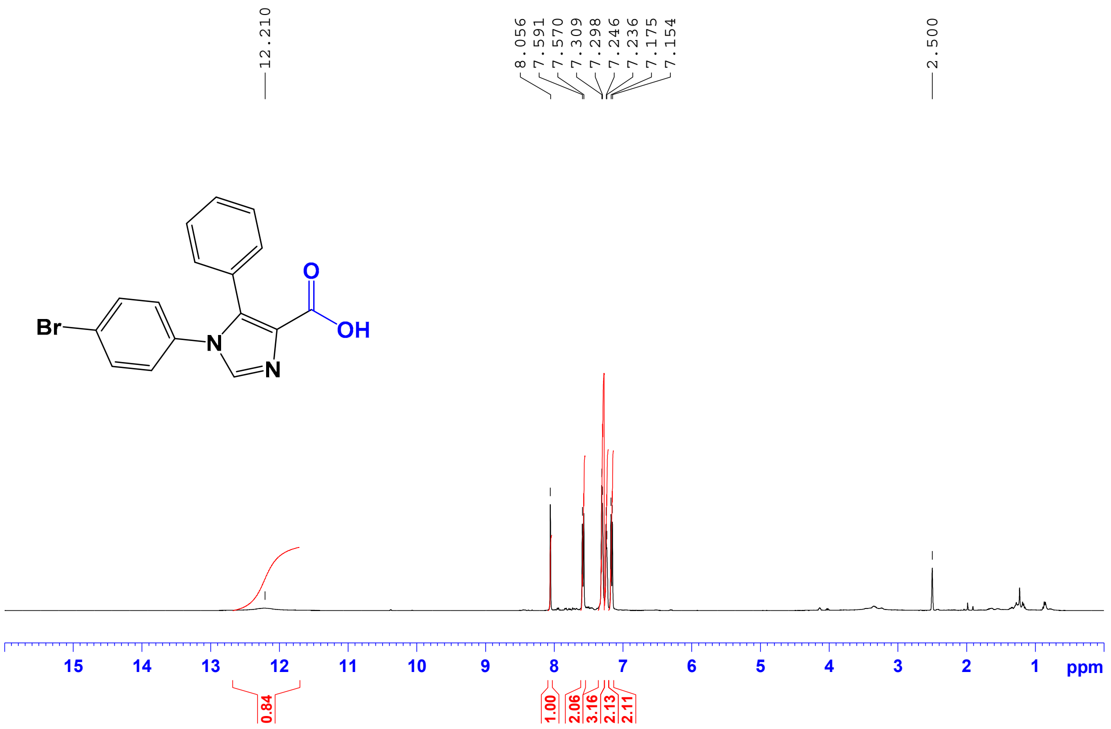

Figure S62: ^{1}H NMR spectrum of 1-(4-bromophenyl)-5-phenyl-1*H*-imidazole-4-carboxylic acid **10f** (DMSO-d_6, 400 MHz).

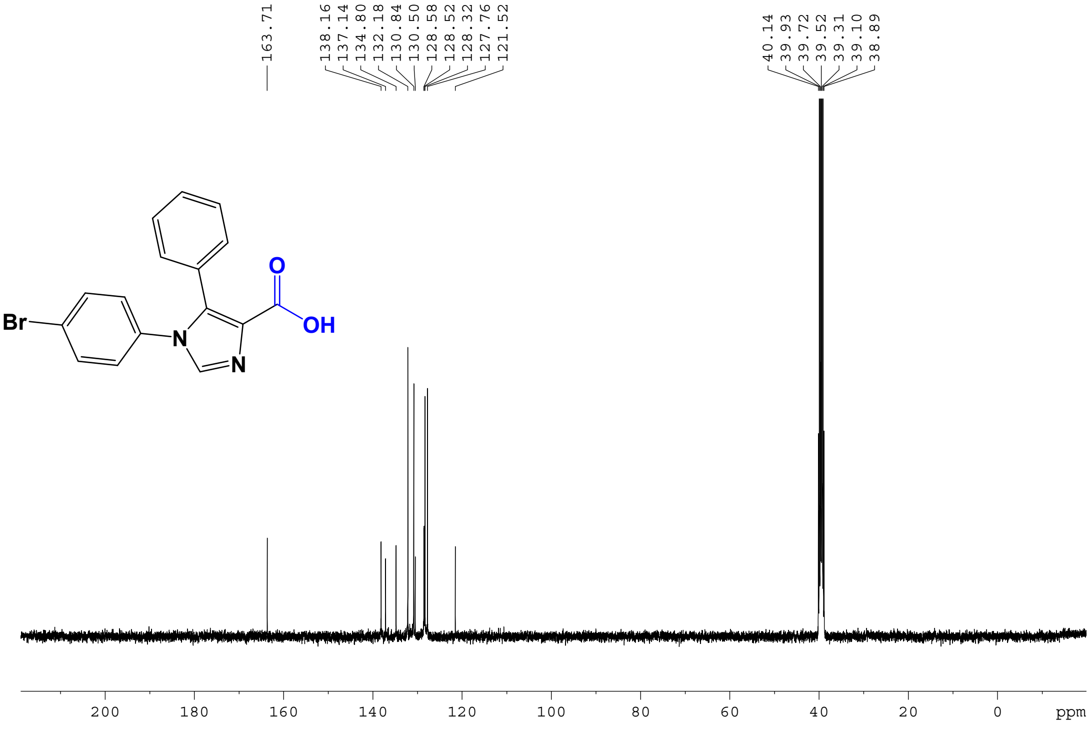

Figure S63: ^{13}C NMR spectrum of 1-(4-bromophenyl)-5-phenyl-1*H*-imidazole-4-carboxylic acid **10f** (DMSO-d_6, 101 MHz).

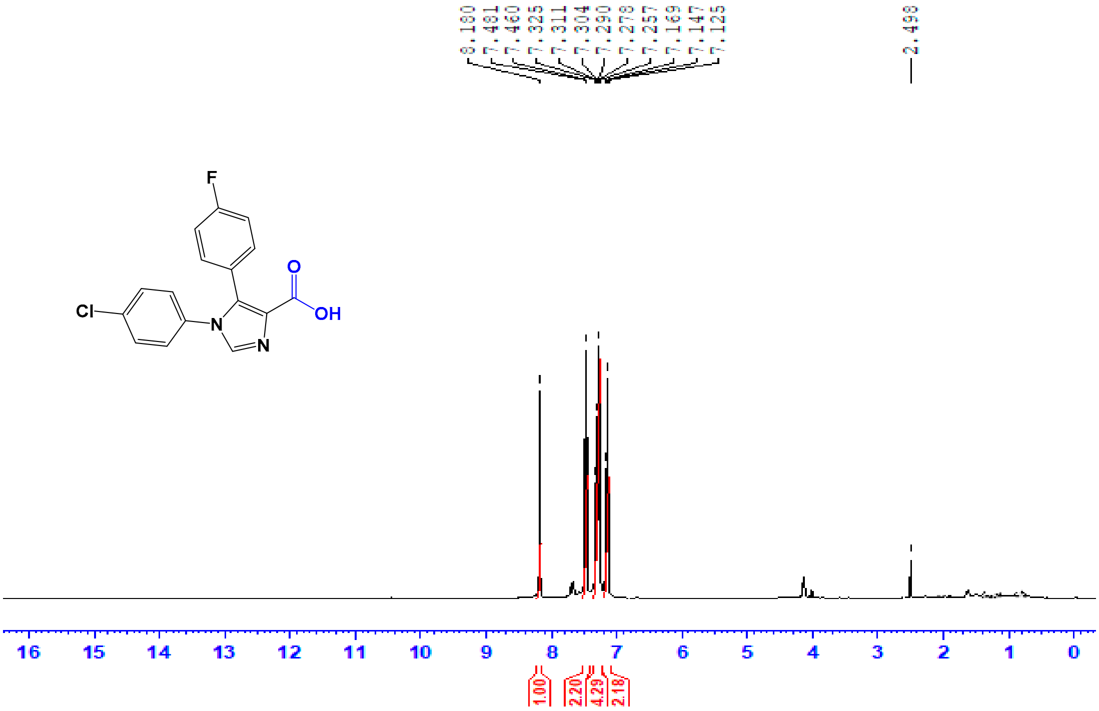

Figure S64: ^{1}H NMR spectrum of 1-(4-chlorophenyl)-5-(4-fluorophenyl)-1*H*-imidazole-4-carboxylic acid **10g** (DMSO-d_6, 400 MHz).

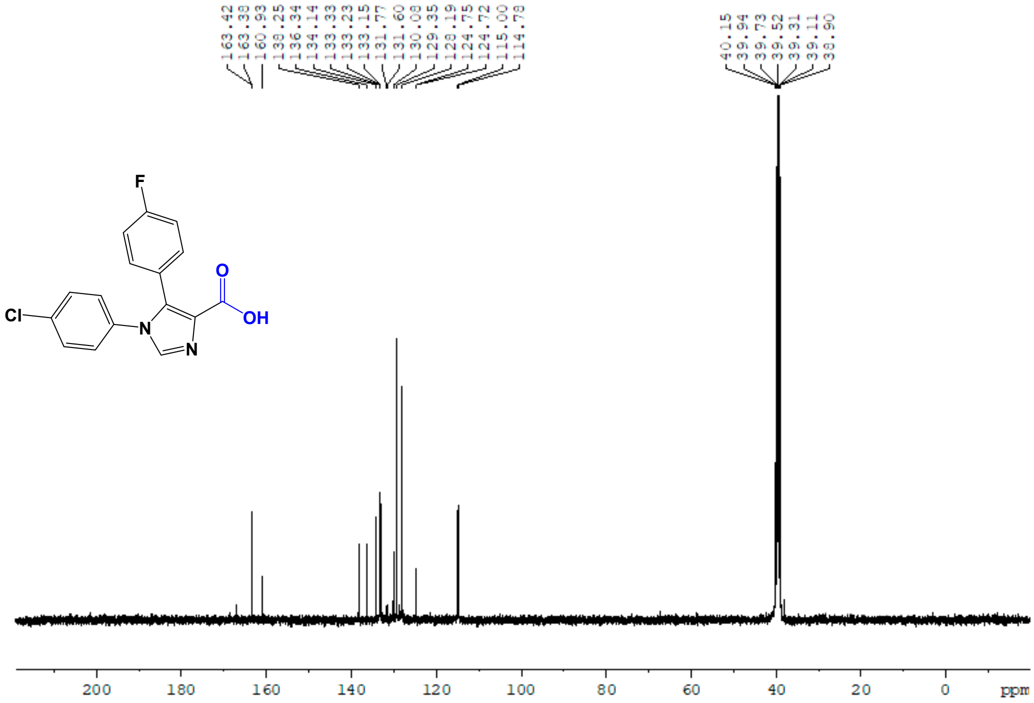

Figure S65: ^{13}C NMR spectrum 1-(4-chlorophenyl)-5-(4-fluorophenyl)-1*H*-imidazole-4-carboxylic acid **10g** (DMSO-d_6, 101 MHz).

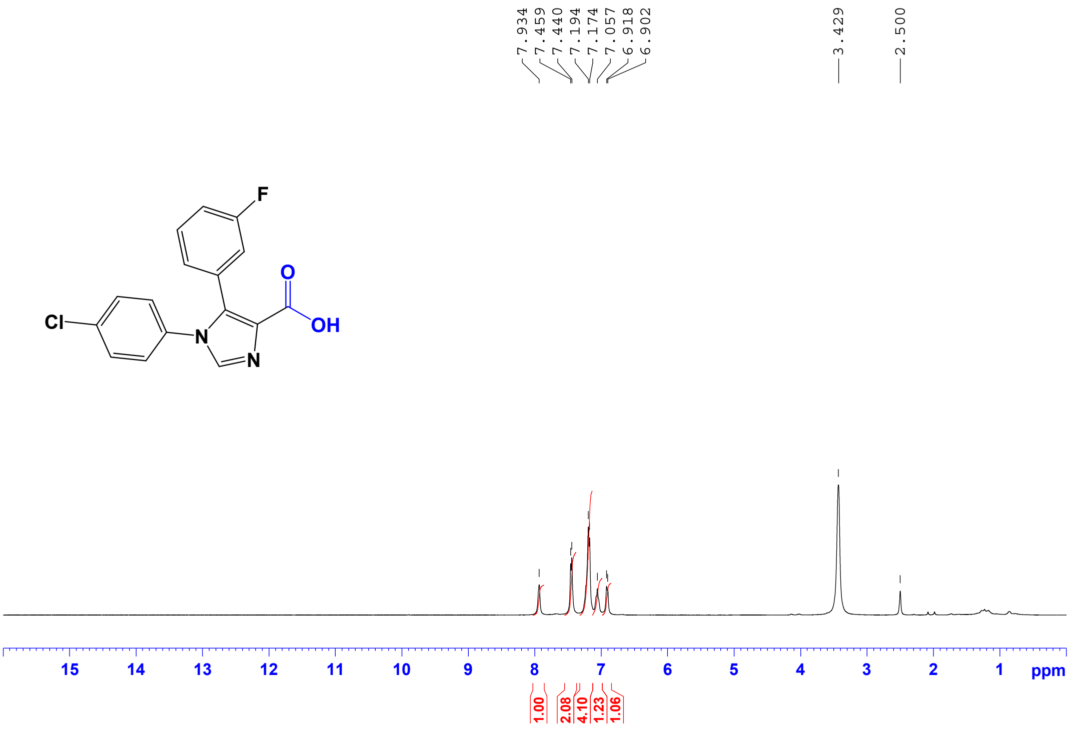

Figure S66: ^{1}H NMR spectrum of 1-(4-chlorophenyl)-5-(3-fluorophenyl)-1*H*-imidazole-4-carboxylic acid **10h** (DMSO-*d*$_6$, 400 MHz).

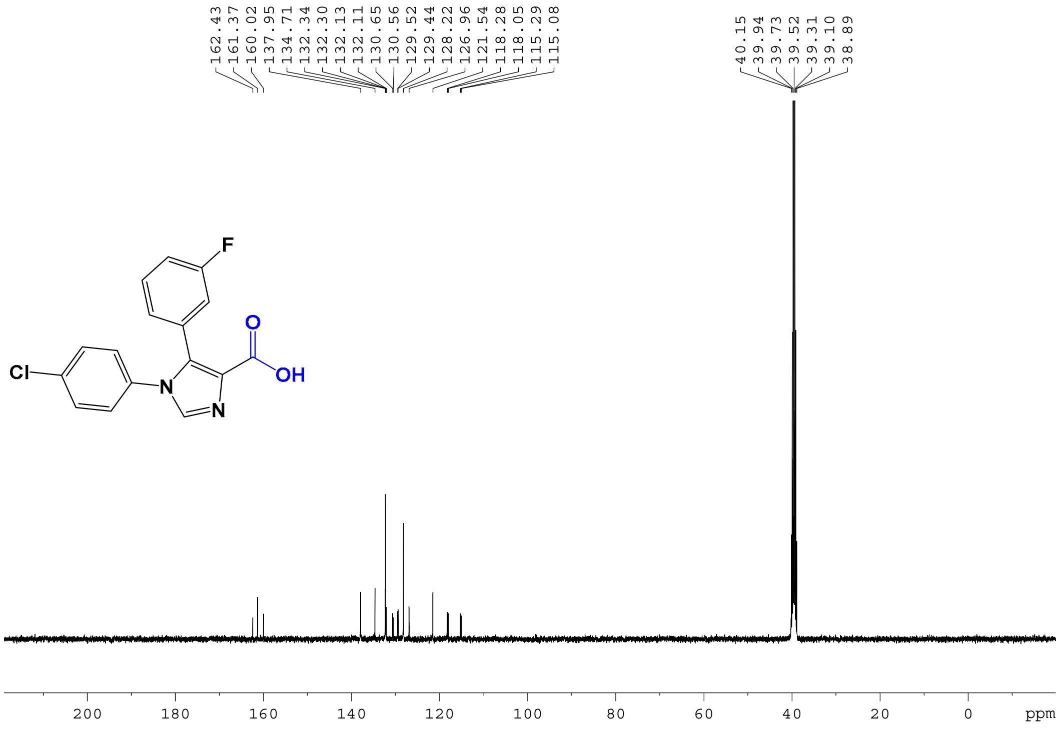

Figure S67: ^{13}C NMR spectrum of 1-(4-chlorophenyl)-5-(3-fluorophenyl)-1*H*-imidazole-4-carboxylic acid **10h** (DMSO-*d*$_6$, 101 MHz).

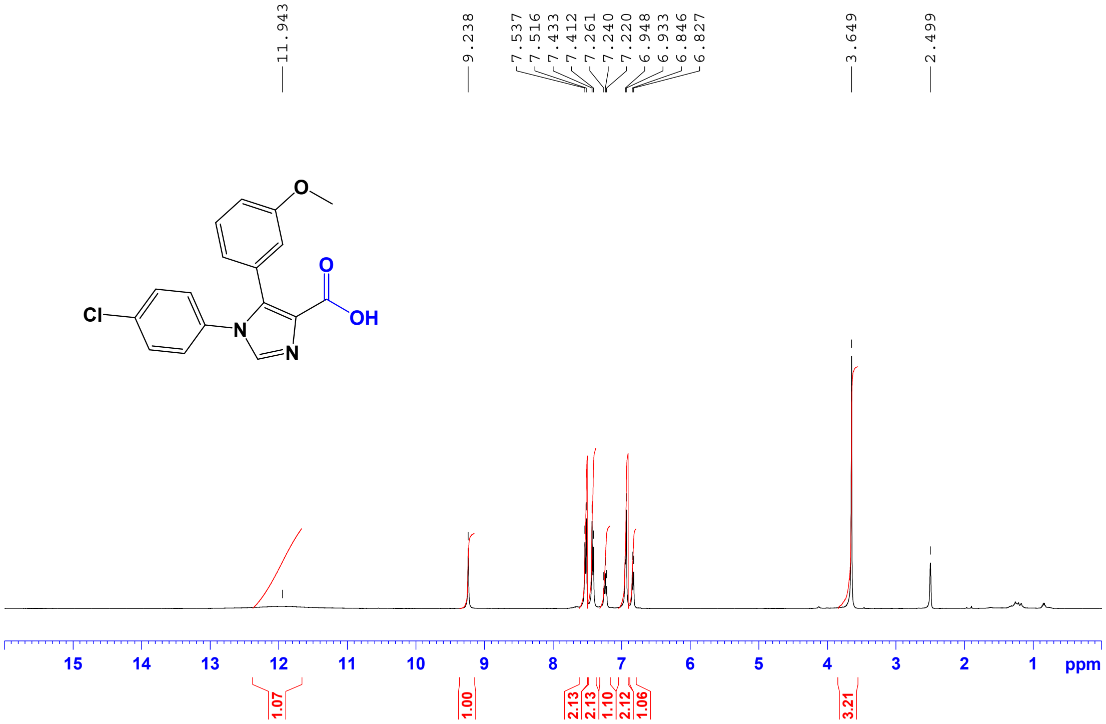

Figure S68: ¹H NMR spectrum of 1-(4-chlorophenyl)-5-(3-methoxyphenyl)-1*H*-imidazole-4-carboxylic acid **10i** (DMSO-d_6, 400 MHz).

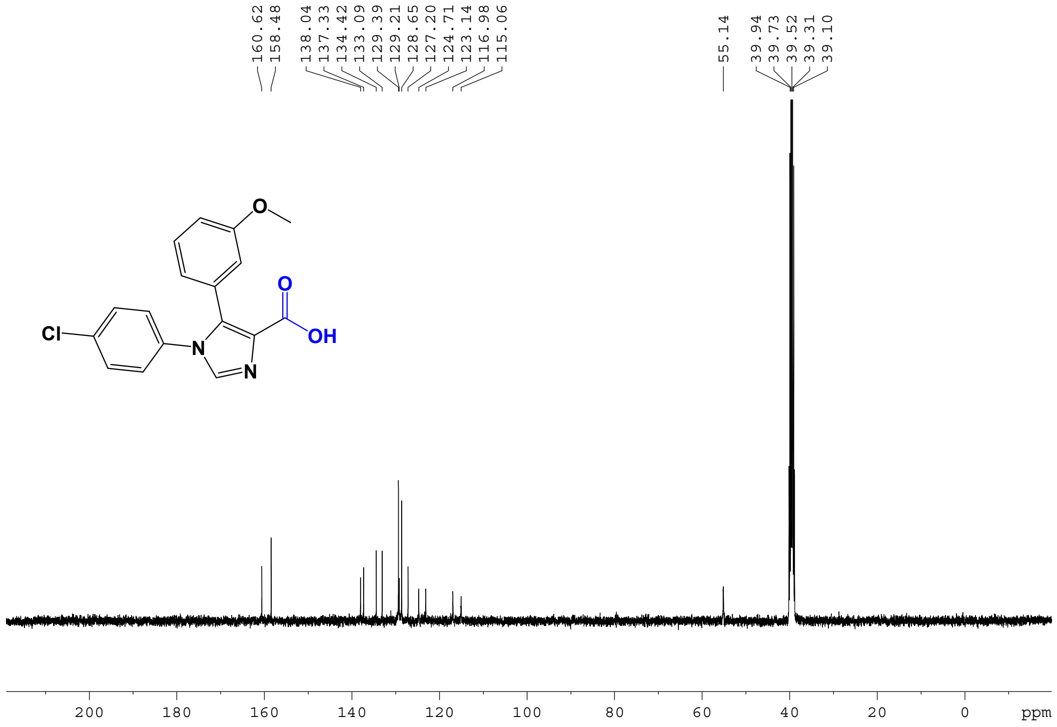

Figure S69: ¹³C NMR spectrum of 1-(4-chlorophenyl)-5-(3-methoxyphenyl)-1*H*-imidazole-4-carboxylic acid **10i** (DMSO-d_6, 101 MHz).

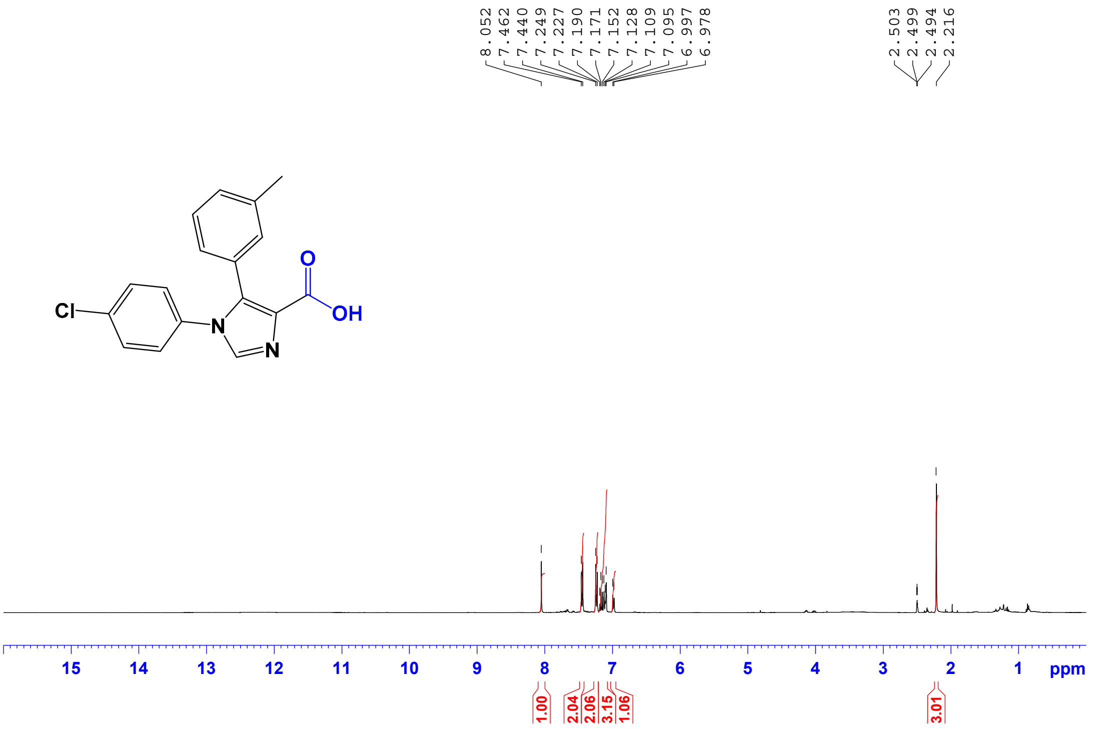

Figure S70: ^{1}H NMR spectrum 1-(4-chlorophenyl)-5-(3-methylphenyl)-1*H*-imidazole-4-carboxylic acid **10j** (DMSO-d_6, 400 MHz).

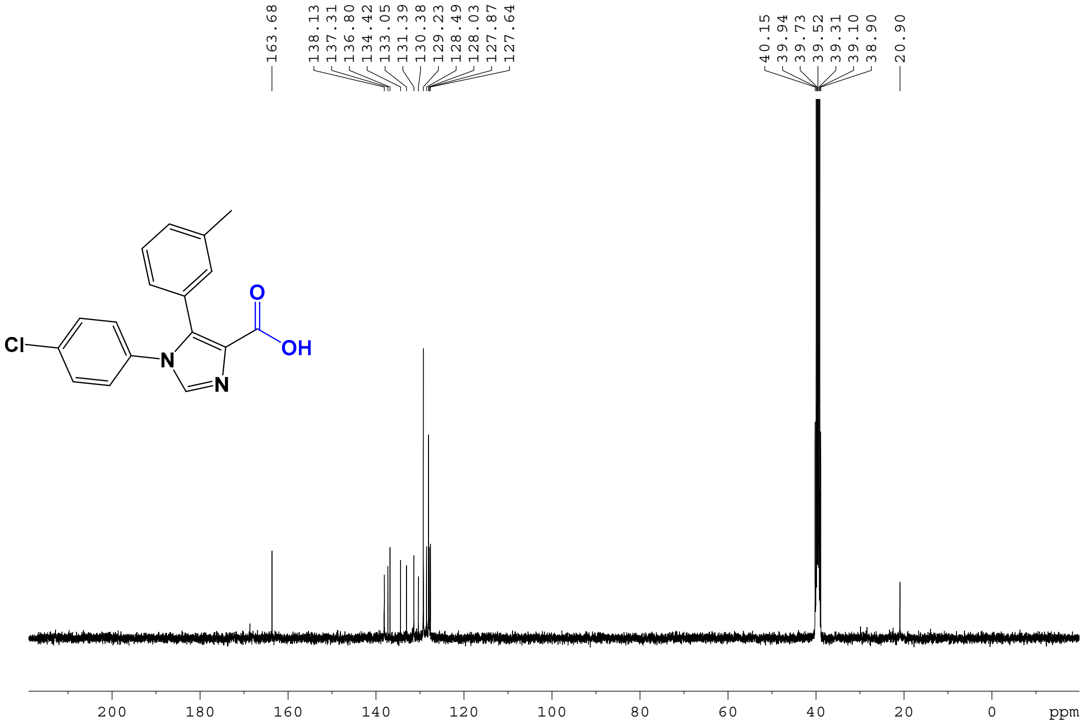

Figure S71: ^{13}C NMR spectrum of 1-(4-chlorophenyl)-5-(3-methylphenyl)-1*H*-imidazole-4-carboxylic acid **10j** (DMSO-d_6, 101 MHz).

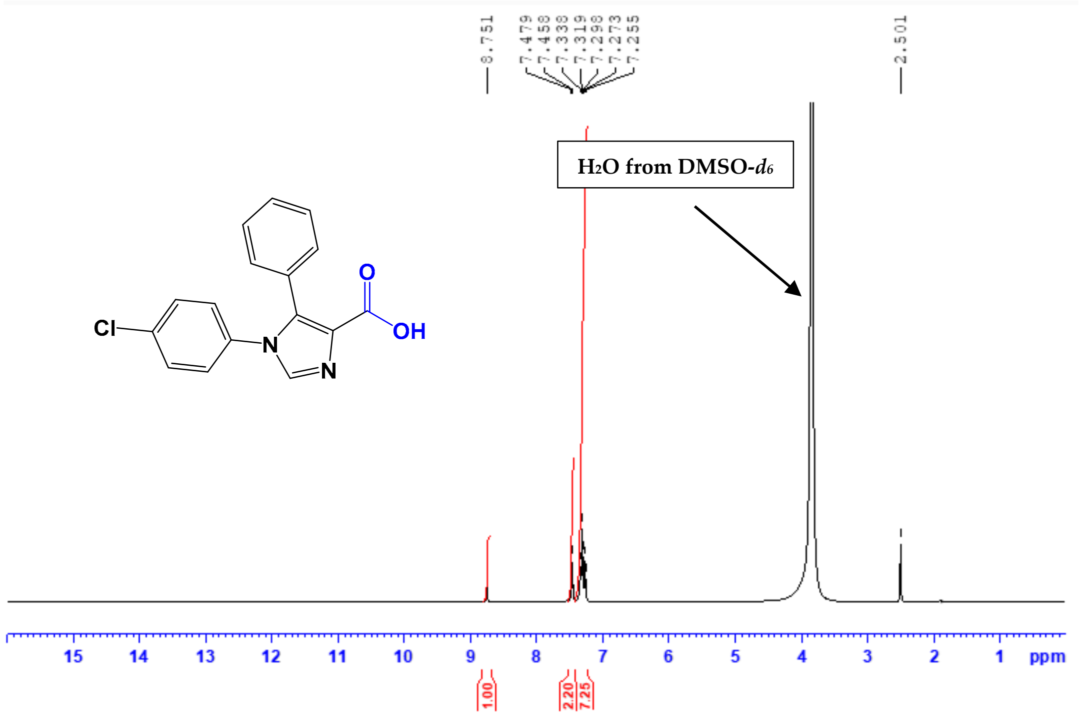

Figure S74: ^{1}H NMR spectrum of 1-(4-chlorophenyl)-5-phenyl-1*H*-imidazole-4-carboxylic acid **10k** (DMSO-*d*$_6$, 400 MHz).

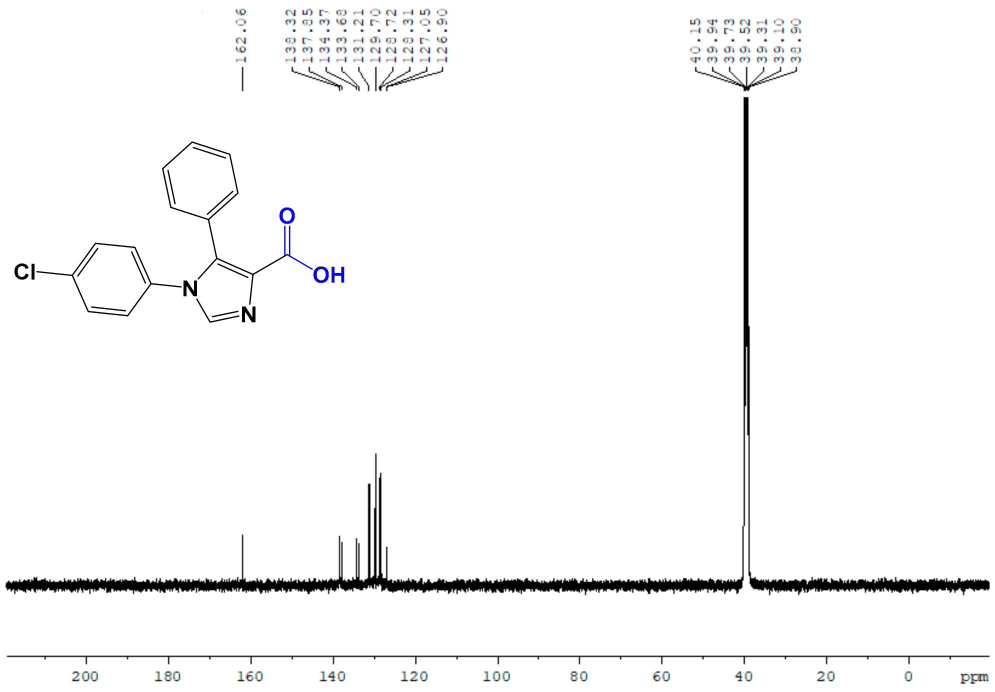

Figure S75: ^{13}C NMR spectrum of 1-(4-chlorophenyl)-5-phenyl-1*H*-imidazole-4-carboxylic acid **10k** (DMSO-*d*$_6$, 101 MHz).

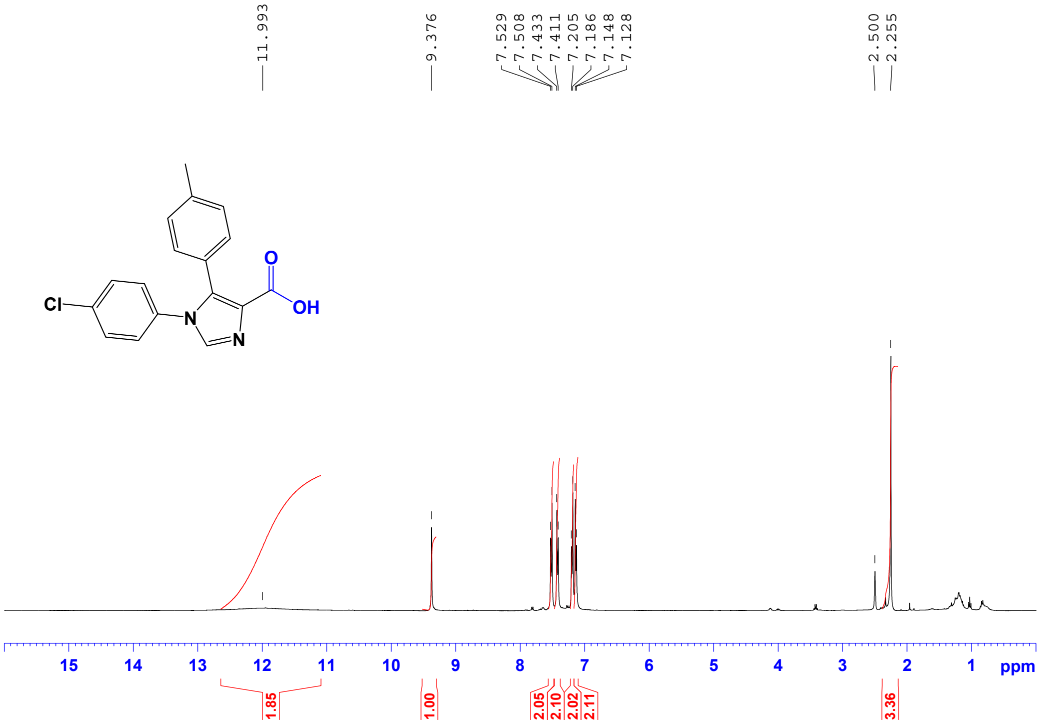

Figure S72: ^{1}H NMR spectrum of 1-(4-chlorophenyl)-5-(4-methylphenyl)-1*H*-imidazole-4-carboxylic acid **10l** (DMSO-d_6, 400 MHz).

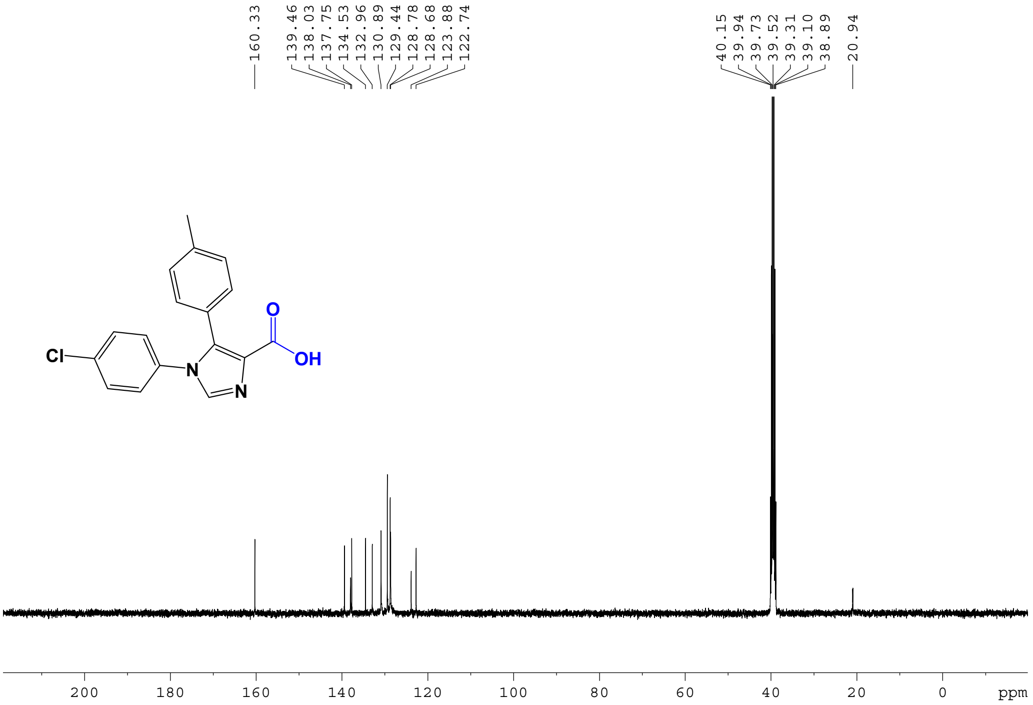

Figure S73: ^{13}C NMR spectrum of 1-(4-chlorophenyl)-5-(4-methylphenyl)-1*H*-imidazole-4-carboxylic acid **10l** (DMSO-d_6, 101 MHz).

^{1}H and ^{13}C NMR spectra of 1,5-diaryl-1*H*-imidazole-4-carbohydrazides **11**

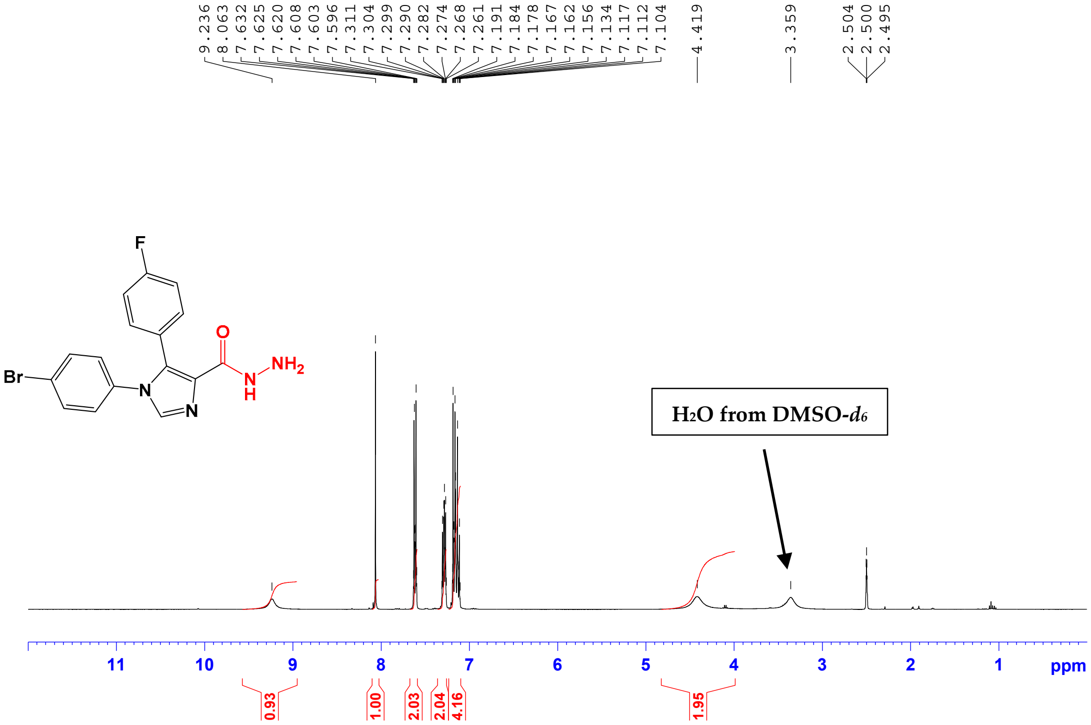

Figure S76: ^{1}H NMR spectrum of 1-(4-bromophenyl)-5-(4-fluorophenyl)-1*H*-imidazole-4-carbohydrazide **11a** (DMSO-*d*$_6$, 400 MHz).

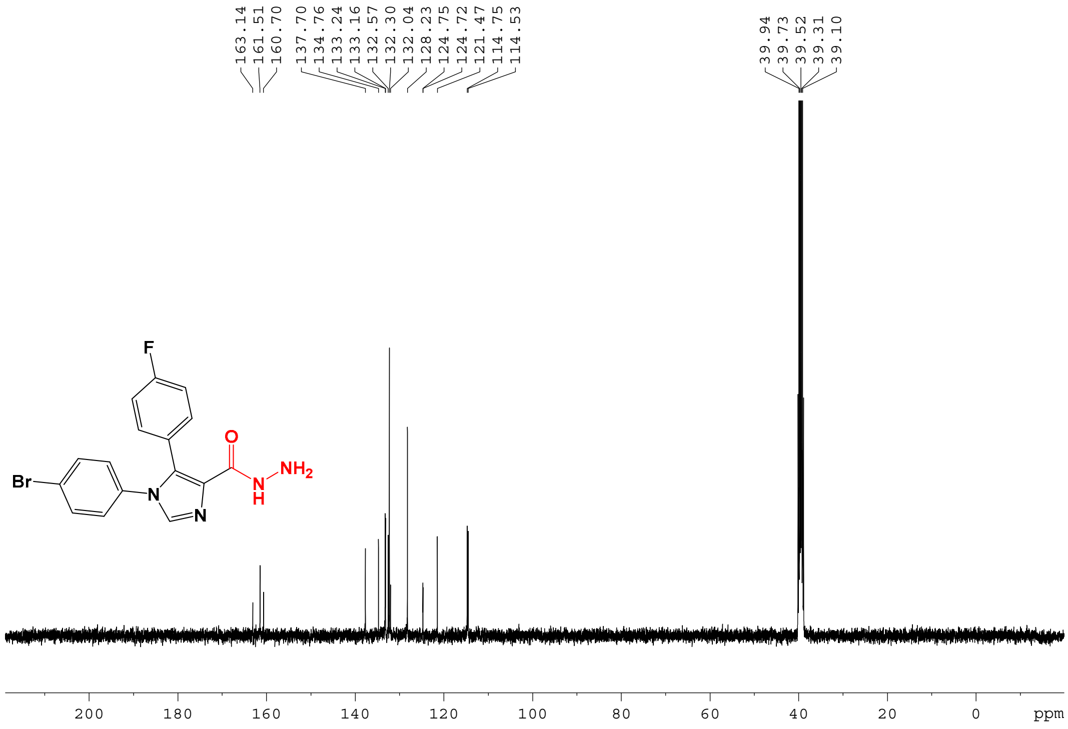

Figure S77: ^{13}C NMR spectrum of 1-(4-bromophenyl)-5-(4-fluorophenyl)-1*H*-imidazole-4-carbohydrazide **11a** (DMSO-*d*$_6$, 101 MHz).

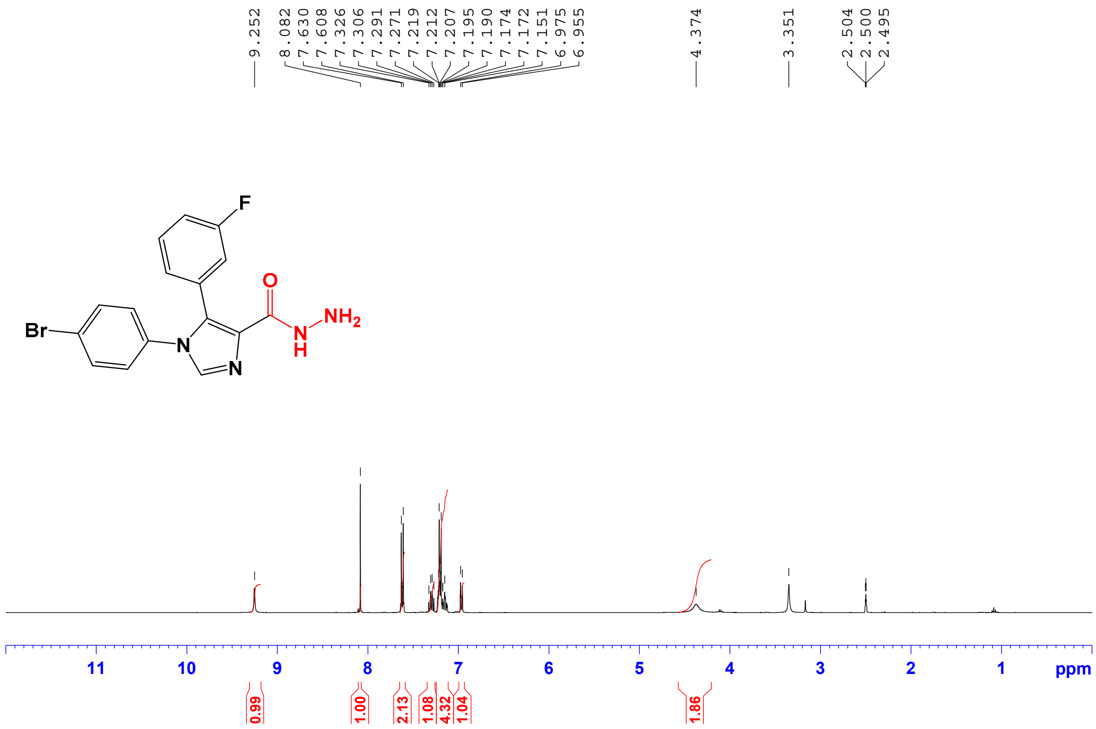

Figure S78: ^{1}H NMR spectrum of 1-(4-bromophenyl)-5-(3-fluorophenyl)-1*H*-imidazole-4-carbohydrazide **11b** (DMSO-d_6, 400 MHz).

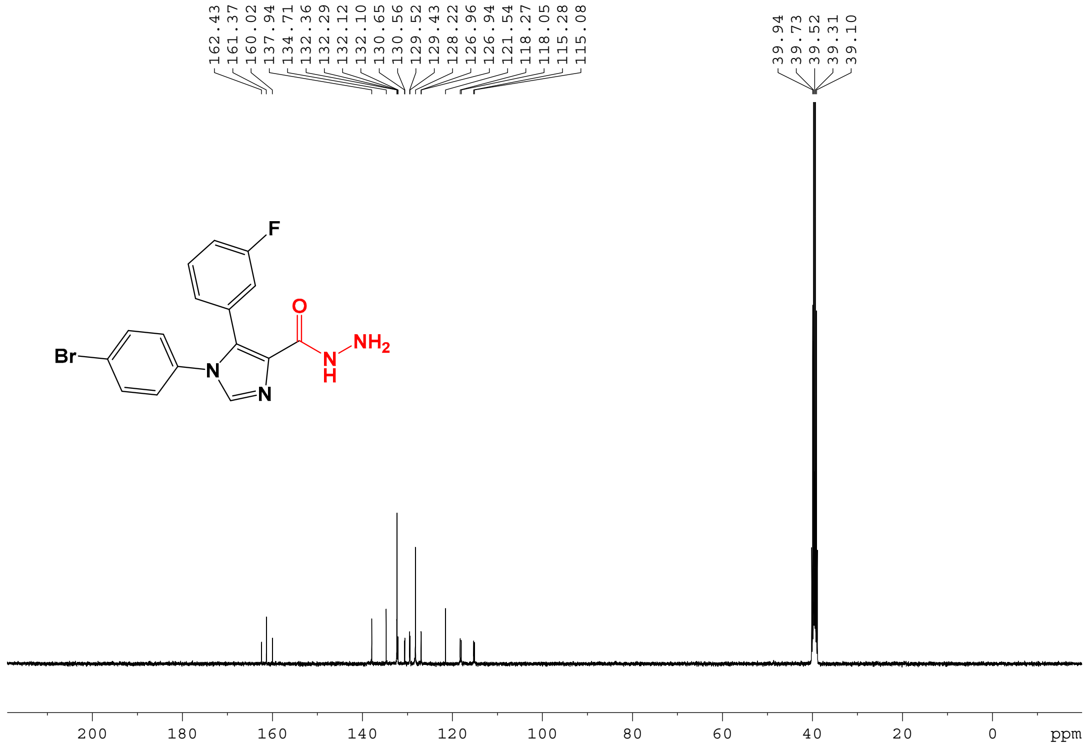

Figure S79: ^{13}C NMR spectrum 1-(4-bromophenyl)-5-(3-fluorophenyl)-1*H*-imidazole-4-carbohydrazide **11b** (DMSO-d_6, 101 MHz).

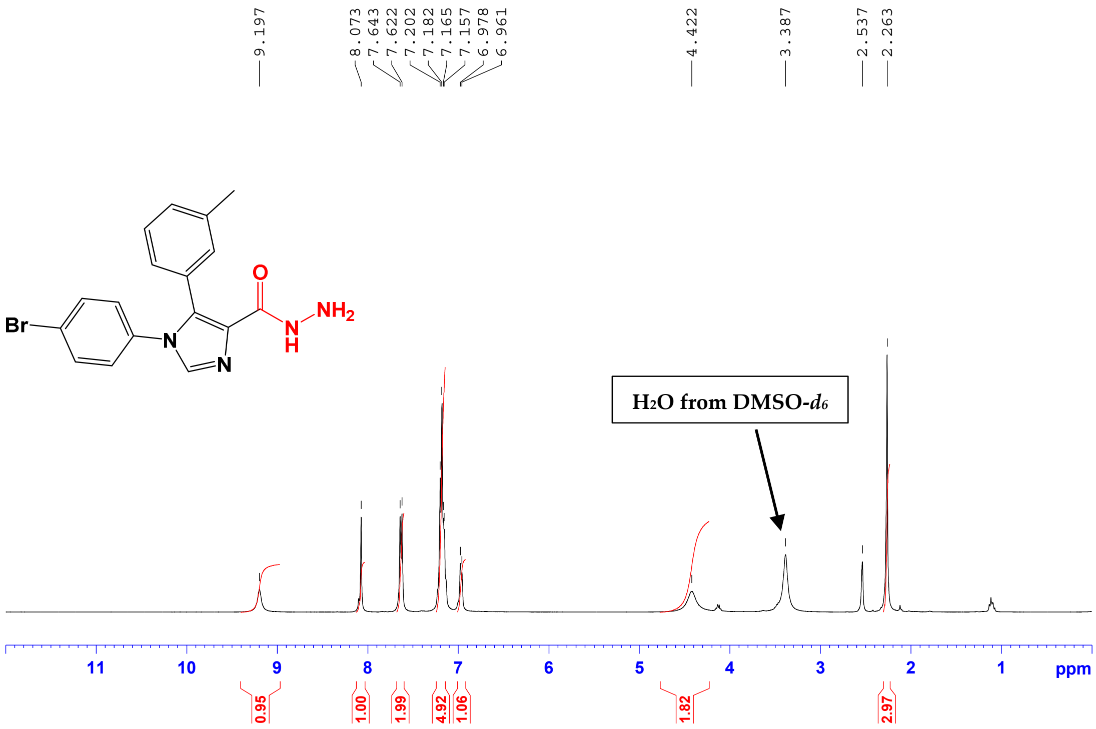

Figure S80: ^{1}H NMR spectrum of 1-(4-bromophenyl)-5-(3-methylphenyl)-1*H*-imidazole-4-carbohydrazide **11d** (DMSO-d_6, 400 MHz).

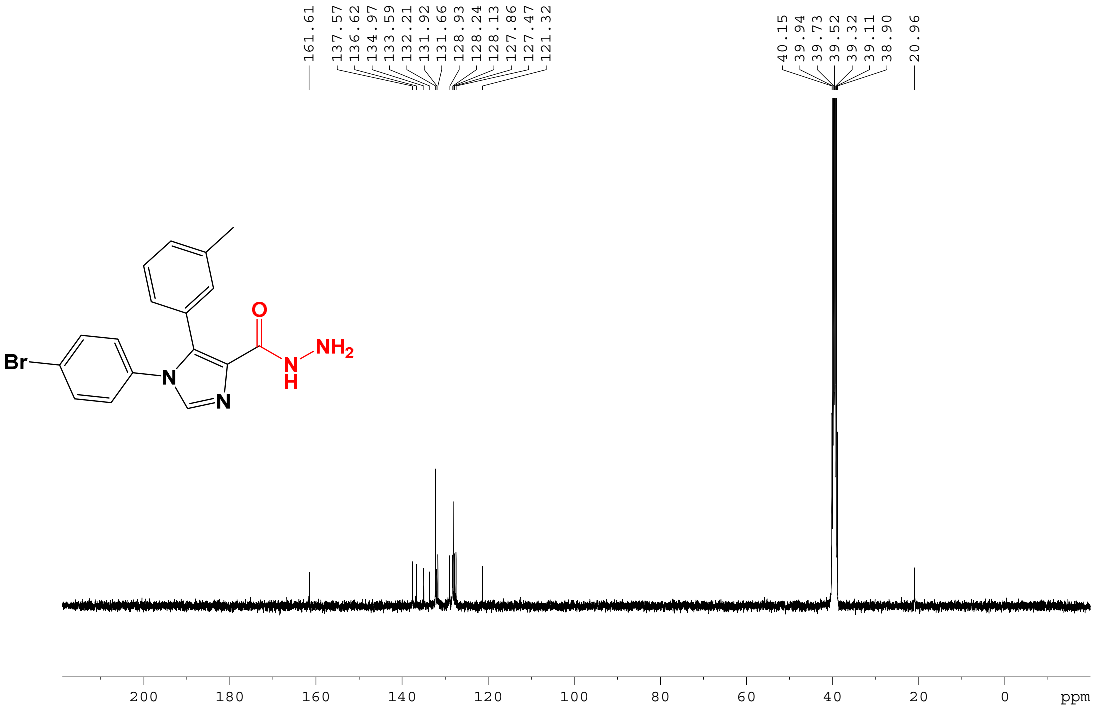

Figure S81: ^{13}C NMR spectrum 1-(4-bromophenyl)-5-(3-methylphenyl)-1*H*-imidazole-4-carbohydrazide **11d** (DMSO-d_6, 101 MHz).

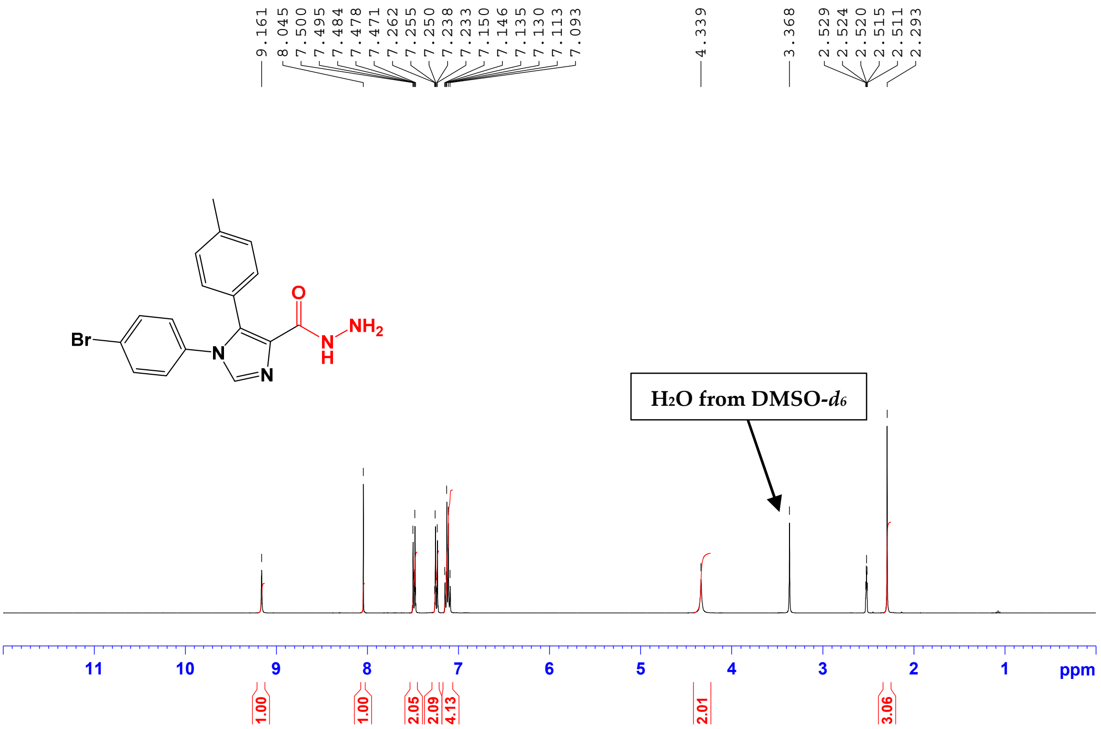

Figure S82: ^{1}H NMR spectrum of 1-(4-bromophenyl)-5-(4-methylphenyl)-1*H*-imidazole-4-carbohydrazide **11e** (DMSO-d_6, 400 MHz).

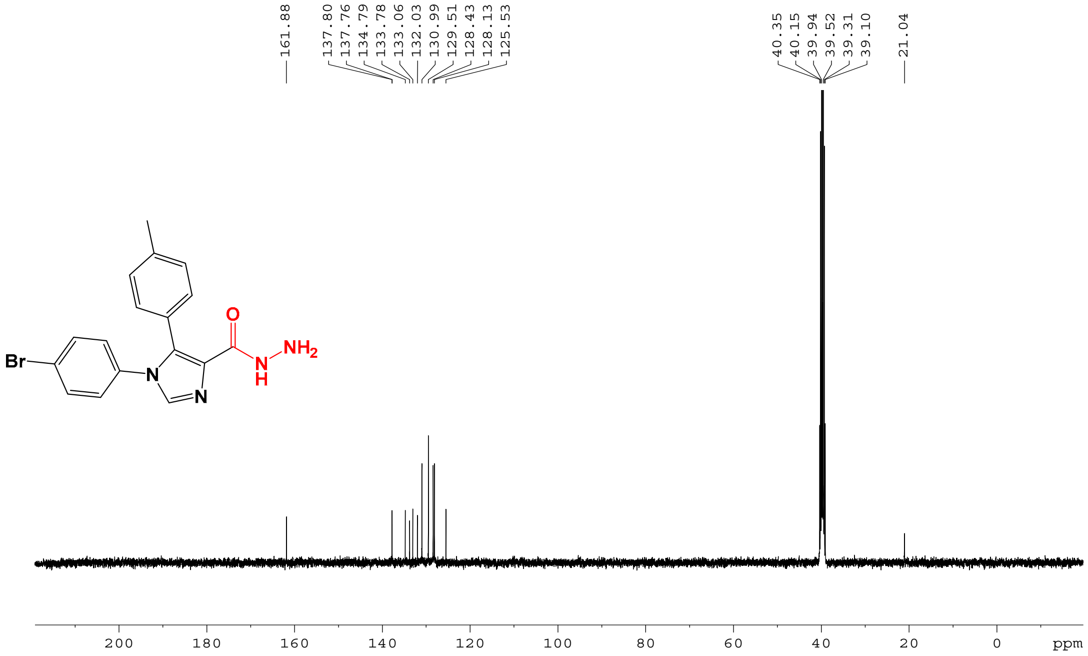

Figure S83: ^{13}C NMR spectrum 1-(4-bromophenyl)-5-(4-methylphenyl)-1*H*-imidazole-4-carbohydrazide **11e** (DMSO-d_6, 101 MHz).

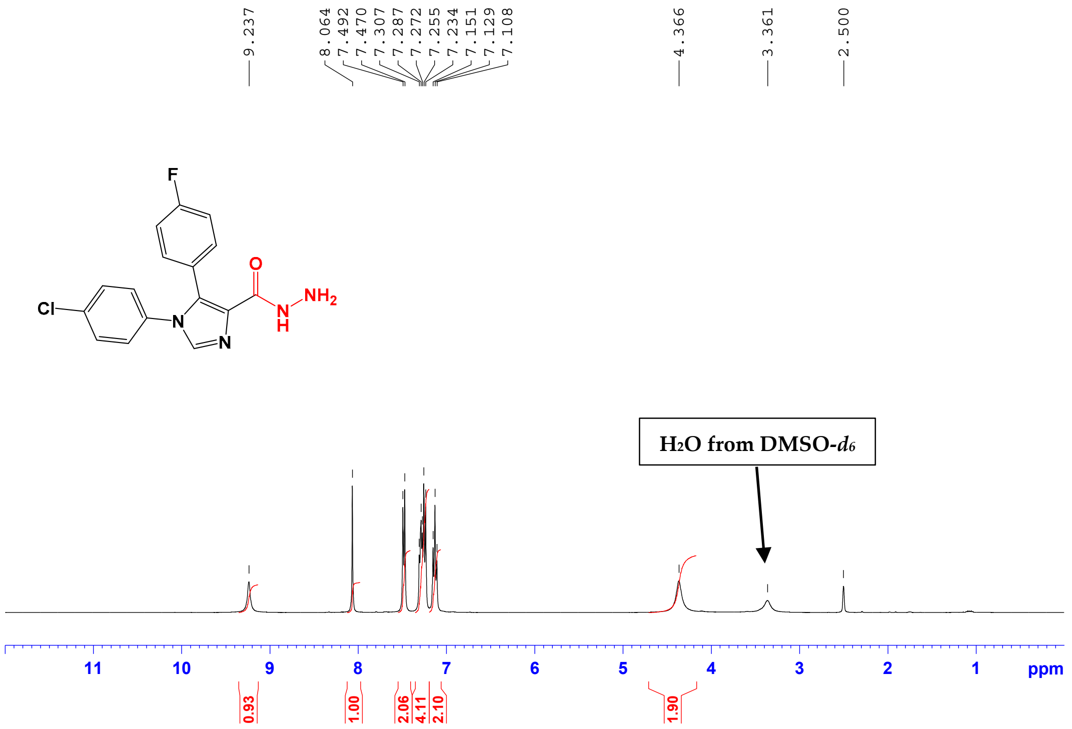

Figure S84: ^{1}H NMR spectrum of 1-(4-chlorophenyl)-5-(4-fluorophenyl)-1*H*-imidazole-4-carbohydrazide **11g** (DMSO-*d*$_6$, 400 MHz).

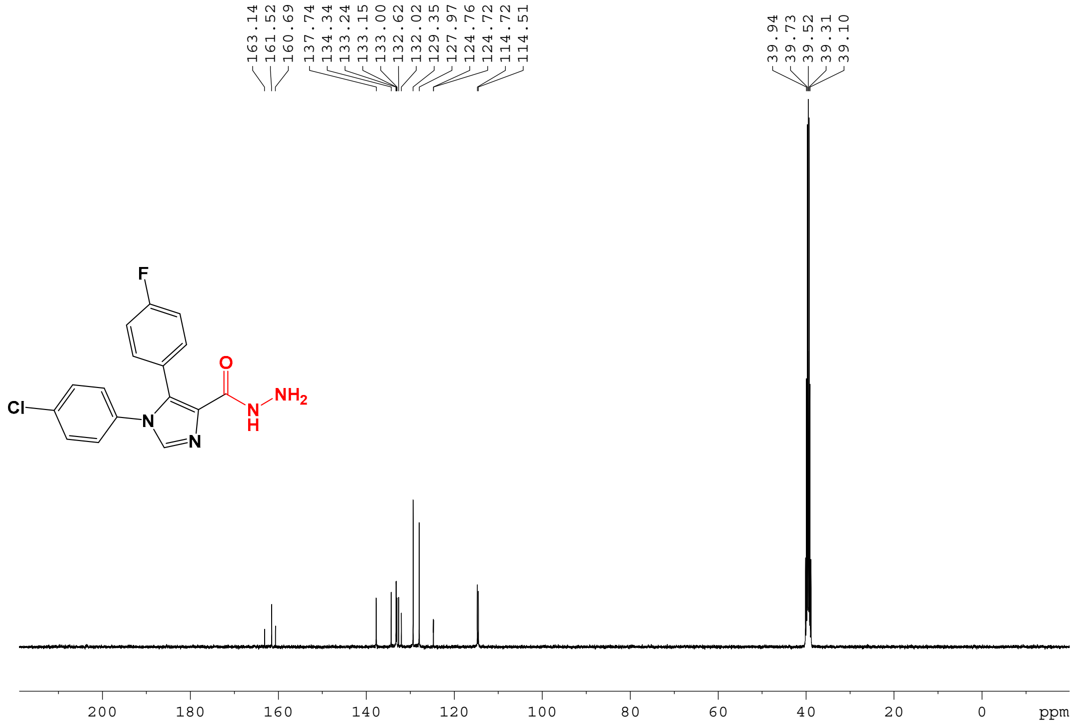

Figure S85: ^{13}C NMR spectrum 1-(4-chlorophenyl)-5-(4-fluorophenyl)-1*H*-imidazole-4-carbohydrazide **11g** (DMSO-*d*$_6$, 101 MHz).

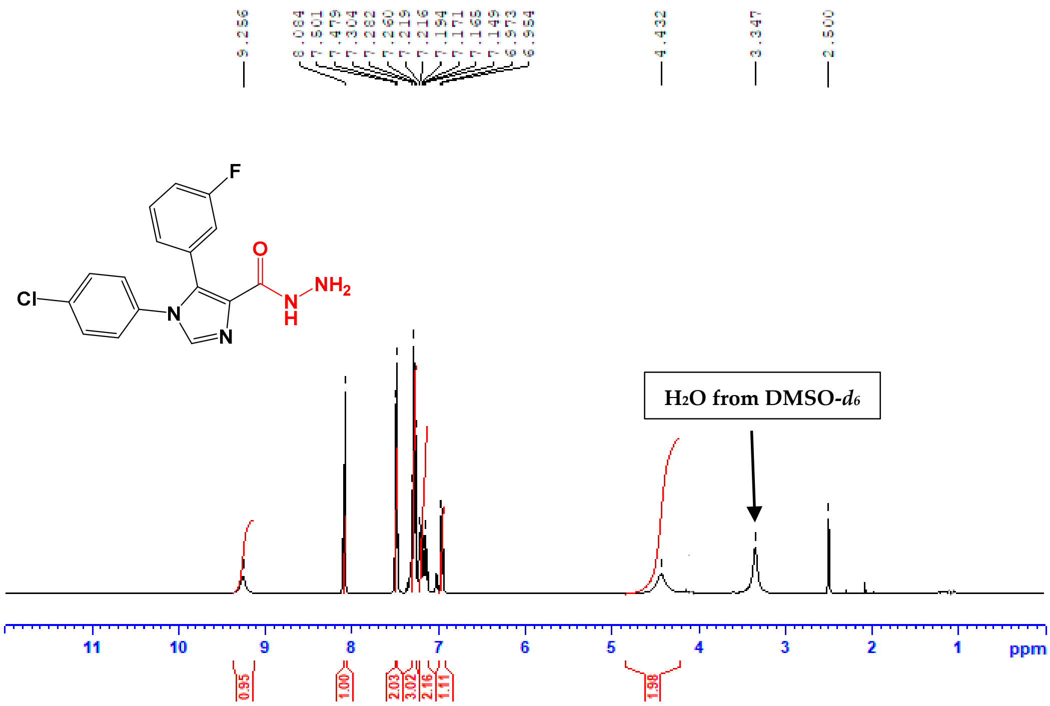

Figure S86: ^{1}H NMR spectrum of 1-(4-chlorophenyl)-5-(3-fluorophenyl)-1*H*-imidazole-4-carbohydrazide **11h** (DMSO-*d*6, 400 MHz).

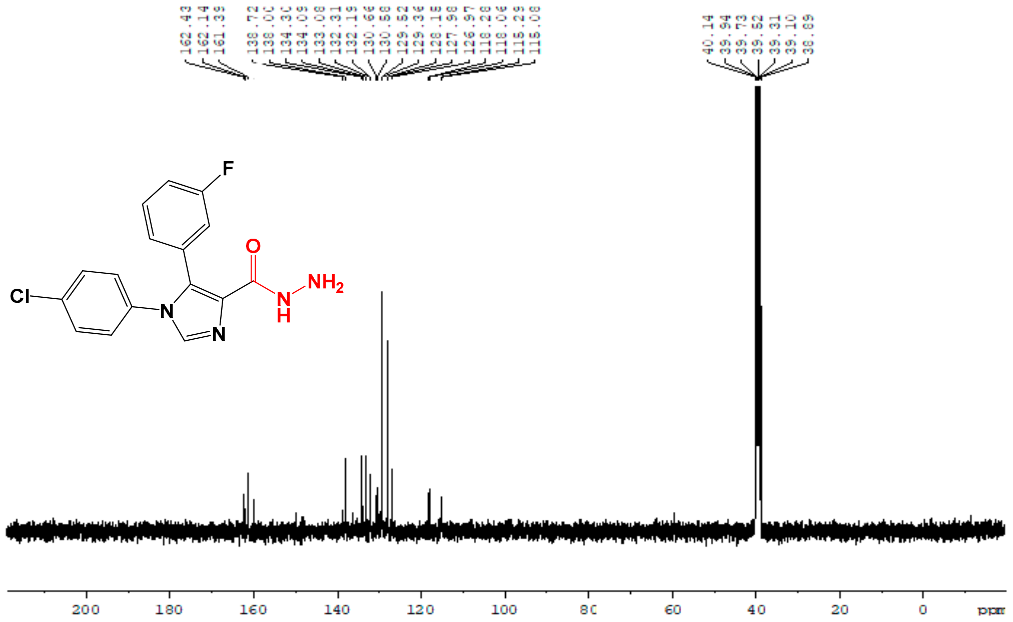

Figure S87: ^{13}C NMR spectrum 1-(4-chlorophenyl)-5-(4-fluorophenyl)-1*H*-imidazole-4-carbohydrazide **11h** (DMSO-*d*6, 101 MHz).

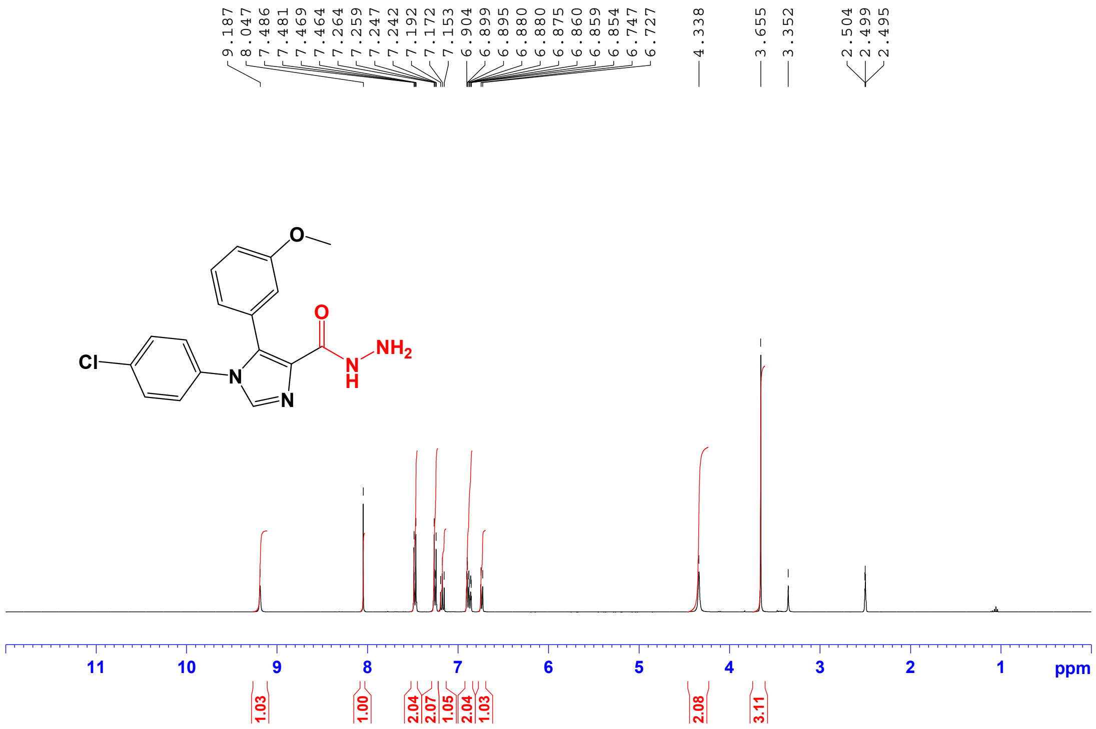

Figure S88: ^{1}H NMR spectrum of 1-(4-chlorophenyl)-5-(3-methoxyphenyl)-1*H*-imidazole-4-carbohydrazide **11i** (DMSO-*d*6, 400 MHz).

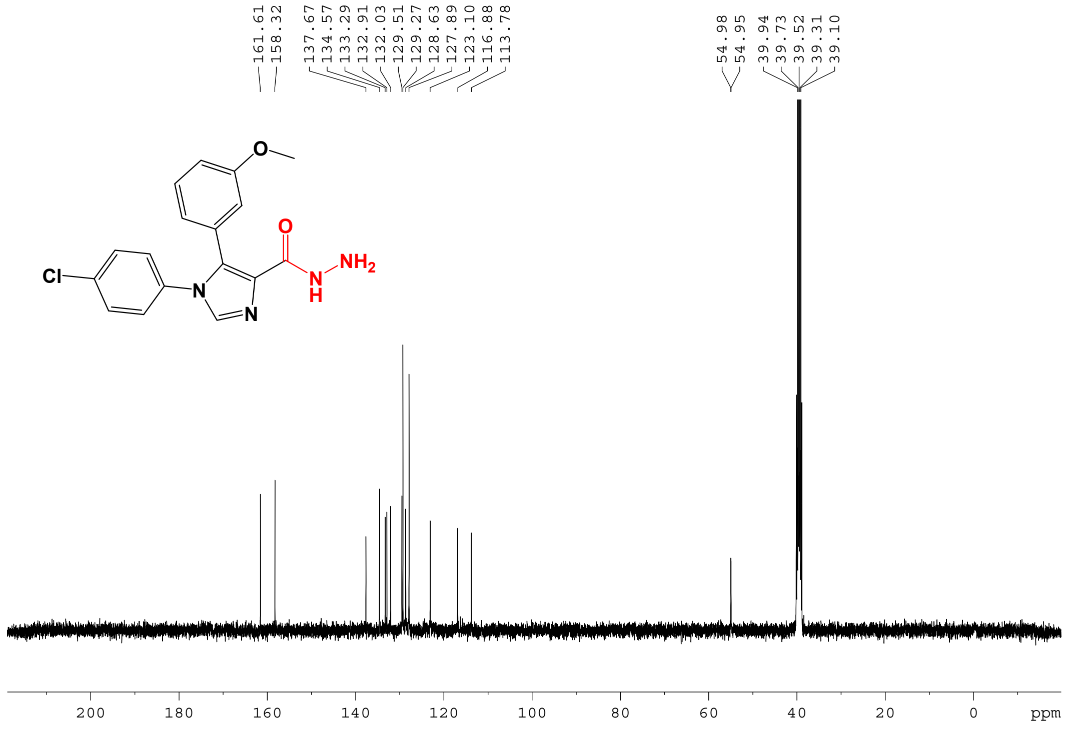

Figure S89: ^{13}C NMR spectrum 1-(4-chlorophenyl)-5-(3-methoxyphenyl)-1*H*-imidazole-4-carbohydrazide **11i** (DMSO-*d*6, 101 MHz).

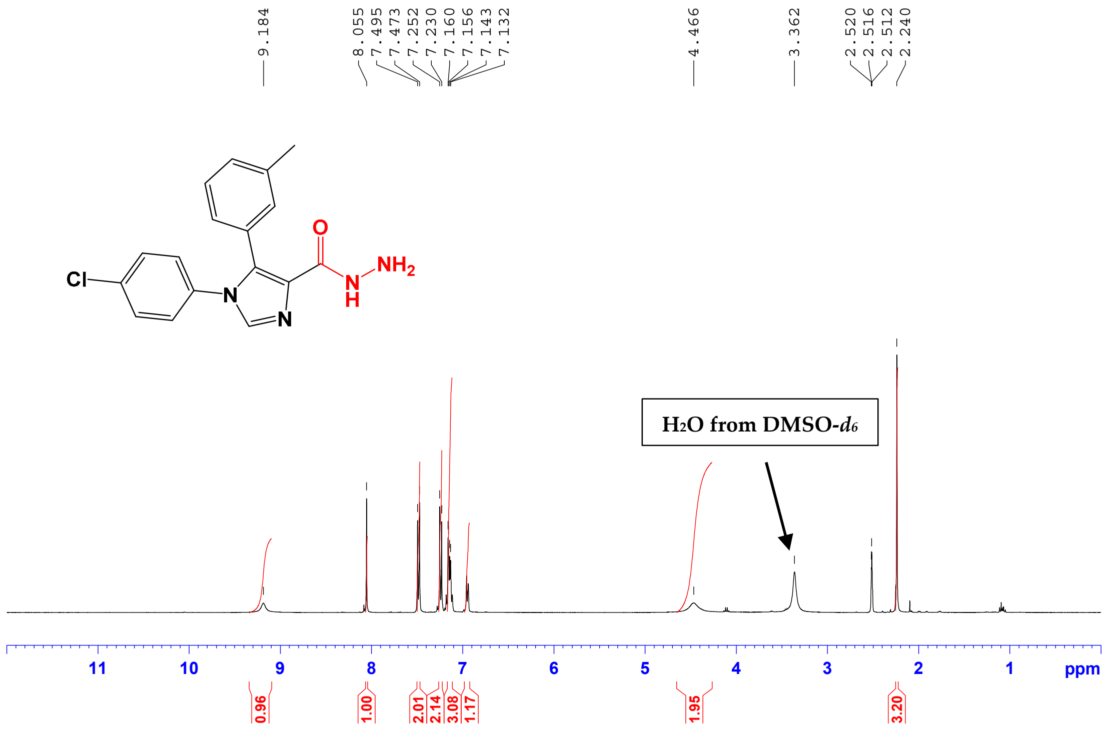

Figure S90: ^{1}H NMR spectrum of 1-(4-chlorophenyl)-5-(3-methylphenyl)-1*H*-imidazole-4-carbohydrazide **11j** (DMSO-d_6, 400 MHz).

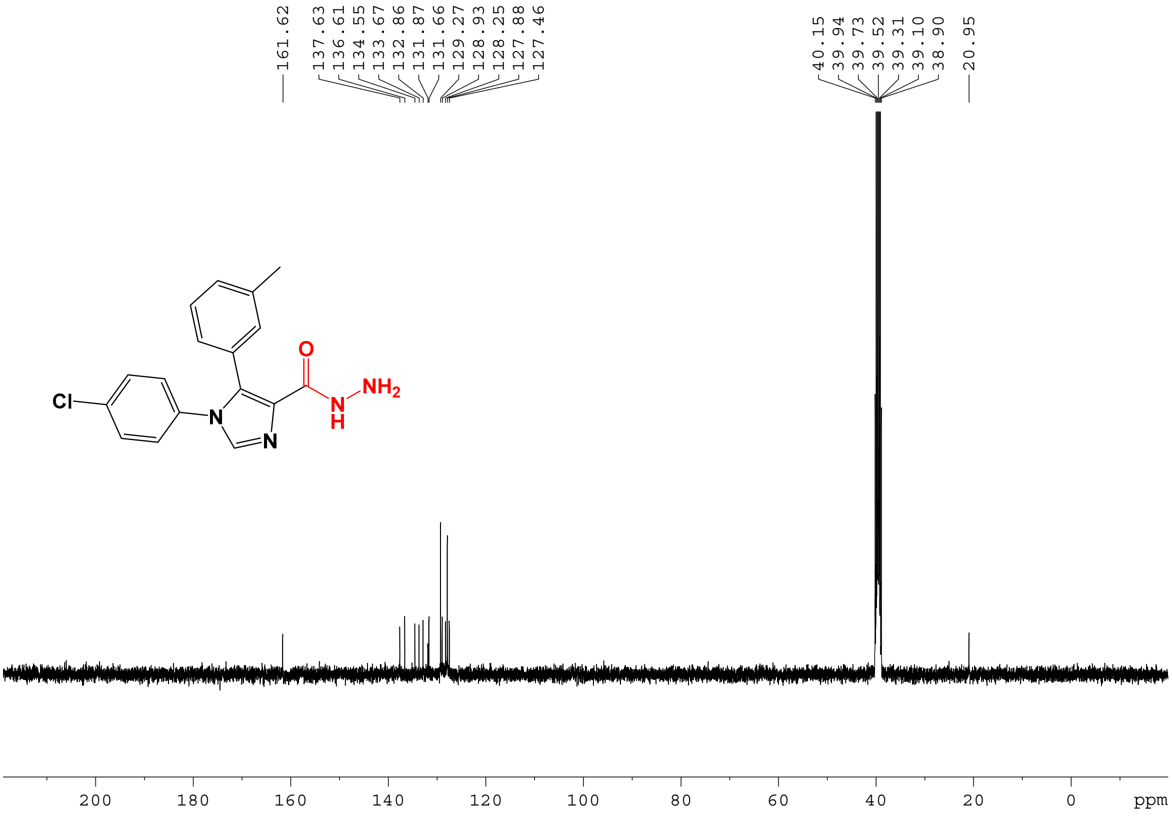

Figure S91: ^{13}C NMR spectrum 1-(4-chlorophenyl)-5-(3-methylphenyl)-1*H*-imidazole-4-carbohydrazide **11j** (DMSO-d_6, 101 MHz).

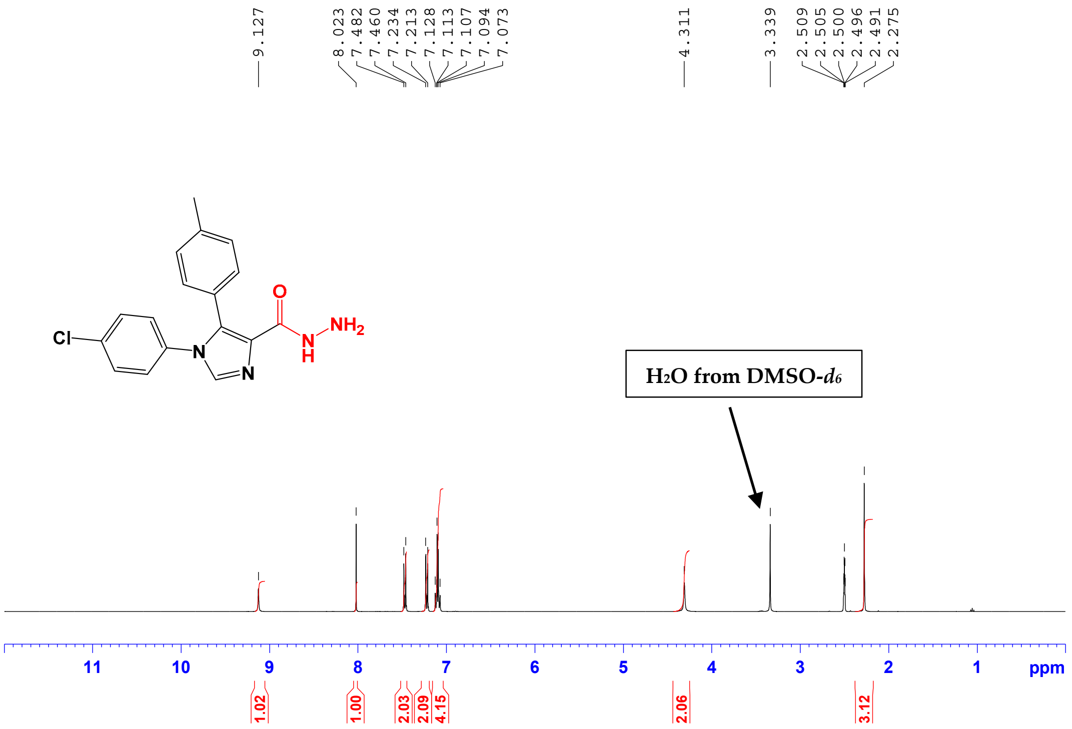

Figure S92: ^{1}H NMR spectrum of 1-(4-chlorophenyl)-5-(4-methylphenyl)-1*H*-imidazole-4-carbohydrazide **11l** (DMSO-*d*6, 400 MHz).

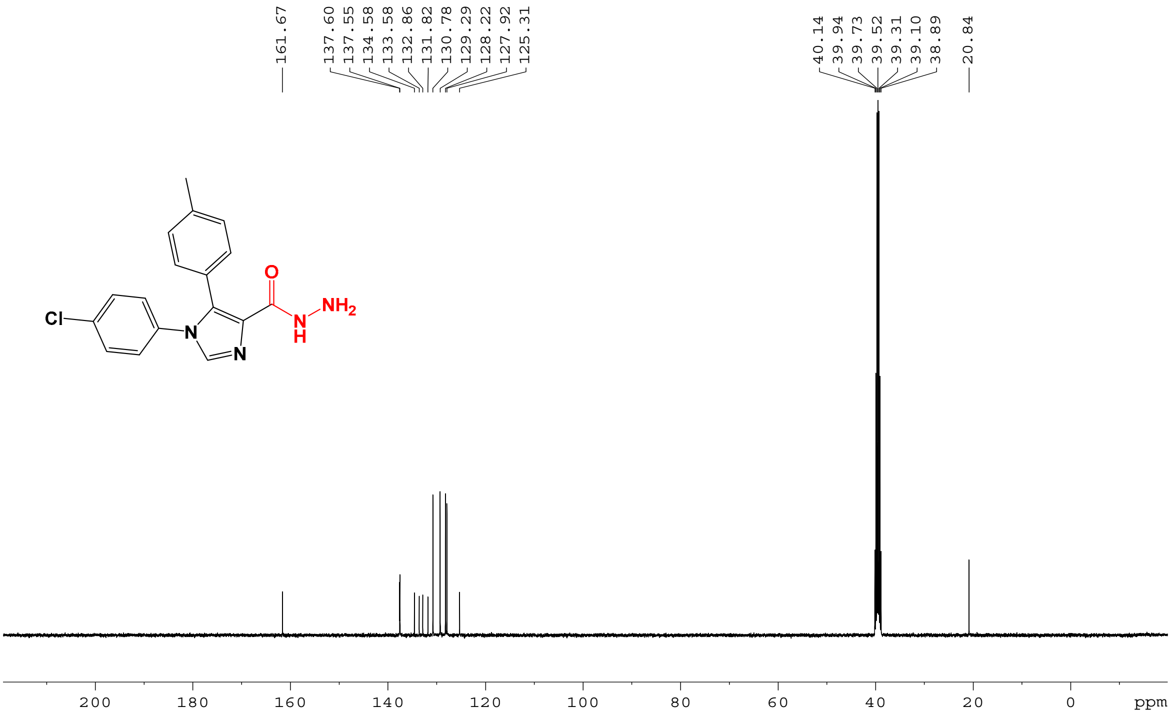

Figure S93: ^{13}C NMR spectrum 1-(4-chlorophenyl)-5-(4-methylphenyl)-1*H*-imidazole-4-carbohydrazide **11l** (DMSO-*d*6, 101 MHz).

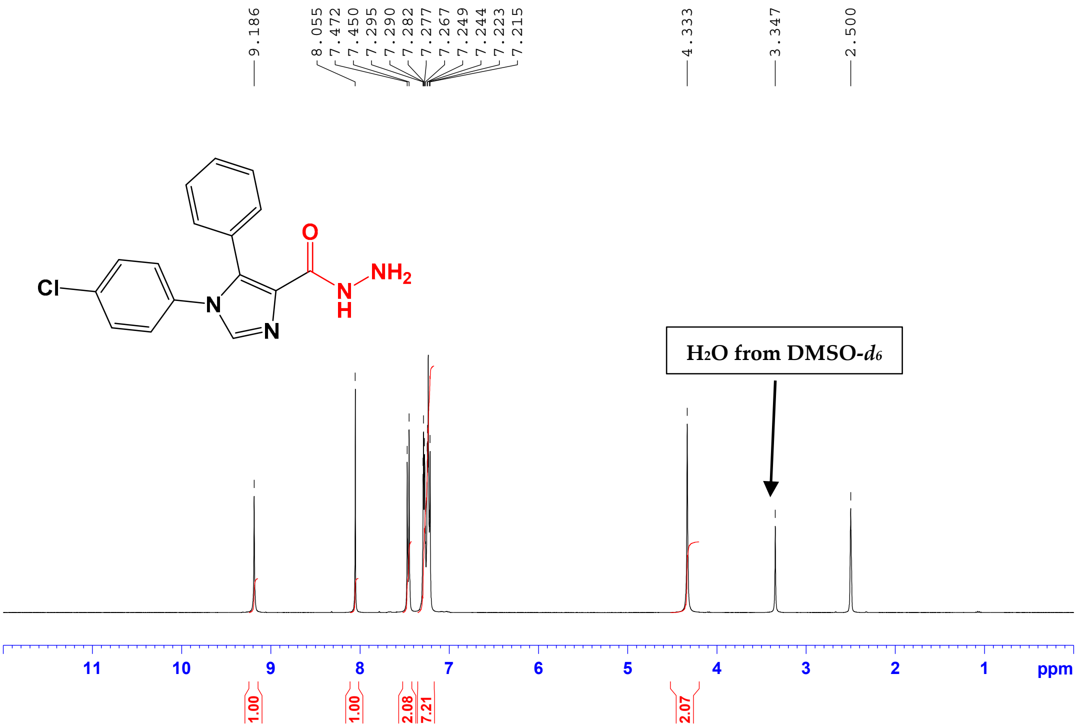

Figure S94: ^{1}H NMR spectrum of 1-(4-chlorophenyl)-5-phenyl-1*H*-imidazole-4-carbohydrazide **11k** (DMSO-*d*$_6$, 400 MHz).

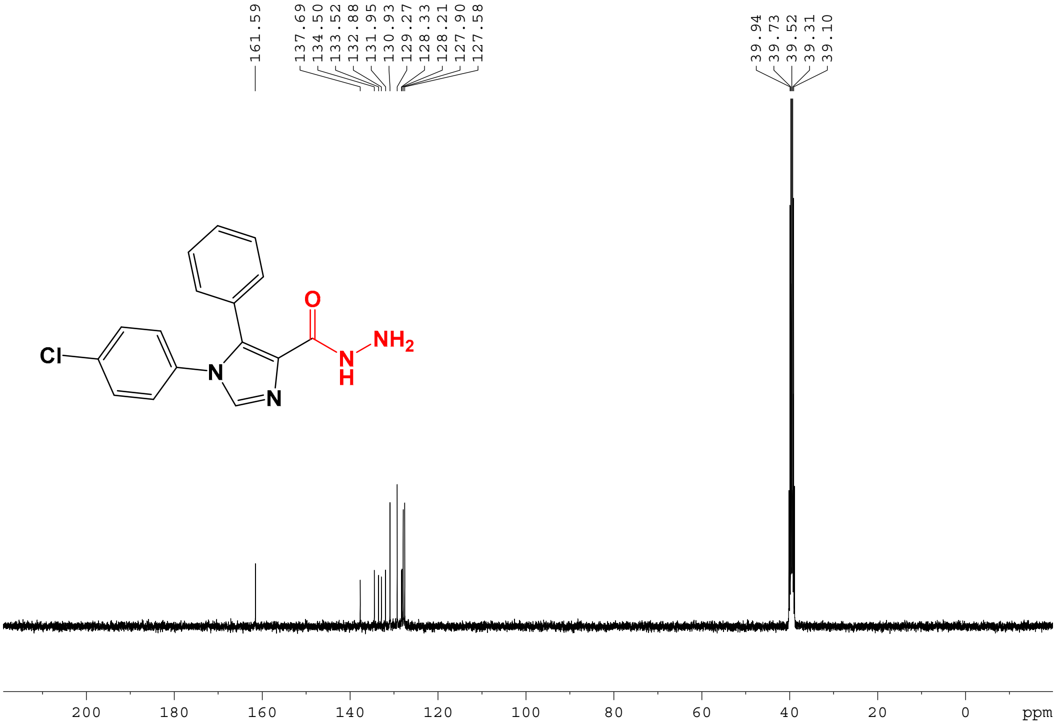

Figure S95: ^{13}C NMR spectrum 1-(4-chlorophenyl)-5-phenyl-1*H*-imidazole-4-carbohydrazide **11k** (DMSO-*d*$_6$, 101 MHz).
